# UV Light Reveals the Diversity of Jurassic Shell Colour Patterns: Examples from the Cordebugle Lagerstätte (Calvados, France)

**DOI:** 10.1371/journal.pone.0126745

**Published:** 2015-06-03

**Authors:** Bruno Caze, Didier Merle, Simon Schneider

**Affiliations:** 1 Département Histoire de la Terre, Sorbonne Universités (CR2P—MNHN, CNRS, UPMC-Paris6), Paris, France; 2 CASP, University of Cambridge, Cambridge, United Kingdom; Universität Göttingen, GERMANY

## Abstract

Viewed under UV light the diverse and exceptionally well-preserved molluscs from the Late Jurassic Cordebugle Konservat Lagerstätte (Calvados, Normandy, France) reveal fluorescent fossil shell colour patterns predating the oldest previously known instance of such patterns by 100 Myr. Evidently, residual colour patterns are observable in Mesozoic molluscs by application of this non-destructive method, provided the shells are not decalcified or recrystallized. Among 46 species which are assigned to twelve gastropod families and eight bivalve families, no less than 25 species yielded positive results. Out of nine colour pattern morphologies that have been distinguished six occur in gastropods and three in bivalves. The presence of these variant morphologies clearly indicates a significant pre-Cenozoic diversification of colour patterns, especially in gastropods. In addition, the occurrence of two distinct types of fluorescence highlights a major difference in the chemical composition of the pigments involved in colour pattern formation in gastropods. This discovery enables us to discriminate members of higher clades, i.e. the Vetigastropoda emitting red fluorescence from the Caenogastropoda and Heterobranchia emitting whitish-beige to yellow fluorescence. Consequently, fluorescent colour patterns may help to allocate part of the numerous enigmatic Mesozoic gastropod taxa to their correct systematic position.

## Introduction

**Fig 1:**
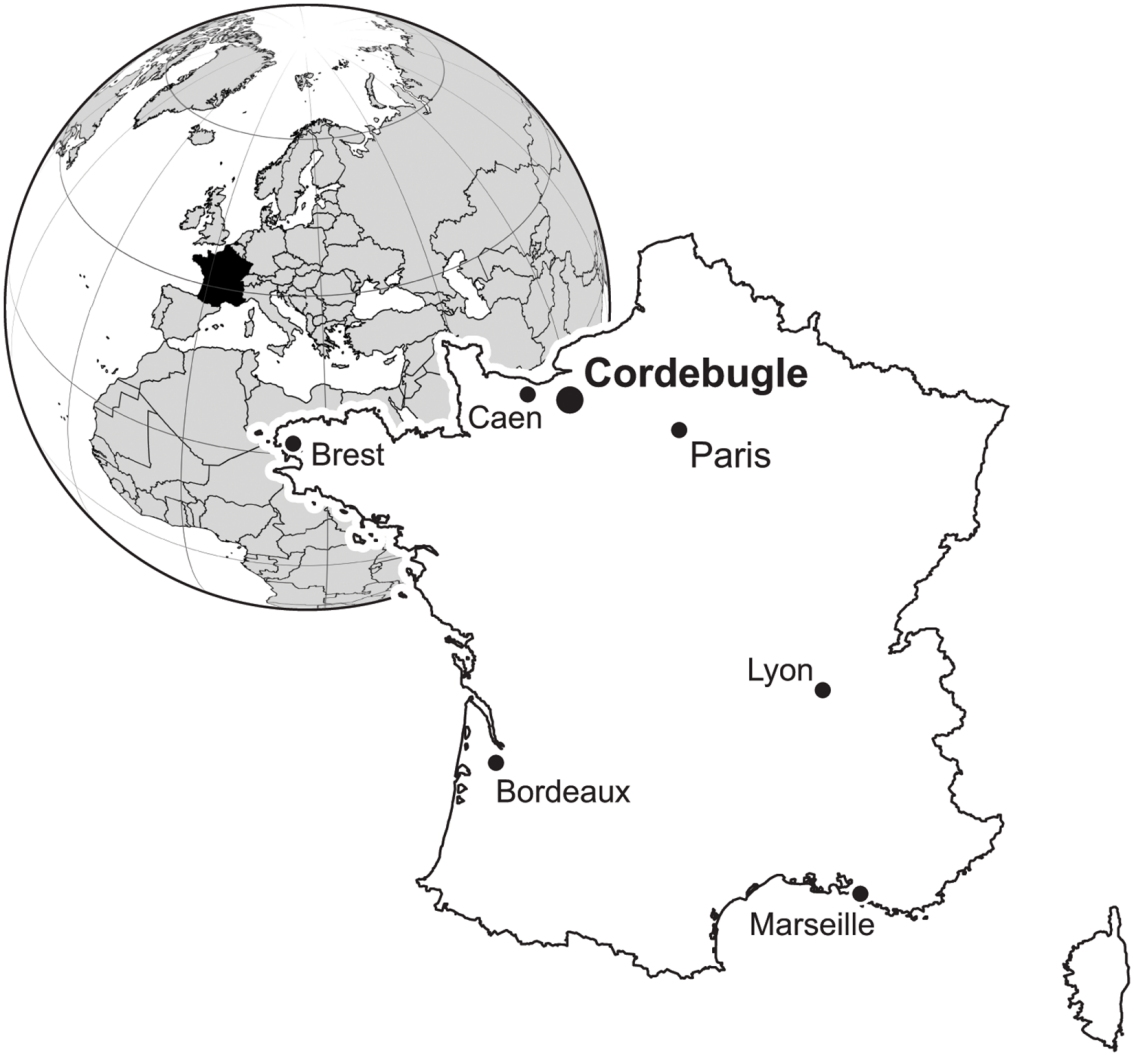
Geographic overview, showing the location of the Cordebugle site.

Colour patterns of animals play an important role in numerous ecological and evolutionary processes such as sexual selection, camouflage or UV-protection. It is difficult to document and consider colour pattern evolution through time, because most of the pigments involved in their formation decay easily and disappear very quickly after death. This is certainly the case among molluscs (Gastropoda, Bivalvia), several thousand Recent species of which display highly variable shell colour patterns, whereas most fossils seem devoid of them, apart from exceptionally well-preserved specimens showing remnants of patterns in natural light (e.g., [1–9]). A number of scholars, however, have demonstrated that masked patterns can be enhanced by bleaching Cenozoic shells in sodium hypochlorite and exposing them to UV light [10–17]. Furthermore, several recent large-scale surveys using this non-destructive method have shown residual patterns to be very common in Cenozoic shells from the Thanetian (late Palaeocene, 58 Myr) to the Quaternary [18–22]. Colour patterns in Mesozoic shells have not yet been investigated systematically using UV light, except for Miethe and Born (1928) [23], who only very briefly reported on three examples of fluorescent colour patterns in Mesozoic mollusc shells without providing figures: (1) an Early Jurassic ammonite, (2) a Triassic pectinid and (3) a Late Cretaceous gryphaeid oyster.

This lack of study is in part due to the relative scarcity of non-decalcified or non-recrystallized Mesozoic shells. On the other hand, the respective procedure simply has not yet been systematically applied to Mesozoic shells. The few paleontological sites that do potentially preserve shells retaining residual colour patterns may therefore provide key data for the understanding of the evolution of these colour patterns during the Mesozoic. The Oxfordian locality of Cordebugle (Calvados, Normandy, France; 157 Myr) is of prime importance with regard to this subject, since it preserves a highly diversified mollusc fauna with many aragonitic shells retaining their original mineralogy [24–32]. The revelation of colour patterns under UV light is generally controlled by the quality of the preservation of the original shell mineralogy [19], both in aragonite [17, 20, 33] and calcite [11]. Recrystallization of aragonite to calcite during diagenesis destroys the UV responsive residual pigments, although some residual pigments may still be observed in natural light in recrystallized shells, but do not emit fluorescence. Examples are *Ampullina perusta* (Defrance in Brongniart, 1823) from the Lutetian of Ronca (Italy) [20] or *Platyceras deceptivum* (Barrande in Perner, 1911) from the Ludlow of Na Požárech (Czech Republic) [3].

The aim of the present study is to: (1) systematically investigate the shells from the late Jurassic of Cordebugle under UV light, and detect and document any residual colour patterns; (2) provide an illustrated overview of all residual colour patterns found in the molluscs from Cordebugle; (3) demonstrate that this kind of preservation is basically not scarce in material from Mesozoic sediments, provided that the shells are well preserved; and (4) show that the type of fluorescence and variant morphologies of colour patterns may serve as additional characters for refined systematic assignment and determination of the studied species.

### Geographical Location and Geological Setting

The village of Cordebugle is located between Lisieux and Bernay (Normandie) and its famous fossiliferous outcrop was situated to the west of the village, near the farm La Martinière (Fig 1) [27]. The site became famous thanks to Alexandre Pierre Désiré Bigot, former professor at the University of Caen, who conducted extensive field studies at Cordebugle in 1892, and collected a rich and well preserved mollusc fauna from the Oxfordian Sables de Glos Formation. Subsequently, the site was overgrown by vegetation until it was re-accessed by André Chavan in the 1940s, who carried out additional field studies. Today, the locality is lost again, because of urban development (D. Raynaud personnal communication).

The Sables de Glos Formation is subdivided into two transgressive-regressive, fluvio-marine depositional sequences. The first sequence (10 metres thick), termed Sables de Glos inférieurs, is marked at its base by a transgressive conglomerate reworking the underlying Calcaire de Blangy with *Paracidaris florigemma*. Above this, the sequence displays an alternation of sands, sandstones and some ferruginous oolite layers. The lower part of this succession contains shallow-water marine fossils (molluscs, calcareous algae) mixed with fragments of terrestrial vegetation. This sequence is terminated by the Violet Bed representing an emergent horizon, which has undergone pedogenic alteration and gullying [34–35]. The second sequence (10 metres thick), termed Sables de Glos supérieurs, fills a large WSW-ENE oriented channel, that has been cut into the Sables de Glos inférieurs and was well exposed at Cordebugle. The base of this sequence comprises reworked blocks of the Violet Bed, sandy slabs, ferruginous nodules, fragments of continental lignites and marine molluscs. Above, an alternation of sandstones, sands, clays, and limestones, with frequent indicators of emergence, display beach ripples associated with tracks or burrows of crustaceans and small reptile footprints, desiccation cracks, migrating ripple structures associated with lenses of plant debris, and bones of marine and terrestrial vertebrates [34–36]. Some intercalated lenses of shelly marine sand yield a rich and exceptionally well-preserved bivalve and gastropod assemblage. Altogether more than 100 species have been described from Cordebugle [24–32]. Towards the top of the sequence, microconglomeratic intervals reveal an elevation of hydrodynamic energy on the sea bottom [34]. The Sables de Glos supérieurs are limited by an erosive surface and are overlain by Aptian ferruginous sandstones.

Following Rioult (1980) [37], the ammonite *Amoeboceras glosense* (Bigot and Brasil, 1904) found in the Sables de Glos inférieurs indicates the Cautisnigrae Biozone and the Glosense Subzone of the Boreal Province corresponding to the earliest Late Oxfordian. The Sables de Glos supérieurs yielded *Euaspidoceras striatocostatum* (Dorn, 1931) [34] which is indicative of the Bimammatum Biozone and the Semiarmatum Subzone of the Mediterranean province [38]. This subzone is correlated to the Regulare Biozone of the Boreal Province and thus points to a middle Late Oxfordian age.

## Material and Methods

### Studied material

Since the Cordebugle site is now inaccessible, the only available material comes from earlier collections. We have studied specimens from the historical Cossmann, Bigot and de Morgan collections housed at the Muséum national d’Histoire naturelle, Paris, collection de Paléontologie (MNHN.F), and from the Le Marchand and Curet collections housed at the Université Pierre et Marie Curie, Sorbonne Universités, collection de Paléontologie (UPMC). The study is based on 575 specimens belonging to 28 species of gastropods and 18 species of bivalves. The specimen numbers for all samples are given in the Results—Systematic survey for every studied species at the entry Examined material. In addition, a detailed list of specimens is provided in S1 Table.

### Experimental design

**Fig 2:**
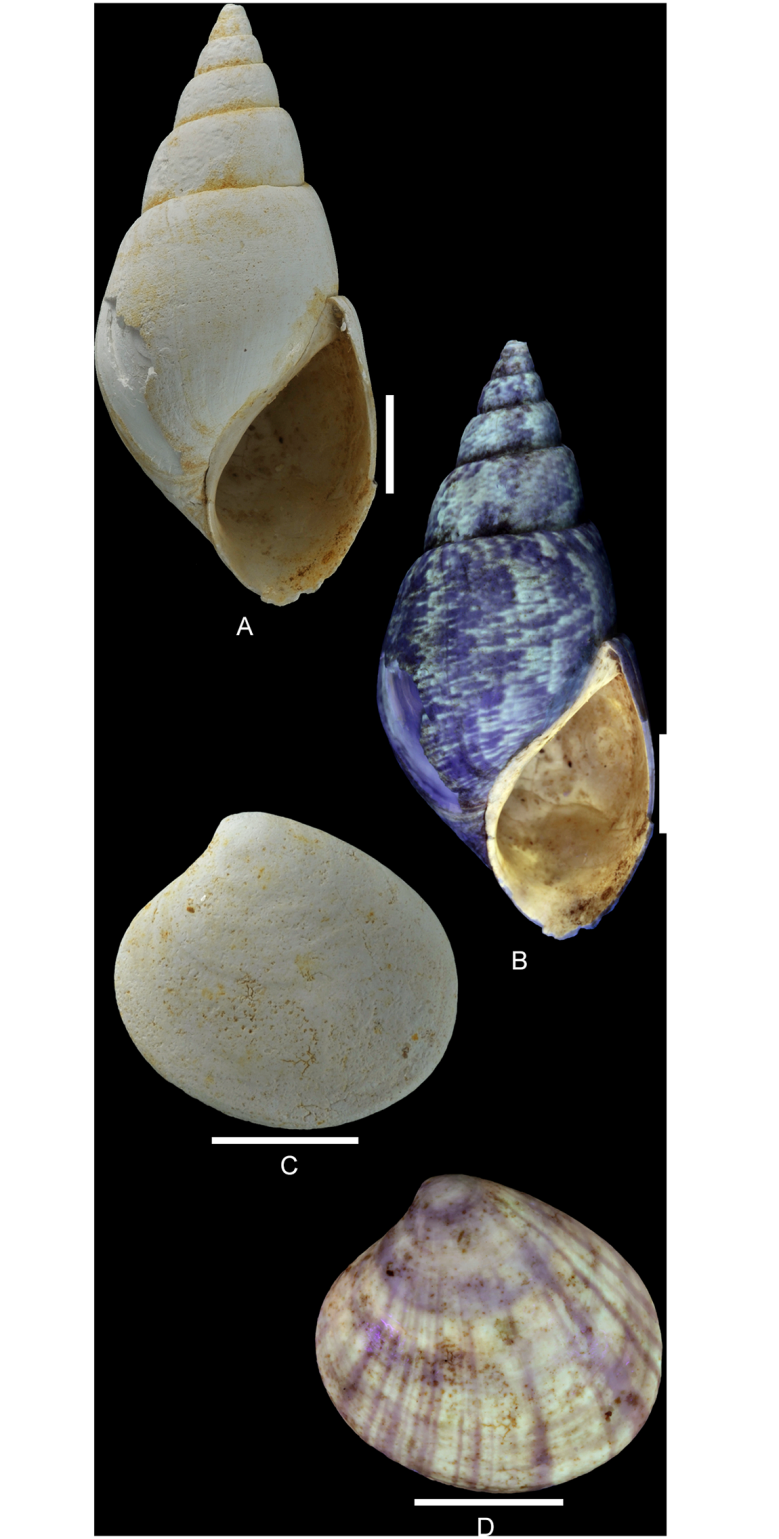
Residual colour patterns revealed in molluscs from the Oxfordian of Cordebugle (Calvados). (A, B) *Pseudomelania brasili* (Bigot, 1938), MNHN.F.A46213 (Bigot coll.). (A) in natural light. (B) under UV light. (C, D) *Neocrassina ovata* (Smith, 1817), MNHN.F.J10473 (Cossmann coll.). (C) in natural light. (D) under UV light. Seen under UV light, fluorescent areas correspond to areas with residual pigmentation while dark areas correspond to unpigmented regions. Scale bars: 10 mm.

Eudes-Deslongchamps (1843, p. 227, pl. 12, fig. 13) [39] described and figured a specimen of *Melania condensata* [*= Cloughtonia abbreviata* (Römer, 1836)] from Cordebugle showing narrow, axial stripes in natural light. This observation, together with the fact that aragonitic shells from Cordebugle are still preserved in their original mineralogy, prompted us to examine preliminarily other material from this locality under UV light, in a first attempt without immersion in a diluted sodium hypochlorite solution. Residual colour patterns were observed in several shells, but they were of low contrast, pale, and incomplete and needed to be enhanced. Subsequently, residual colour patterns were systematically revealed (Fig 2) by subjecting the shells to a procedure described in detail by Merle et al. (2008) [22]. First, the specimens were immersed in a concentrated sodium hypochlorite solution (solution of sodium hypochlorite at 9.6 percent chlorine) for 24 hours in order to enhance the residual colour. Subsequently, the shells were exposed to UV light emitting a wavelength of 3600 Å [40].

Due to the fragility of the mostly aragonitic shell material, many specimens have been glued previously. Due to these conservational issues as well as the scientific and historical value of these specimens, most historical types and figured specimens of the smallest species were not treated with the above described procedure.

### Descriptive terminology

Given the complexity and diversity of the observed shell colour patterns, the terminology necessary to describe them is often intricate. To facilitate proper understanding of the descriptions, detailed definitions of key terms are provided below. Most of these terms are newly introduced or were defined in a paper dealing with the residual colour patterns in ampullinid gastropods [20]. Keeping in mind that shell colour patterns are formed by the incorporation of pigments during shell growth [41], the following four criteria sufficiently characterize the revealed residual patterns: (1) the continuous or discontinuous incorporation of pigments along the growing edge (= over space); (2) the continuous or discontinuous incorporation of pigments during shell growth (= over ontogenetic time); (3) the spreading, shrinking or migration of the pigment incorporation areas along the growing edge over time; (4) the coincidence (positive relation) or distinctness (negative relation) of fluorescent elements of the colour pattern and of sculpture elements. The process of pigment incorporation is outlined in detail for each pattern revealed and the related terminology is provided.

**Fig 3:**
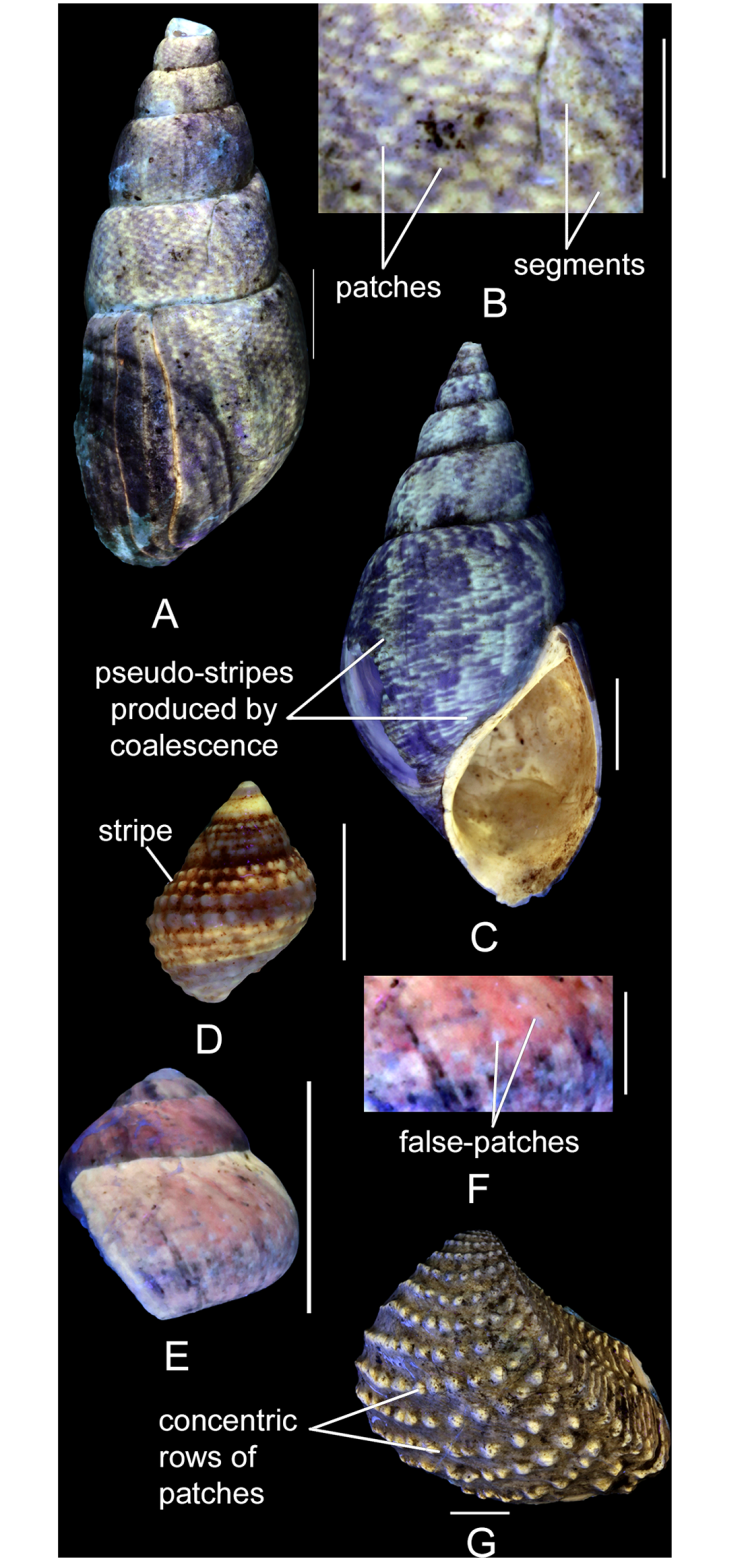
Descriptive terminology for the elements of colour patterns. (A, B) *Pseudomelania brasili* (Bigot, 1938), MNHN.F.J10471 (Cossmann coll.). (A) dorso-labral view. (B) detailed view of the colour pattern. (C) *P*. *brasili*, MNHN.F.A46213 (Bigot coll.), apertural view. (D) *Gerasimovcyclus* cf. *lorioli* (Schmidt, 1905), MNHN.F.J10402 (Cossmann coll.), dorsal view. (E, F) *Ataphrus (Ataphrus) marschmidti* Gründel and Kaim, 2006, MNHN.F.J11070 (Cossmann coll.). (E) labral view. (F) detailed view of the colour pattern. (G) *Myophorella nodulosa* (Lamarck, 1801), UPMC-138 (UPMC coll.), left valve view. Scale bars: 10 mm (A, C, E, G), 5 mm (B, D), 2 mm (F).

**Fig 4:**
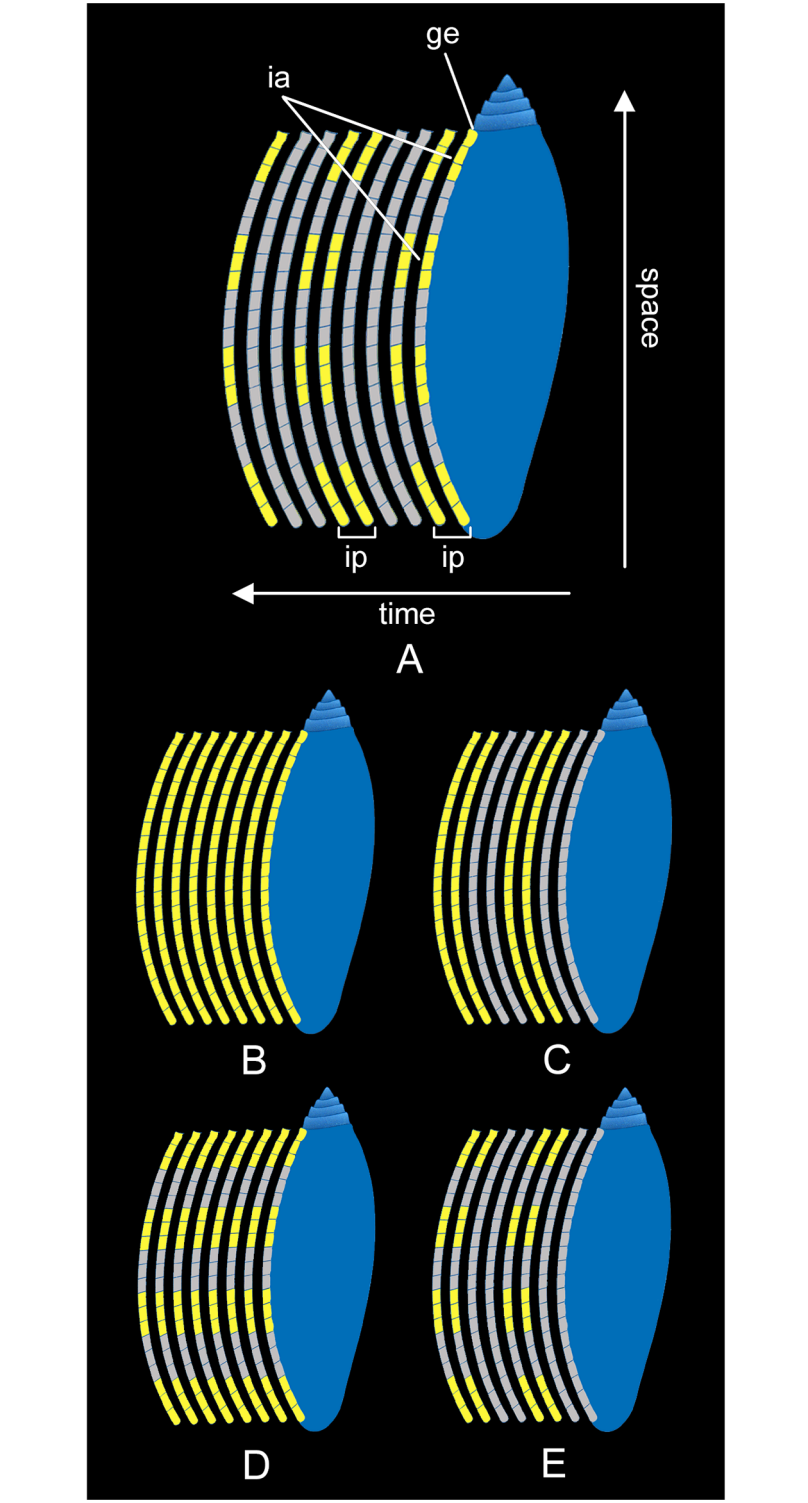
Descriptive terminology. (A) Descriptive terminology for the incorporation process of pigments. ia: incorporation area. ge: growing edge. ip: incorporation phase. (B-E) Examples of incorporation processes. (B) Continuous incorporation of pigments over space and time. (C) Continuous incorporation of pigments over space, along the entire growing edge, and discontinuous and recurrent over time. (D) Discontinuous incorporation of pigments over space, at several areas along the growing edge, and continuous over time. (E) Discontinuous incorporation of pigments over space, and discontinuous and recurrent over time.

- Patch: small fluorescent area sharply contrasting with the background (Fig 3A and 3B).
- False patch: small non fluorescent area contrasting with a fluorescent colouration covering a large part of the whorl or valve (Fig 3E and 3F).
- Stripe (axial/spiral or radial/commarginal): elongated and fluorescent element, continuous on the whole whorl or valve (Fig 3D).
- Segment (axial/spiral or radial/commarginal): elongated and fluorescent element, discontinuous on the whorl or valve and frequently resulting from the coalescence of patches (Fig 3A and 3B).
- Pseudo-stripe (axial/spiral or radial/concentric): elongated fluorescent element, continuous on the whole whorl or valve resulting from the coalescence of patches or segments (Fig 3C). Often the coalescence of patches or segments does not start immediately but sets on later in ontogeny.
- Row: series of aligned fluorescent patches or segments (Fig 3G).
- Coalescence: partial or complete merging of adjacent fluorescent elements, due to the increase in their size or density of distribution. This may include the formation of a larger element of spatial continuity from patches (Fig 3A), segments, stripes or rows.

- Incorporation area: part of the growing edge at which the animal incorporates the pigments involved in the colour pattern formation during the shell growth (Fig 4A, ia).
- Incorporation phase: period of relative time during which pigments are incorporated into the shell in formation (Fig 4A, ip)

## Results

### Distribution of residual colour patterns among the taxa

Among the 46 species studied, 14 out of 28 species of gastropods and 11 out of 18 species of bivalves display a residual colour pattern under UV light (S1 Table). Representatives of the following 10 gastropod and 8 bivalve superfamilies were studied: (1) Gastropoda: Pleurotomarioidea (Pleurotomariidae), Trochoidea (Proconulidae, Ataphridae), Seguenzioidea (Eucyclidae), Neritoidea (Neritidae), Pseudomelanioidea (Pseudomelaniidae), Campaniloidea (Ampullinidae), Cerithioidea (Procerithiidae), Rissooidea (Rissoidae), Nerineoidea (Ceritellidae), Acteonoidea (Acteonellidae, Bullinidae), (2) Bivalvia: Mytiloidea (Mytilidae), Arcoidea (Cucullaeidae), Ostreoidea (Flemingostreidae), Trigonioidea (Trigoniidae), Myophorelloidea (Myophorellidae), Crassatelloidea (Astartidae), Lucinoidea (Lucinidae), Sphaerioidea (Neomiodontidae). Positive results were obtained for most of these groups. The Pleurotomarioidea, Neritoidea, Rissooidea, and Acteonoidea among the gastropods, and the Arcoidea, Ostreoidea and Trigonioidea among the bivalves did not exhibit any fluorescence. Within the taxa showing colour patterns under UV light, positive results were achieved for 49.37% of the specimens in gastropods and 31% of the specimens in bivalves. However, the preservation rate of residual colour patterns is variable among different taxa. For example, 66.67% of all individuals in the Pseudomelaniidae show residual colour patterns, while only 38.71% of the specimens in the Ampullinidae reveal residual colours.

Negative results, i.e. the lack of residual colour patterns may either be caused by (1) diagenetic alteration destroying previously existing patterns, or by (2) an original lack of pigmentation of the shells. An interesting case illustrating the particular value of negative results is the Acteonoidea. This superfamily comprises three extant families, the Acteonidae, Aplustridae and Bullinidae. Rates of species displaying colour patterns are approximately 40% for the 54 actaeonid species, 100% for the seven aplustrid species and 100% for the ten bullinid species [42]. In the Paleogene of the Paris Basin, none of the three Lutetian acteonid species tested (from 35 specimens altogether) displayed a residual colour pattern. From the Oxfordian of Cordebugle, 70 specimens belonging to three species of the Acteonellidae and one species of the Bullinidae were examined and did not show fluorescence at all. Since colour patterns are not evenly distributed among Recent taxa and are lacking in Paleogene taxa, the lack of fluorescence in the Oxfordian taxa probably results from a true lack of shell colouration. The occurrence and diversification of shell colour patterns in stratigraphically younger (post-Paleogene) representatives may constitute an evolutionary trend in the Actaeonoidea.

### Systematic survey of colour patterns

Class Gastropoda Cuvier, 1797

Clade Vetigastropoda Salvini-Plawen, 1980

Family Ataphridae Cossmann, 1915

Genus *Ataphrus* Gabb, 1869

Subgenus *Ataphrus* Gabb, 1869

*Ataphrus (Ataphrus) marschmidti* Gründel and Kaim, 2006 [43]

(Fig 5A–5D)

**Fig 5:**
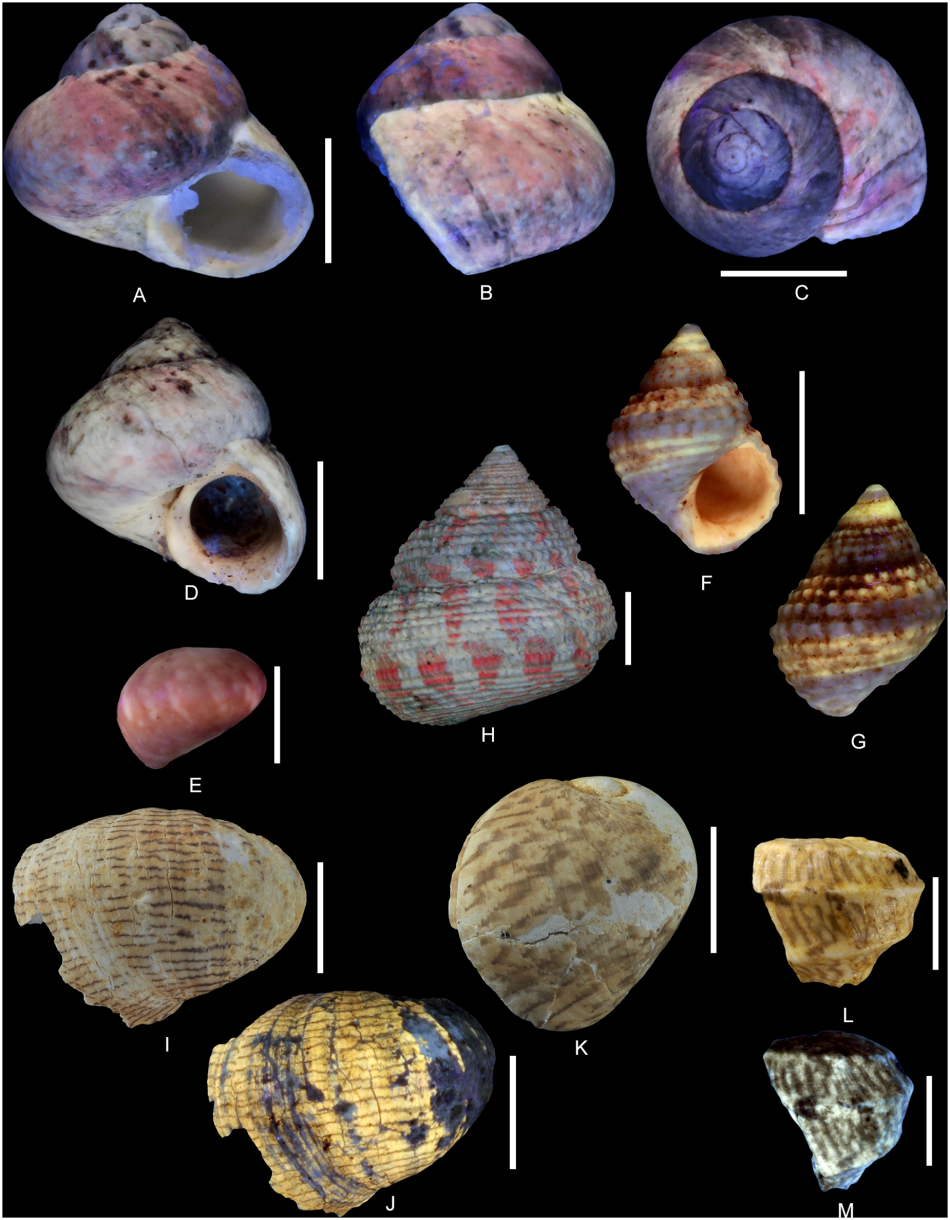
Residual colour patterns in Vetigastropoda and Neritimorpha. (A-G, I-K) Gastropods from the Oxfordian of Cordebugle (Calvados). (A, B) *Ataphrus (Ataphrus) marschmidti* Gründel and Kaim, 2006, MNHN.F.J11070 (Cossmann coll.). (A) apertural view. (B) labral view. (C) *A*. *(A*.*) marschmidti*, MNHN.F.J11074 (Cossmann coll.), apical view. (D) *A*. *(A*.*) marschmidti*, UPMC-136 (Le Marchand coll.), apertural view. (E) *A*. *(Endianaulax) sarahae* (Chavan, 1954), UPMC-137 (Le Marchand coll.), dorsal view. (F, G) *Gerasimovcyclus* cf. *lorioli* (Schmidt, 1905), MNHN.F.J10402 (Cossmann coll.). (F) apertural view. (G) dorsal view. (H) *Calliomphalus (Calliomphalus) squamulosus* (Lamarck, 1804) from the Lutetian of the Paris Basin showing red fluorescence, MNHN.F.A24999 (Pacaud coll.), dorsal view. (I, J) *Neridomus ovula* (Buvignier, 1843), MNHN.F.A32286 (de Morgan coll.), dorso-labral view. (I) in natural light. (J) under UV light. (K), *Neridomus* sp., MNHN.F.A32268 (de Morgan coll.), dorso-labral view. (L, M) *Pseudodostia pentastoma* (Deshayes, 1864) from the Lutetian of the Paris Basin, MNHN.F.A31564 (Faullummel coll.), labral view. (L) in natural light. (M) under UV light. Scale bars: 5 mm (A, B, C, D, F, G), 2 mm (E, L, M), 10 mm (H, I, J, K).

Examined material: 2 spm (Cossmann coll.: MNHN.F.J11074 and MNHN.F.J11070) and 1 spm (Le Marchand coll.: UPMC-136).

The residual colour pattern consists of two components: (1) a slightly paler, red, fluorescent colour and (2) a dark, non-fluorescent background. The red colouration covers almost the entire surface of the whorls whereas the dark background is only apparent on numerous small subcircular false patches (Fig 5A and 5B). These are irregularly distributed and sometimes contiguous. This pattern results from discontinuous to continuous incorporation of pigments over space and continuous incorporation over time. During shell growth, short, localized incorporation pauses occur at parts of the growing edge (see S1 Fig).

Comments: The description of the colour pattern is based on the specimen illustrated in Fig 5A and 5B. The other figured specimens of *A*. *(A*.*) marschmidti* are less well preserved and display only incomplete colour patterns consisting of rather irregular red fluorescent elements (Fig 5C and 5D). Consequently, they do not provide sufficient information with regard to the intraspecific variability of the colour pattern. However, all specimens exhibit red fluorescence. In Cenozoic fossil shells, this red tint typically occurs in Vetigastropoda [19].

Subgenus *Endianaulax* Chavan, 1954

*Ataphrus (Endianaulax) sarahae* (Chavan, 1954) [30]

(Fig 5E)

Examined material: 1 spm (Le Marchand coll.: UPMC-137).

Taxonomic note: Gründel (2008, p. 180–181) [44] considers *Teinostomopsis* Chavan, 1954 as a junior synonym of *Endianaulax*, which he assigns as a subgenus to *Ataphrus*.

The residual colour pattern consists of two components: (1) a red fluorescent colouration that covers almost the entire last whorl and (2) a slightly paler, non-fluorescent background. The background is visible only in a few irregular elongated, axial, roughly prosocline false patches in the median part of the last whorl (Fig 5E). This pattern results from discontinuous to continuous incorporation of pigments over space and continuous incorporation over time. Several incorporation pauses occur in the median part of the growing edge (see S2 Fig).

Comments: As mentioned above, the red fluorescence seen in the shell of *A*. *(E*.*) sarahae* under UV light typically occurs in the Cenozoic fossil Vetigastropoda (Fig 5) [19].

Family Eucyclidae Koken, 1896

Genus *Gerasimovcyclus* Gründel, 2005

*Gerasimovcyclus* cf. *lorioli* (Schmidt, 1905) [45]

(Fig 5F–5G)

Examined material: 2 spm (Cossmann coll.: MNHN.F.J10462 and MNHN.F.J10402) and 4 spm (Le Marchand coll.: UPMC-129 and UPMC-199).

Taxonomic note: The shell shape and the ornamentation of the French specimens are similar to those of the Polish specimens illustrated by Gründel and Kaim (2006) [43]. However, the French specimens differ by a bent columella and by the microstructure of the apertural margin with only two layers: (1) an outer probably primastic layer and (2) an inner nacreous layer (see S3 Fig).

The residual colour pattern consists of two components: (1) five pale yellow, fluorescent spiral stripes and (2) a dark, non-fluorescent background. The stripes are narrow, straight, parallel and positioned on spiral cords. Two stripes are situated just below the suture while three stripes occur on the median part of the whorl (Fig 5F and 5G). This pattern results from discontinuous incorporation of pigments over space and continuous incorporation over time. The location and size of the incorporation areas corresponds to elements of the spiral sculpture (see S4 Fig).

Comments: Among the three studied species of Vetigastropoda from Cordebugle that show fluorescent colour patterns under UV light, the shell of *Gerasimovcyclus* cf. *lorioli* is the only one that emits yellow rather than red fluorescence. Although the broken apertural margin of two of the examined specimens reveals a nacreous inner layer that is characteristic of the vetigastropods (see S3 Fig), yellow fluorescence is typically observed in all non-vetigastropods. This yellow fluorescence is unique also with regard to the entire set of 31 Mesozoic and Cenozoic fossil vetigastropod species examined so far [19, 22, 46–48]. The Lutetian eucyclid *Calliomphalus (Calliomphalus) squamulosus* (Lamarck, 1804) is figured as an example (Fig 5H, [22]).

Two hypotheses may be advanced to explain this exception.

(1) The single specimen showing yellow fluorescence underwent a peculiar diagenetic process leading to an uncommon alteration of the pigments—As this specimen is very well preserved and all the Jurassic species studied herein come from the same locality, we regard it unlikely that this single shell underwent a unique process of colour deterioration with regard to the other vetigastropod shells.

(2) Considering that the wavelength of the emitted fluorescence is potentially related to the chemical composition of the pigments, the different colours of fluorescence may relate to different pigments. Accordingly, some taxa within the Vetigastropoda may incorporate other pigments than the majority of the group.—Given the diversity of shell pigments in molluscs [49–50], individual species or genera of Vetigastropoda may well produce different pigments causing different colours of fluorescence under UV light.

Clade Neritimorpha Golikov and Starobogatov, 1975

Family Neritidae Rafinesque, 1815

Genus *Neridomus* Morris and Lycett, 1851

*Neridomus ovula* (Buvignier, 1843) [51]

(Fig 5I and 5J)

Examined material: 10 spm (de Morgan coll.: MNHN.F. B45749 and A32286).

Taxonomic note: Gründel and Kaim (2006) [43] raised *Neridomus* to full generic status.

In natural light, the residual colour pattern consists of two components: (1) more than twenty thin dark, spiral stripes and (2) a paler background (Fig 5I). These stripes are faintly undulating, roughly parallel and of similar thickness. They are close to each other and regularly distributed across the entire whorl. This pattern is produced by discontinuous incorporation of pigments over space, on numerous areas along the growing edge, and continuous incorporation over time (see S5 Fig).

Comments: Under UV light, the figured specimen and four other specimens out of the nine examined shells show a pale yellow fluorescence between the dark spiral stripes (Fig 5J). This yellow fluorescence could be caused by the degradation of shell pigments as observed in numerous fossil Caenogastropoda (see [19] and below) or by a peculiar diagenetic process undergone by the fossil shell and involving no pigment. In the first case, this suggests the presence of an additional level of colouration supplementing the dark spiral stripes, which would also imply more complex mechanisms of pigment incorporation. To date, yellow fluorescence under UV light has never been observed on Cenozoic neritids (e.g., Fig 5L and 5M).

*Neridomus* sp.

(Fig 5K)

Examined material: 1 spm (de Morgan coll.: MNHN.F.A32268).

In natural light, the residual colour pattern consists of two components: (1) dark zigzag stripes and (2) a paler background. The stripes are largely parallel and opisthocline (Fig 5K). This pattern results from a discontinuous incorporation of pigments over space and continuous incorporation over time. In a first step, the incorporation starts in several, relatively distant areas of the adapical part of the growing edge. Shortly after their onset, these areas split into two, which spread in adapical and abapical directions, respectively, forming upward and downward directed, oblique subelements. In a second step, new incorporation areas turn on in abapical position relative to the previous ones, and split in a similar manner; and so on. Gradually, this leads to the fusion of the downsloping area with the adjacent upsloping area. After these areas fuse, they do not pursue the incorporation. With time, this process leads to the formation of zigzags (see S6 Fig).

Comments: Although the colour pattern of *Neridomus* sp. (Fig 5K) is clearly distinct from that of *Neridomus ovula* (Fig 5I), these species cannot be distinguished based on shell shape. Taking the strong intraspecific variability of colour patterns in numerous neritid species into account, we refrain from describing a new species based on the single specimen of *Neridomus* sp.

Comments on the family Neritidae: In the fossil record, the neritids are the most common gastropods to preserve remnants of the colour patterns visible in natural light. This is also the case for the Oxfordian specimens from Cordebugle (Fig 5I and 5K). Since the unusual preservation of neritid colour patterns seems occur rather independant of the age of the fossils or sedimentology and taphonomy, we consider it as relying on intrinsic factors. It is likely related to the chemical composition of the pigments involved. In order to validate this hypothesis, the composition of shell pigments would need to be established.

Clade Caenogastropoda Cox, 1960

Family Pseudomelaniidae Hoernes, 1884

Genus *Pseudomelania* Pictet and Campiche, 1862

*Pseudomelania brasili* (Bigot, 1938) [25]

(Figs 6 and 7)

**Fig 6:**
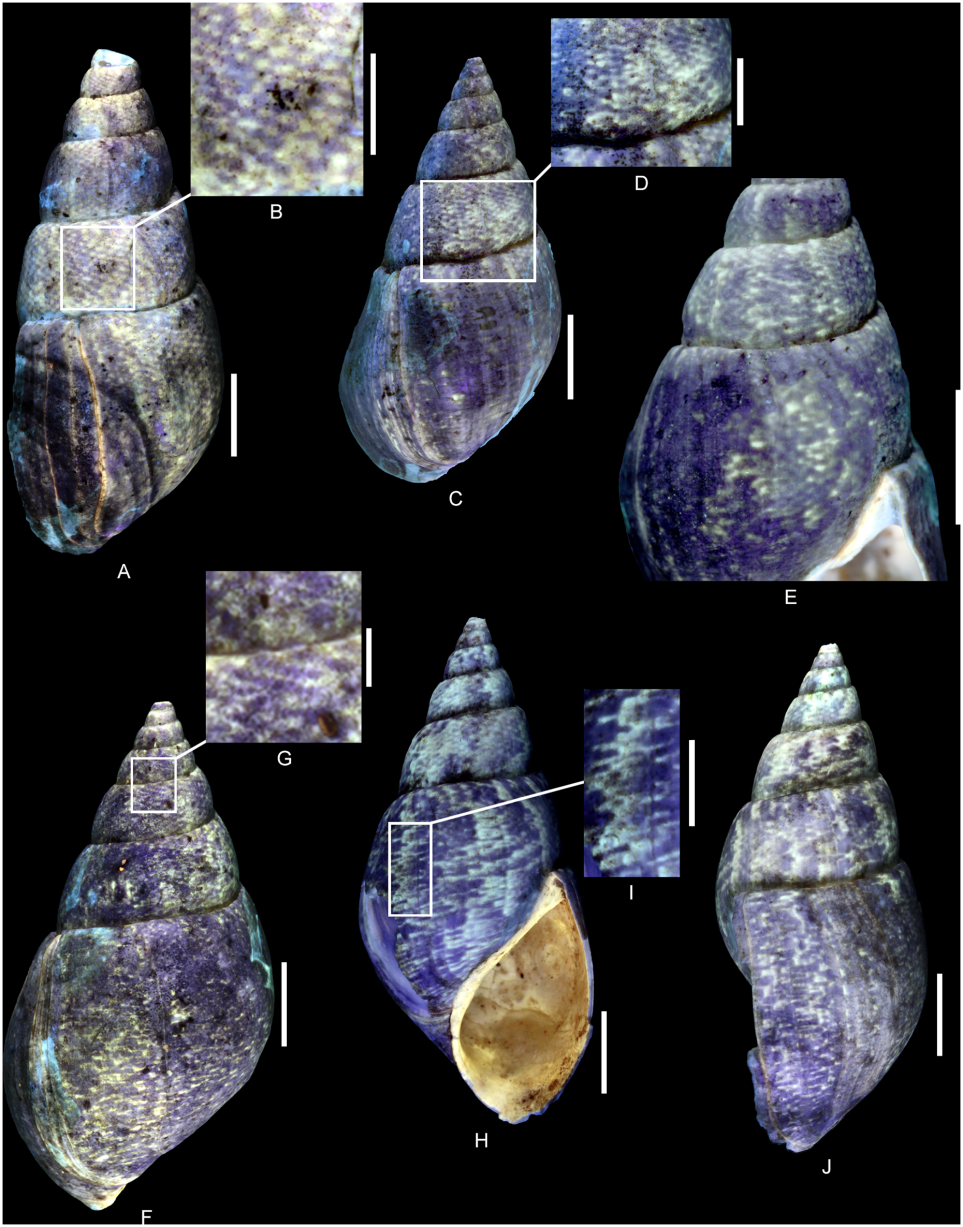
Residual colour pattern of *Pseudomelania brasili* (Bigot, 1938) from the Oxfordian of Cordebugle (Calvados). (A, B) MNHN.F.J10471 (Cossmann coll.). (A) dorso-labral view. (B) detailed view of the colour pattern. (C-E) MNHN.F.J10208 (Cossmann coll.). (C) dorsal view. (D) detailed view of the colour pattern. (E) detailed view of the colour pattern in apertural view. (F, G) MNHN.F.J10468 (Cossmann coll.). (F) dorsal view. (G) detailed view of the colour pattern. (H-J) MNHN.F.A46213 (Bigot coll.). (H) apertural view. (I) detailed view of the colour pattern. (J) labral view. Scale bars: 10 mm (A, C, E, F, H, J), 5 mm (B, D, I), 2 mm (G).

**Fig 7:**
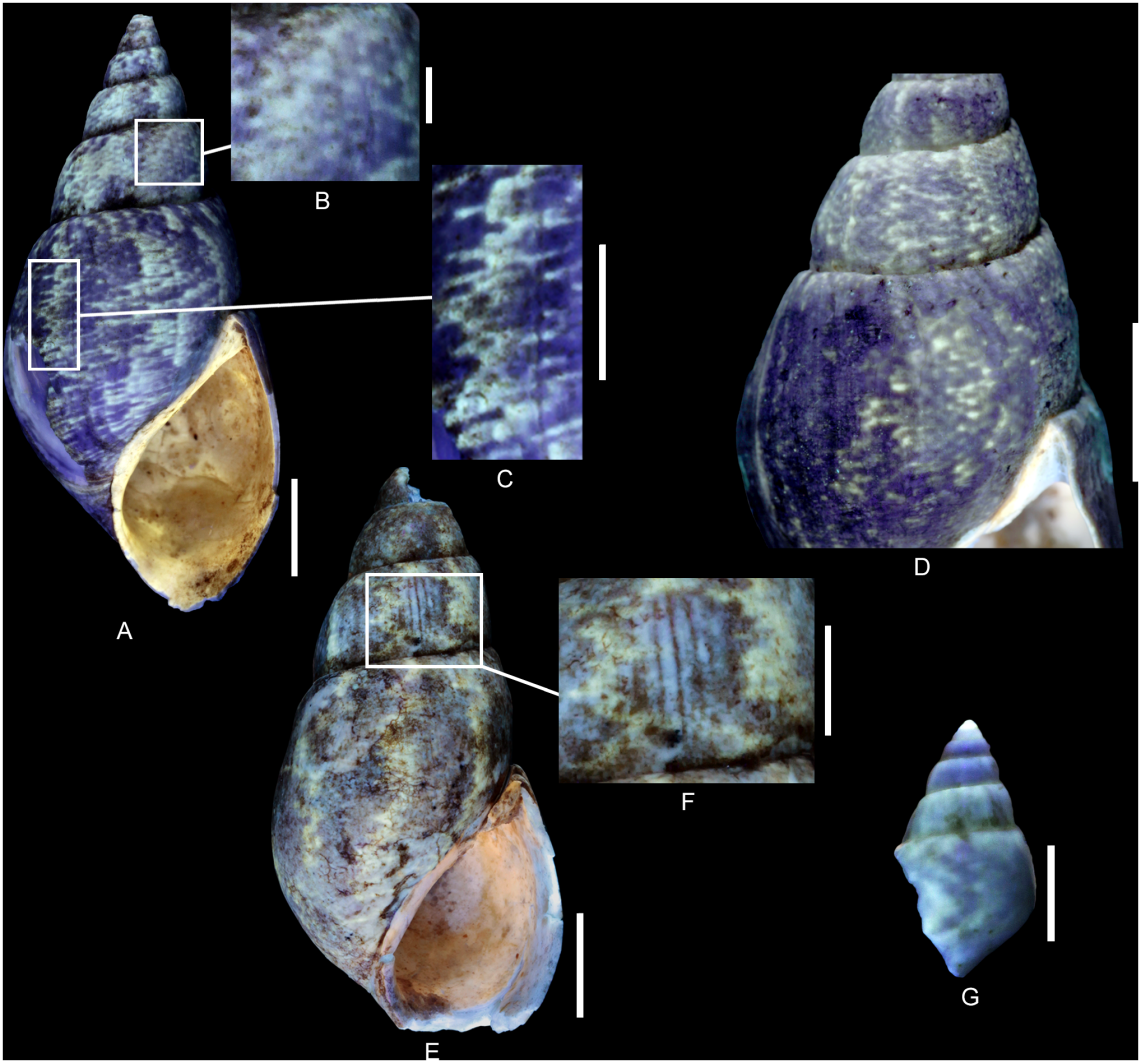
Colour pattern variants of *Pseudomelania brasili* (Bigot, 1938) from the Oxfordian of Cordebugle (Calvados). (A-C) MNHN.F.A46213 (Bigot coll.). (A) apertural view. (B, C) detailed views of the colour pattern. (D) MNHN.F.J10208 (Cossmann coll.), detailed view of the colour pattern in apertural view. (E, F) UPMC-133 (Curet coll.). (E) apertural view. (F) detailed view of the colour pattern. (G) UPMC-135 (UPMC coll.), juvenile individual in labral view. Scale bars: 10 mm (A, D, E), 5 mm (C, F), 2 mm (B, G).

Examined material: 3 spm (Cossmann coll.: MNHN.F.J10467, J10468 and J10471), 6 spm (Bigot coll.: MNHN.F. A32278, A46212 and A46213), 6 spm (Le Marchand coll.: UPMC, including one figured specimen UPMC-135) and 1 spm (Curet coll.: UPMC-133).

The residual colour pattern consists of two components: (1) numerous, small, pale yellow, fluorescent patches and (2) a dark, non-fluorescent background (Fig 6A–6D). The patches are subcircular to slightly spirally elongate in outline, homogeneous in size and arranged in staggered rows. Meinhardt (1998) [41] coined the term “meshwork” for this kind of pattern. It is produced by discontinuous incorporation of pigments, both over space and time. Small incorporation areas are regularly distributed along the growing edge and get slightly shifted with each incorporation phase (see S7 Fig).

Variability: *P*. *brasili* displays a strong intraspecific variability with regard to colour pattern. Frequently patches coalesce to form short, oblique segments (Fig 6B), larger patches (Fig 6D), chevrons (Fig 6G), or zigzag axial pseudo-stripes (Fig 6H–6J). These axial pseudo-stripes show numerous small zigzags of various amplitudes. Despite this large variability (Figs 6 and 7), the transition from the common staggered rows of patches to zigzag axial pseudo-stripes is gradual (Fig 7). Some specimens may even show both extremes (Fig 7A–7C). With regard to the mechanisms of pigment incorporation, this variability results from the merger of contiguous incorporation areas along the growing edge, and from continuity of incorporation between several phases.

Comments: The same colour pattern consisting of small patches that coalesce to form chevrons or axial zigzag pseudo-stripes has been observed in a juvenile specimen (Fig 7G). This suggests that the secretion and incorporation of pigments starts early in the ontogeny of *P*. *brasili*.

*Pseudomelania cornelia* (d’Orbigny, 1851) [32]

(Fig 8A–8F)

**Fig 8:**
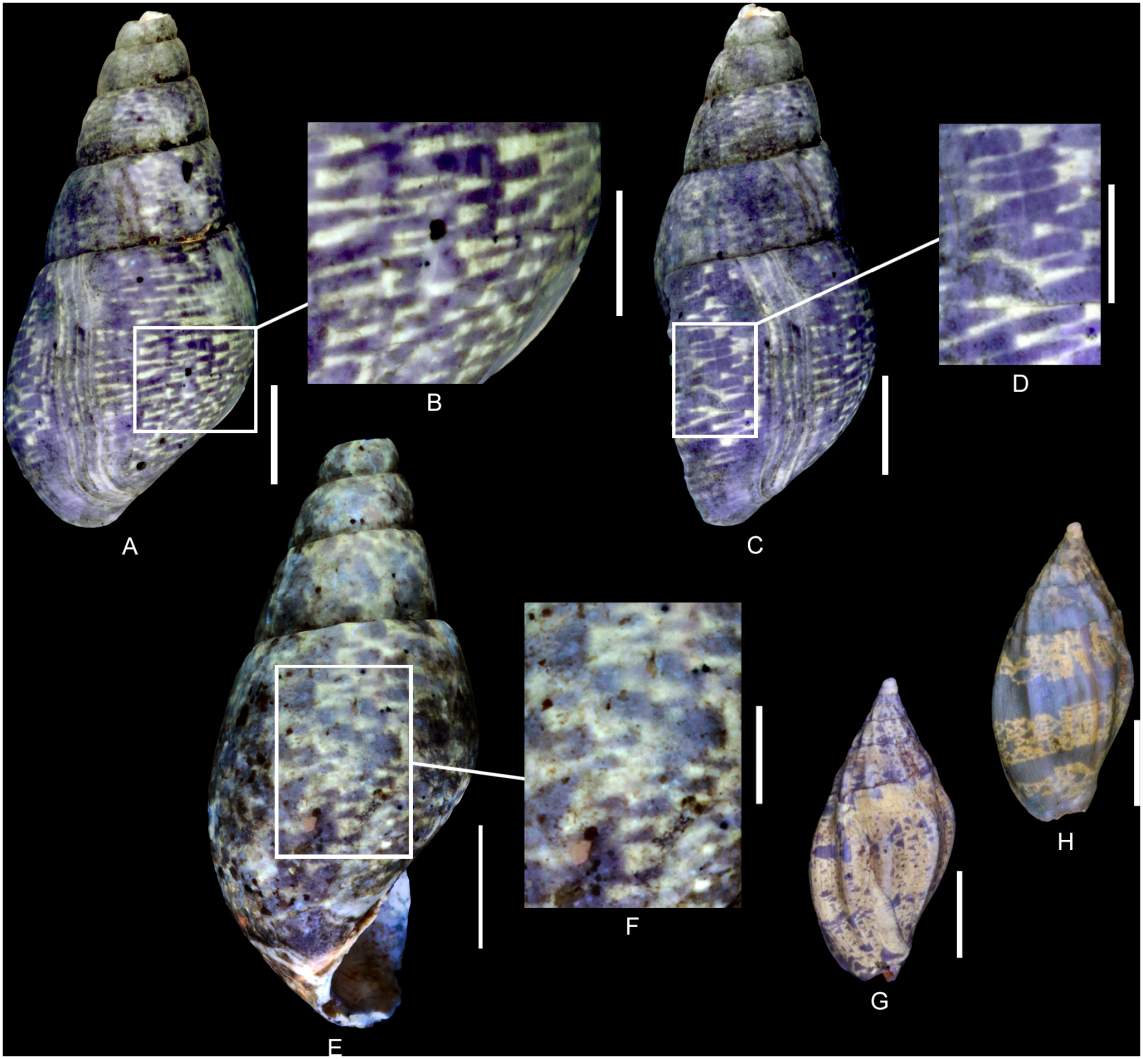
Residual colour pattern of *Pseudomelania cornelia* (d’Orbigny, 1851) from the Oxfordian of Cordebugle (Calvados). (A-F) *Pseudomelania cornelia*. (A-D) MNHN.F.A45819 (Bigot coll.). (A) dorsal view. (B) detailed view of the colour pattern. (C) labral view. (D) detailed view of the colour pattern. (E, F) UPMC-134 (Curet coll.). (E) ablabral view. (F) detailed view of the colour pattern. (G, H) *Mitreola maxwelli* (Le Renard, 1994) from the Lutetian of the Paris basin, a volutid showing similar patches. (G), MNHN.F.B64860 (MNHN coll.), dorsal view. (H) MNHN.F.A25041 (Faullummel coll.), dorsal view. Scale bars: 10 mm (A, C, E), 5 mm (B, D, F-H).

Examined material: 1 spm (Bigot coll.: MNHN.F.A45819) and 1 spm (Curet coll.: UPMC-134).

The residual colour pattern consists of two components: (1) numerous, yellow, fluorescent and spirally elongated patches and (2) a dark, non-fluorescent background (Fig 8A–8F). These spirally elongated patches appear largely coalescent and their shape is therefore difficult to characterize (probably subtriangular, Fig 8B and 8F). The coalescence produces a network that subdivides the background into small, dark, subtriangular, false patches (Fig 8B, 8D and 8F). These numerous false patches are irregularly distributed and frequently contiguous or continuous. Their apex is directed towards the growing edge. This pattern results from discontinuous incorporation of pigments over space and continuous incorporation over time. From time to time, there are some incorporation stops along the growing edge that are shorter or longer during the shell growth (see S8 Fig).

Comments: A similar pattern of fluorescent, superimposed colouration, where the dark background shines through only in the form of dark triangles that point towards the aperture, is known from *Mitreola maxwelli* (Le Renard, 1994) from the Lutetian of the Paris Basin (Fig 8G and 8H).

*Pseudomelania collisa* de Loriol, 1874 [52]

(Fig 9)

**Fig 9:**
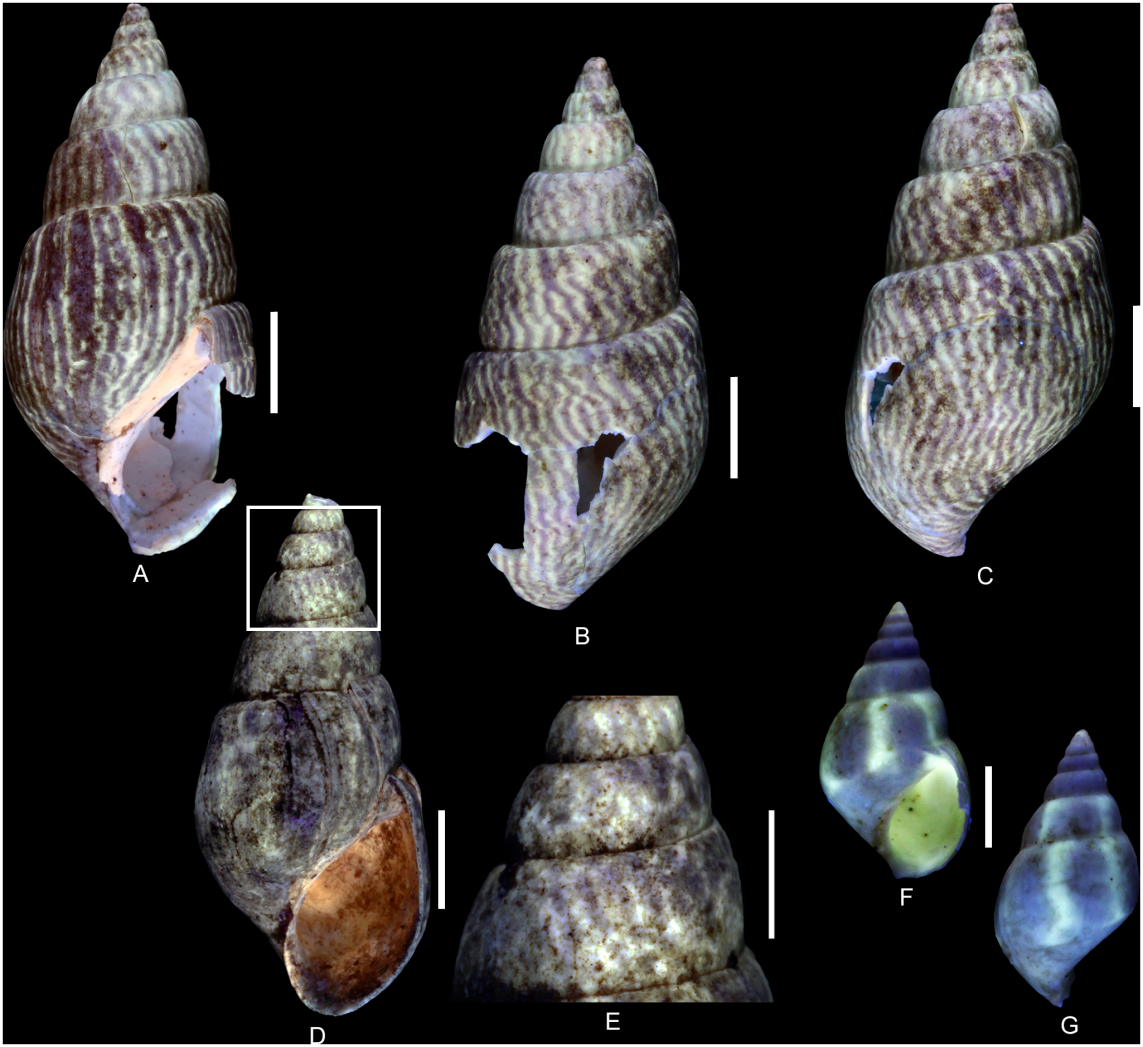
Residual colour pattern of *Pseudomelania collisa* (de Loriol, 1874) from the Oxfordian of Cordebugle (Calvados). (A-C) UPMC-131 (Le Marchand coll.). (A) apertural view. (B) labro-dorsal view. (C) dorsal view. (D, E) UPMC-130 (Le Marchand coll.). (D) apertural view. (E) detailed view of the colour pattern. (F, G) UPMC-132 (UPMC coll.). (F) juvenile in apertural view. (G) juvenile in ablabral view. Scale bars: 10 mm (A-D), 5 mm (E), 2 mm (F, G).

Examined material: 2 spm (Le Marchand coll.: UPMC-130 and 131) and 4 spm (UPMC coll., including one figured specimen UPMC-132).

The residual colour pattern consists of two components: (1) axial, yellow, fluorescent stripes and (2) a dark, non-fluorescent background (Fig 9A–9C). These stripes are approximately parallel to the growing edge and their inclination may vary from slightly sinuous to sigmoidal. This pattern results from roughly continuous incorporation of pigments over space and discontinuous, recurrent incorporation over time (see S9 Fig).

Comments: The same colour pattern already occurs in juvenile specimens (Fig 9F and 9G), suggesting that the secretion and incorporation of pigment in *P*. *collisa* starts early in ontogeny.

Comments on the genus *Pseudomelania*: According to the original description of the genus by Pictet and Campiche (1862) [53], *Pseudomelania* lacks an umbilicus. Since some of the studied specimens from Cordebugle show a small umbilicus, the generic diagnosis needs to be emended.

Superfamily Campaniloidea Douvillé, 1904

Family Ampullinidae Cossmann, 1919

Genus *Ampullina* Bowdich, 1822

*Ampullina clio* (d’Orbigny, 1850) [54]

(Fig 10A–10L)

**Fig 10:**
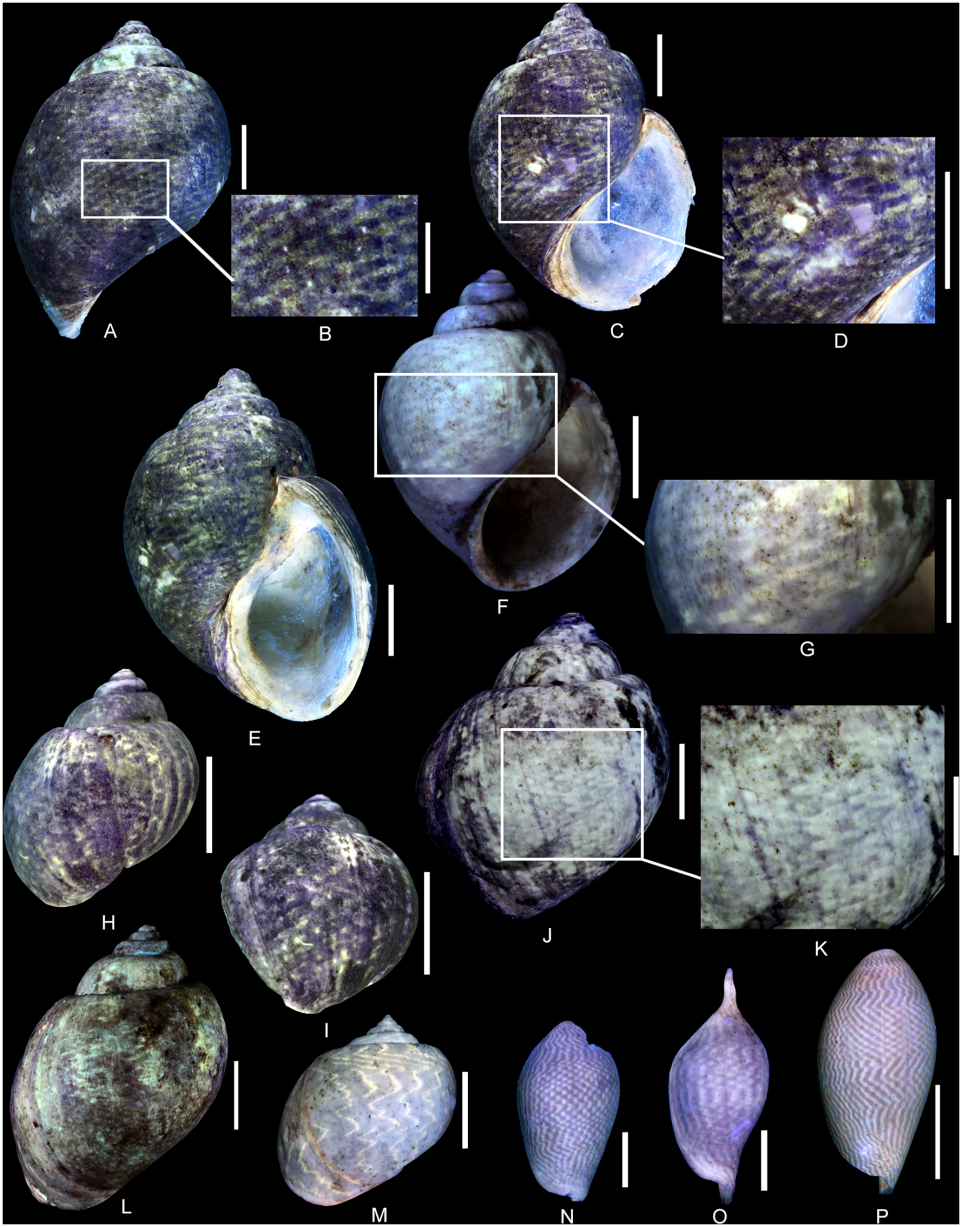
Residual colour pattern of *Ampullina clio* (d’Orbigny, 1850) from the Oxfordian of Cordebugle (Calvados). (A-L) *Ampullina clio*. (A-E) MNHN.F.J10210 (Cossmann coll.). (A) ablabral view. (B) detailed view of the colour pattern. (C) apertural view. (D) detailed view of the colour pattern. (E) aperturo-labral view. (F, G) MNHN.F.J10426 (Cossmann coll.). (F) apertural view. (G) detailed view of the colour pattern. (H, I) MNHN.F.J10425 (Cossmann coll.). (H) dorsal view. (I) labro-dorsal view. (J, K) MNHN.F.A46211 (Bigot coll.). (J) dorsal view. (K) detailed view of the colour pattern. (L) MNHN.F.A30347 (de Morgan coll.), dorsal view. (M-P) Gastropods from the Eocene of the Paris basin showing a similar meshwork pattern. (M) *Globularia (Globularia) patuloides* (Cossmann and Pissarro, 1902), MNHN.F.A31162 (Faullummel coll., Bartonian), dorsal view. (N) *Diameza (Miniseraphs) eratoides* (Cossmann, 1889), MNHN.F.A28934 (Pacaud coll., Lutetian), dorsal view. (O) *D*. *(Diameza) fragilis* (Defrance, 1825), MNHN.F.A28939 (Pacaud coll., Lutetian), dorsal view. (P) *D*. *(Miniseraphs) isabella* (Bernay *in* Deshayes, 1865), MNHN.F.A28936 (Pacaud coll., Lutetian), dorsal view. Scale bars: 10 mm (A, C, D, E, H-J, M), 5 mm (B, F, G, K, O), 2 mm (M, N).

Examined material: 3 spm (Cossmann coll.: MNHN.F.J10210, J10425 and J10426), 5 spm (Bigot coll.: MNHN.F. B45795 and A46211), 4 spm (de Morgan coll.: MNHN.F. B45796 and A30347), 6 spm (Le Marchand coll.: UPMC) and 2 spm (UPMC coll).

Taxonomic note: According to Fischer and Weber (1997, p 74–76, 80) [55], this species belongs to the genus *Globularia* Swainson, 1840. The studied specimens, however, do not show the diagnostic features stated in the original description of this genus (wide aperture with very prosocline outer lip, low spire, expanded sheath delineated by a rim). The shells from Cordebugle rather display the characteristic shell shape of *Ampullina* Bowdich, 1822 and are therefore referred to this genus herein.

The residual colour pattern consists of two components: (1) numerous small, pale yellow-white and fluorescent patches and (2) a dark, non-fluorescent background (Fig 10A–10B). The patches are frequently spirally elongated. Generally, the patches are arranged in staggered rows. This pattern, called “meshwork” following Meinhardt (1998) [41], is produced by a discontinuous incorporation of pigments over space and time. Numerous small incorporation areas are regularly distributed along the growing edge and their positions change according to the incorporation phases (see S10 Fig).

Variability: *A*. *clio* displays a strong intraspecific variability with regard to colour pattern. The patches frequently coalesce forming short oblique segments, chevrons (Fig 10C–10E) and axial zigzagging segments and pseudo-stripes (Fig 10H–10L). These segments and pseudo-stripes show numerous zigzags of various amplitudes. Transition from non-coalescent to coalescent morphology is gradual and most specimens show two variants (Fig 10). With regard to the incorporation mechanisms of the pigments, these variations result from the merging of incorporation areas along the growing edge and with a relative continuity between incorporation phases.

Comments: This colour pattern is observed for the first time in the family Ampullinidae. None of the previously examined Cenozoic ampullinid species shows fluorescent patches arranged in staggered rows, or meshwork pattern [20]. Cenozoic Ampullinidae may also show fluorescent axial zigzagging elements (Fig 10M, [20]) which are, however, not variants of a meshwork pattern and the incorporation mechanisms is obviously different. With regard to the incorporation mechanisms, the colour pattern of *A*. *clio* is indeed more similar to those of the pseudomelaniid species *Pseudomelania brasili* Bigot, 1938 or of the Cenozoic seraphsid genus *Diameza* Deshayes, 1865 (Fig 10N–10P, [18]) than to that of Cenozoic ampullinids.

Genus *Cloughtonia* Huddleston, 1882

*Cloughtonia abbreviata* (Römer, 1836) [56]

(Fig 11A–11G)

**Fig 11:**
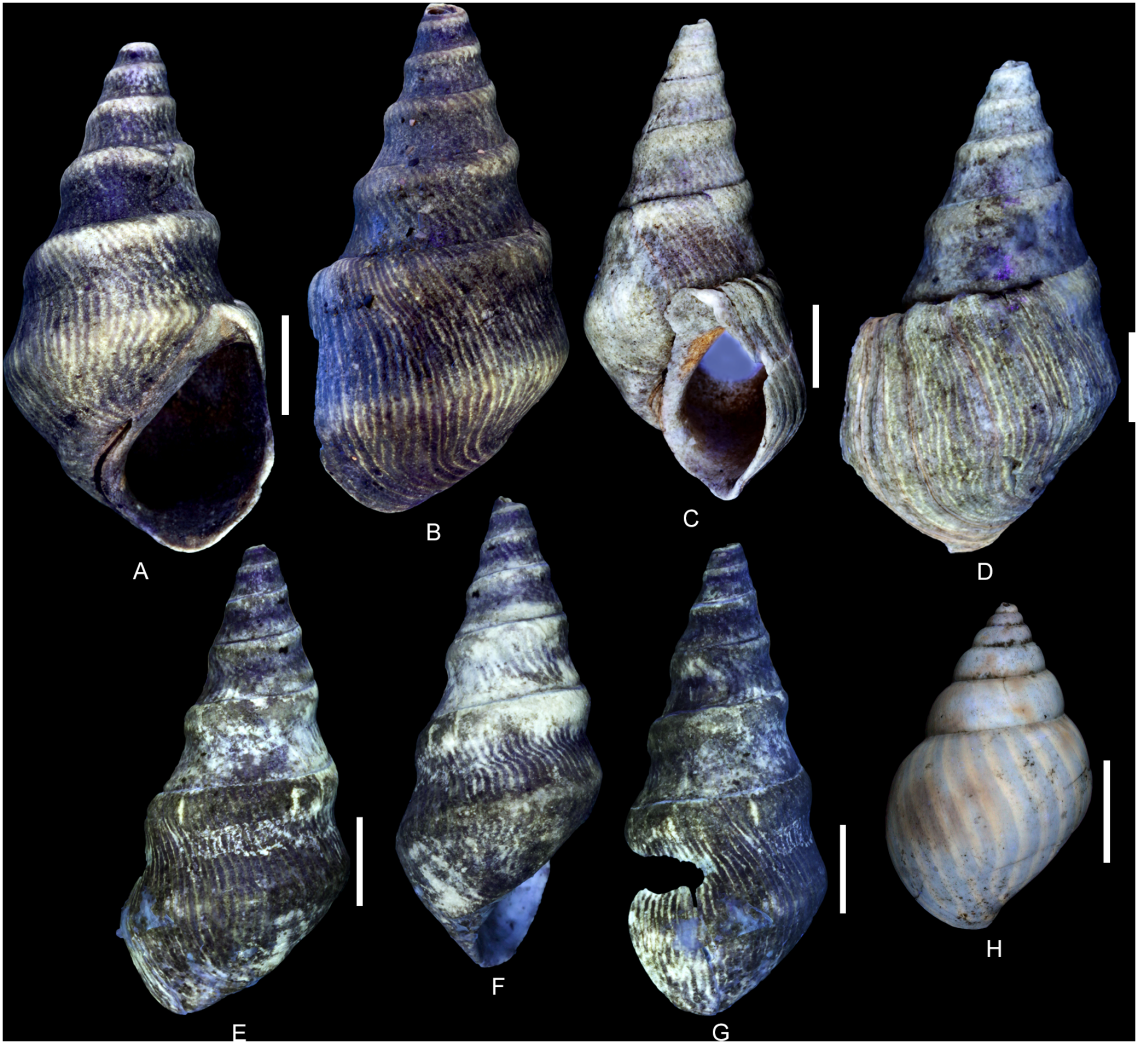
Residual colour pattern of *Cloughtonia abbreviata* (Römer, 1836) from the Oxfordian of Cordebugle (Calvados). (A-G) *Cloughtonia abbreviata*. (A, B) MNHN.F.J10209 (Cossmann coll.). (A) apertural view. (B) dorsal view. (C) MNHN.F.A32267 (Bigot coll.), aperturo-labral view. (D) MNHN.F.A32265 (Bigot coll.), dorsal view. (E-G) MNHN.F.A32266 (Bigot coll.). (E) dorsal view. (F) ablabral view. (G) labro-dorsal view. (H) *Pachycrommium* sp. (ampullinid) from the Ypresian of the Paris Basin showing similar axial stripes. MNHN.F.A30482 (Pacaud coll.), dorsal view. Scale bars: 10 mm.

Examined material: 1 spm (Cossmann coll.: MNHN.F.J10209) and 3 spm (Bigot coll.: MNHN.F.A32265, A32266, A32267).

The residual colour pattern consists of two components: (1) thin, axial, pale yellow, fluorescent stripes and (2) a dark, non-fluorescent background (Fig 11A–11G). The stripes are sigmoidal and approximately parallel to the growing edge. (Fig 11D). This residual pattern results from a roughly continuous incorporation of pigments over space and discontinuous recurrent incorporation over time (see S11 Fig).

Comments: The colour pattern seen in *C*. *abbreviata* under UV light confirms the observations of Eudes-Deslongchamps (1843) [39], who described narrow, axial stripes in a single specimen of *Melania condensata* in natural light. Originally, this exceptionally well preserved specimen was stored in the Laboratoire de Géologie de Caen, but is now considered lost, and was probably destroyed during World War II.

This colour pattern is very similar to that of the peculiar Ypresian (early Eocene) ampullinid species *Pachycrommium* sp. (Fig 11H, [20]). Considering its shell shape, the attribution of the latter species to *Pachycrommium* Woodring, 1828 is doubtful. However, despite the similarity of colour patterns in *Pachycrommium* sp. and *C*. *abbreviata*, these two species differ greatly with regard to shell shape. Moreover, the large time gap between their occurrences, spanning the entire Cretaceous, and the lack of data on colour patterns in ampullinids from this period prevent conclusions about the relationship between the two taxa to be drawn.

Comments on the genus *Cloughtonia*: (1) No colour pattern has been observed in *Cloughtonia michaelensis* (Buvignier, 1852) [57] from Cordebugle (see S1 Table). (2) The exact systematic position of *Cloughtonia* within the basal Caenogastropoda is still ambiguous. Earlier scholars [25, 52, 58–59] included species of *Cloughtonia* in the Pseudomelaniidae, while Szabó and Jaitly (2004) [60] and Gründel and Kaim (2006) [43] assigned them to the Ampullinidae. Indeed, the strange combination of a very elevated spire, strong nodulose carina, umbilicus, subsutural ramp, and slightly parasigmoid growth lines renders *Cloughtonia* an enigmatic taxon.

Comments on the colour patterns in Ampullinidae: Two distinct patterns have been revealed in the Jurassic ampullinids. (1) So far, the pattern of *Ampullina clio* seems unique in the Ampullinidae. Its absence in Cenozoic representatives of *Ampullina* Bowdich, 1822 may suggest a significant evolution of colour patterns within the genus, while the shell shape of Mesozoic and Cenozoic representatives remains very similar. (2) The colour pattern in *Cloughtonia abbreviata* is very similar to that in the pseudomelaniid *Pseudomelania collisa*. This pattern is rare in gastropods, and the co-occurrence in *Cloughtonia* and *Pseudomelania* may suggest that Ampullinidae and Pseudomelaniidae are closely related. Summing up the new information on Jurassic ampullinids, a considerable change in colour patterns is inferred within this family during the Mesozoic and Cenozoic.

Superfamily Cerithioidea Fleming, 1862

Family Procerithiidae Cossmann, 1906

Genus *Nerineopsis* Cossmann, 1906

*Nerineopsis boidini* (de Loriol, 1874) [52]

(Fig 12A and 12B)

**Fig 12:**
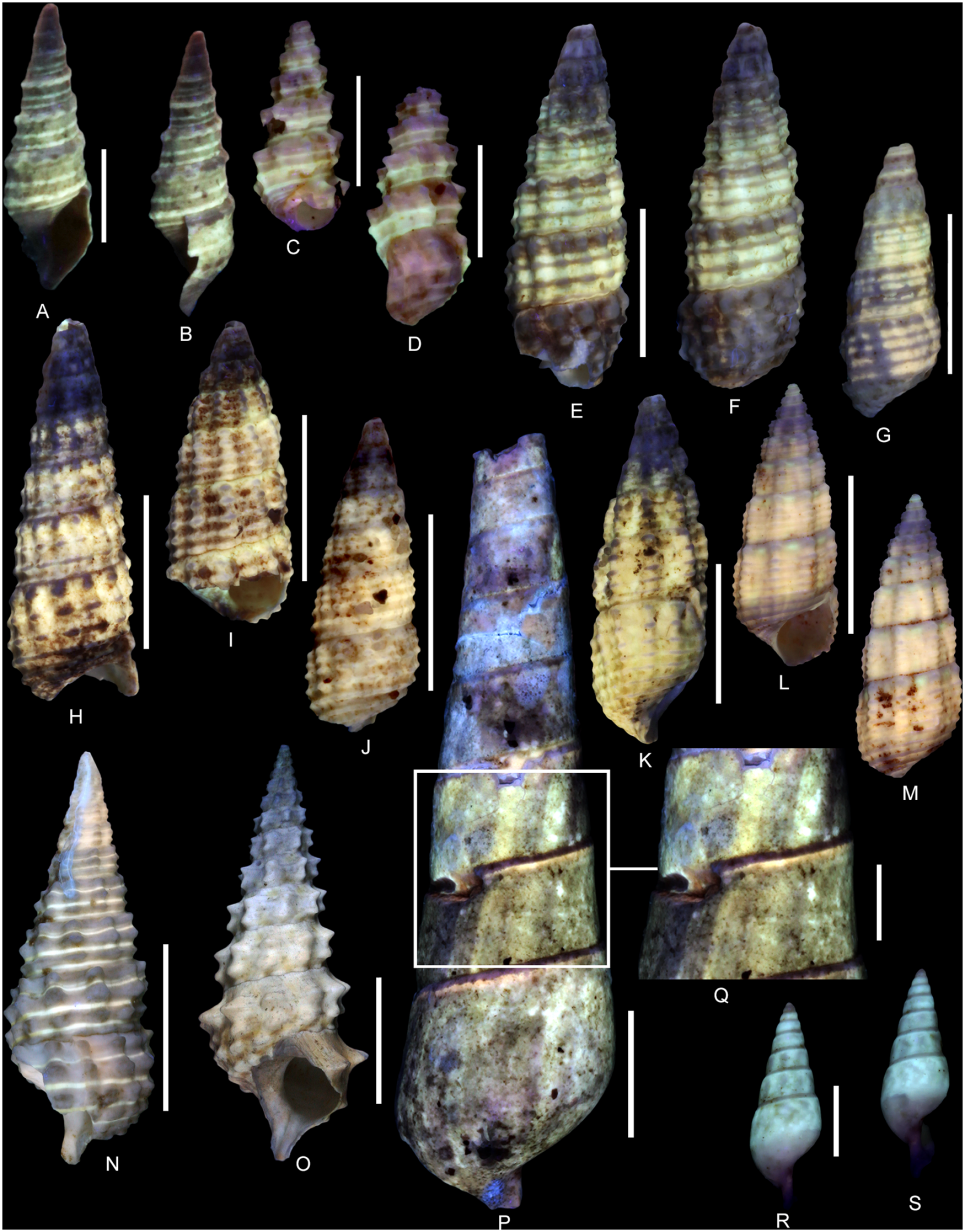
Residual colour patterns of gastropod species belonging to the clades Cerithioidea and Heterobranchia. **(A-M) Gastropods from the Oxfordian of Cordebugle (Calvados).** (A, B) *Nerineopsis boidini* (de Loriol, 1874), MNHN.F.J04464 (Cossmann coll.). (A) apertural view. (B) labral view. (C) *Paracerithium echinophorum* Cossmann, 1913, UPMC-141 (Le Marchand coll.), apertural view. (D) *P*. *echinophorum*, UPMC-142 (Le Marchand coll.), labral view. (E, F) *Exelissa diacritica* Cossmann, 1913, MNHN.F.J10445 (Cossmann coll.). (E) apertural view. (F) dorsal view. (G) *E*. *diacritica*, MNHN.F.J10443 (Cossmann coll.), dorsal view. (H) *E*. *diacritica*, MNHN.F.J10447 (Cossmann coll.), apertural view. (I) *E*. *diacritica*, MNHN.F.J10446 (Cossmann coll.) apertural view. (J) *E*. *diacritica*, MNHN.F.J11595 (Cossmann coll.), dorsal view. (K) *Exelissa distans* Cossmann, 1913, MNHN.F.A32270 (de Morgan coll.), ablabral view. (L, M) *E*. *distans*, UPMC-143 (Le Marchand coll.). (L) apertural view. (M) dorsal view. (N, O) Cerithioid gastropods from the Lutetian of the Paris basin for comparisons. (N) *Granulolabium (Granulolabium) multinodosum* (Deshayes, 1833), MNHN.F.A31733 (Faullummel coll.), labral view. (O) *Vicinocerithium calcitrapoides* (Lamarck, 1804), MNHN.F.A31096 (MNHN coll.), apertural view. (P-S) *Pseudonerinea caecilia* (d’Orbigny, 1851) from the Oxfordian of Calvados (Cordebugle). (P, Q) MNHN.F.J10466 (Cossmann coll.). (P) dorsal view. (Q) detailed view of the colour pattern. (R, S) MNHN.F.J11591 (Cossmann coll.). (R) dorso-ablabral view. (S) ablabral view. Scale bars: 10 mm (N, O, P), 5 mm (E-M, Q), 2 mm (A-D, R, S).

Examined material: 55 spm (Cossmann coll.: MNHN.F. J11071 and J10464).

Taxonomic note: de Loriol (1874, p. 66) [52] included this species in the genus *Cerithium* Bruguière, 1789. However, its sculpture is characteristic of the later established genus *Nerineopsis* Cossmann, 1906 and it is thus assigned *Nerineopsis* herein.

The residual colour pattern consists of two components: (1) spiral pale yellow, fluorescent spiral stripes and (2) a dark, non-fluorescent background (Fig 12A and 12B). On the spire, three thin straight stripes are present and the last whorl displays an additional stripe on the adbasal part (Fig 12A). All stripes are located on the nodular spiral cords. This pattern results from a discontinuous incorporation of pigments over space and continuous incorporation over time. Incorporation is restricted to distinct areas of the growing edge, which correspond to the locations of spiral sculpture (see S12 Fig).

Genus *Paracerithium* Cossmann, 1902

*Paracerithium echinophorum* Cossmann, 1913 [61]

(Fig 12C and 12D)

Examined material: 6 spm (Le Marchand coll.: UPMC, including two figured specimens UPMC-141 and 142).

The residual colour pattern consists of two components: (1) three pale yellow, fluorescent, straight spiral stripes and (2) a dark, non-fluorescent background (Fig 12C and 12D). All stripes are located on nodose spiral cords. Only the most prominent cord, situated in the adapical part of the whorl, does not emit fluorescence under UV light. The two adbasal stripes are similar in thickness and broader than the third, further adapical one, which is often also less fluorescent and occasionally absent on the early whorls (Fig 12C). This pattern results from a discontinuous incorporation of pigments over space, restricted to three distinct areas at the growing edge, and continuous incorporation over time. Although all three incorporation areas correspond to the spiral sculpture not all cords are associated with incorporation areas (see S13 Fig).

Genus *Exelissa* Piette, 1860

*Exelissa diacritica* Cossmann, 1913 [61]

(Fig 12E–12J)

Examined material: 43 spm (Cossmann coll.: MNHN.F.J10442, J10444, J10445, J10446, J10447 and J11069), 1 spm (UPMC coll.).

The residual colour pattern consists of two components: (1) three to four pale yellow, fluorescent, spiral stripes and (2) a dark, non-fluorescent background (Fig 12E–12G). The stripes are straight and located between the spiral cords. This pattern results from discontinuous incorporation of pigments over space, occurring in three to four distinct areas of the growing edge, and continuous incorporation over time. The distribution and size of the incorporation areas correlate with the interspaces of spiral cords (see S14 Fig).

Variability: Several specimens show a uniform pale fluorescence on the last four whorls (Fig 12H–12J). This pattern, which is identical to that of *E*. *distans* Cossmann, 1913 (Fig 12K–12M), is produced by continuous incorporation of pigments over space, along the entire growing edge, and continuous incorporation over time.

Comments: Taking the excellent preservation of the specimens into account, we consider the two distinct colour pattern morphologies observed in *E*. *diacritica* as a matter of intraspecific variability rather than of taphonomy. This kind of intraspecific variability, caused by the coalescence of coloured elements, is common in gastropods. In numerous Cenozoic species, individuals may either develop coloured spiral stripes or uniformly coloured whorls [20].

However, coloured spiral stripes that are located between spiral cords have been documented so far only from a single fossil species, i.e. the potamidid *Granulabium (s*.*s) multinodosum* (Deshayes, 1833) from the Lutetian of the Paris Basin (Fig 12N).

*Exelissa distans* Cossmann, 1913 [61]

(Fig 12K–12M)

Examined material: 1 spm (de Morgan coll.: MNHN.F.A32270), 6 spm (Le Marchand coll.: UPMC, including one figured specimen UPMC-143), 1 spm (Curet coll.: UPMC).

The residual colour pattern consists of two components: (1) dark non-fluorescent first whorls and (2) uniformly pale yellow and fluorescent last whorls (Fig 12K–12M). This pattern results from continuous incorporation of pigments over space and time starting at a late stage in ontogeny (see S15 Fig).

Comments: Uniformly fluorescent whorls are also documented in Cenozoic fossil gastropods, e.g. in *Vicinocerithium calcitrapoides* (Lamarck, 1804) from the Lutetian of the Paris Basin (Fig 12O).

Comments on *Exelissa*: Some specimens of *E*. *diacritica* show the same colour pattern observed in *E*. *distans*. However, none of the studied shells of *E*. *distans* have revealed fluorescent spiral stripes under UV light. Considering that only a few shells of *E*. *distans* were available for the present study and that these two species are very closely related, it would not be unexpected that both species would show similar intraspecific variability.

Clade Heterobranchia Burmeister, 1837

Family Ceritellidae Wenz, 1938

Genus *Pseudonerinea* de Loriol, 1890

*Pseudonerinea caecilia* (d’Orbigny, 1851) [32]

(Fig 12P–12S)

Examined material: 13 spm (Cossmann coll.: MNHN.F.J10465, J10466, J11591, J11592 and J08532), 3 spm (Bigot coll.: MNHN.F.A46317) and 2 spm (Curet coll.: UPMC).

The residual colour pattern consists of two components: (1) pale yellow, fluorescent, axial stripes and (2) a dark, non-fluorescent background (Fig 12P–12S). The stripes are slightly opisthocline, straight, parallel and of variable thickness. This pattern results from continuous incorporation of pigments over space, along the entire growing edge, and discontinuous and recurrent incorporation over time (see S16 Fig).

Variability: In juveniles (Fig 12R and 12S), the stripes may vary from opisthocline and straight to opisthocyrt and chevron-shaped.

Comments: Ontogenetic variability of shell colour patterns, as seen in *P*. *caecilia*, is common in Cenozoic gastropods.

Class Bivalvia Linnaeus, 1758

Clade Palaeoheterodonta Newell, 1965

Family Myophorellidae Kobayashi, 1954

Genus *Myophorella* Bayle, 1878

*Myophorella nodulosa* (Lamarck, 1801) [62]

(Fig 13A–13C)

**Fig 13:**
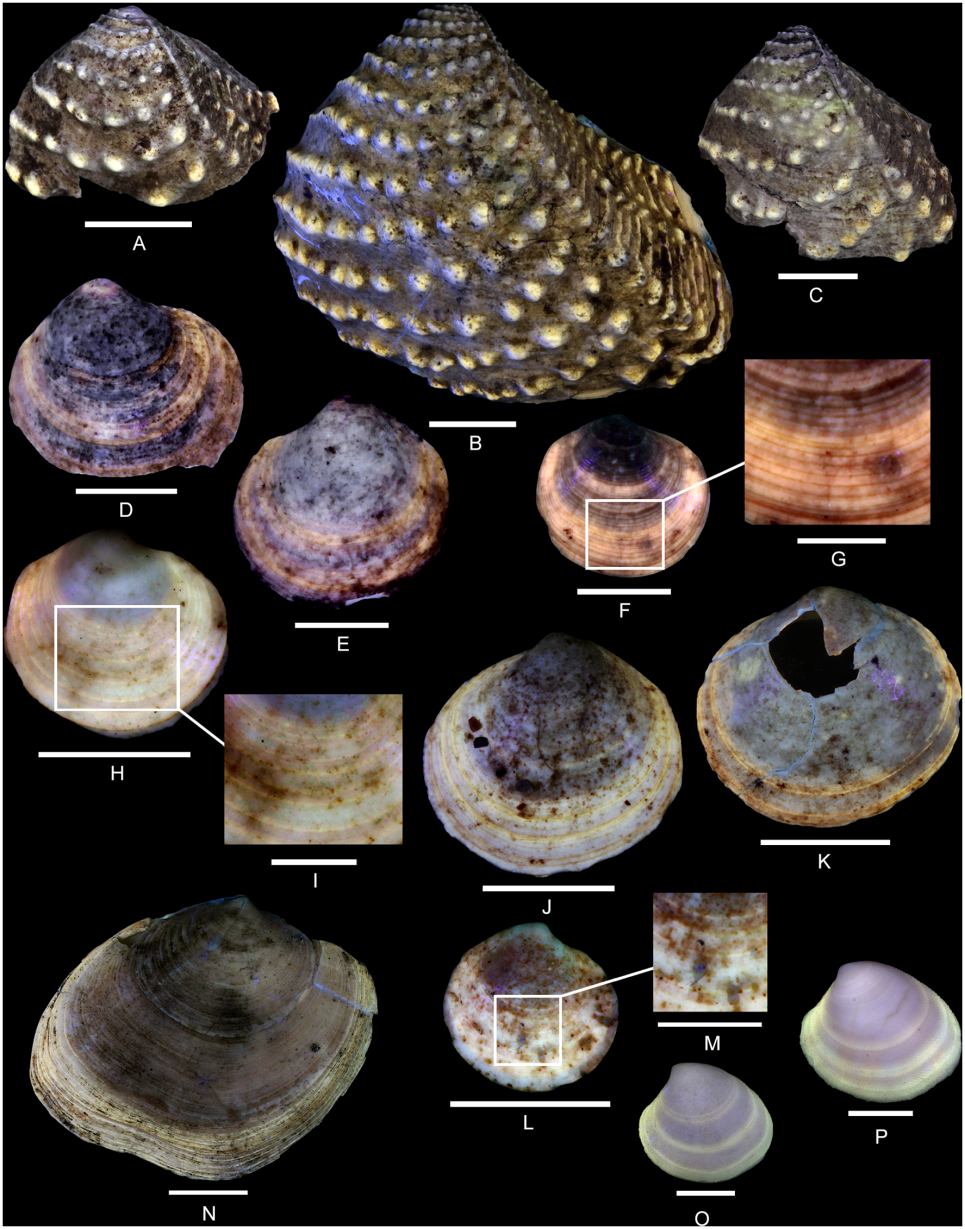
Residual colour patterns of bivalve species (Trigonioidea and Lucinoidea) from the Oxfordian of Cordebugle (Calvados). (A-M) Bivalves from the Oxfordian of Calvados (Cordebugle). (A) *Myophorella nodulosa* (Lamarck, 1801), UPMC-139 (UPMC coll.), left valve view. (B) *M*. *nodulosa*, UPMC-138 (UPMC coll.), left valve view. (C) *M*. *nodulosa*, UPMC-140 (UPMC coll.), left valve view. (D) *Mesolinga typica* Chavan, 1952, MNHN.F.J11589 (Cossmann coll.), left valve view. (E) *M*. *typica*, MNHN.F.J11590 (Cossmann coll.), right valve view. (F, G) *Mesomiltha pulchra* (Zittel and Goubert, 1861), UPMC-144 (Le Marchand coll.). (F) left valve view. (G) detailed view of the colour pattern. (H, I) *Jagonoma circumcisa* (Zittel and Goubert, 1861), MNHN.F.J11586 (Cossmann coll.). (H) right valve view. (I) detailed view of the colour pattern. (J) *J*. *circumcisa*, MNHN.F.J11588 (Cossmann coll.), left valve view. (K) *J*. *circumcisa*, MNHN.F.J11587 (Cossmann coll.), left valve view. (L, M) *J*. *circumcisa*, MNHN.F.J11585 (Cossmann coll.). (L) right valve view. (M) detailed view of the colour pattern. (N-P) Bivalves from the Lutetian of the Paris basin showing similar commarginal stripes. (N) *Pseudomiltha (Pseudomiltha) mutabilis* (Lamarck, 1807), MNHN.F.A25068 (Faullummel coll.), left valve view. (O, P) *Katelysia (Textivenus) scobinellata* (Lamarck, 1806). (O) MNHN.F.A31917 (Faullummel coll.), right valve view. (P) MNHN.F.A31910 (Faullummel coll.), right valve view. Scale bars: 10 mm (A-C, J, K, N), 5 mm (D-F, H, L, O, P), 2 mm (G, I, M).

Examined material: 3 spm (Raynaud coll.: MNHN.F.A46229), 2 spm (Le Marchand coll.: UPMC), 10 spm (UPMC coll., including three figured specimens UPMC-138, 139 and 140).

Taxonomic note: Based on the observations of Francis (2000) [63] and the present authors, *Trigonia bronnii* Agassiz, 1840, as well as *Trigonia clavellata* Parkinson, 1811 are herein considered junior synonyms of *Myophorella nodulosa* (Lamarck, 1801) [62]. Specimens described under these three species names overlap in time, and range well within the moderate intraspecific variability of *Myophorella nodulosa*.

The residual colour pattern consists of two components: (1) pale yellow, fluorescent patches and (2) a dark, non-fluorescent background (Fig 13A–13C). The patches are circular to slightly elliptical and their size increases with shell growth. They are located on the tubercles which are arranged in arcuate, subcommarginal to oblique rows. This pattern results from discontinuous incorporation of pigments both over space and time. The distribution and the size of the incorporation areas along the growing edge are in phase with the sculpture.

Variability: One of the specimens also shows fluorescent segments on the commarginal lirae (Fig 13B), emphasizing the correlation of sculpture and colour pattern (see S17 Fig).

Clade Heterodonta Neumayr, 1884

Family Lucinidae Fleming, 1828

Genus *Mesolinga* Chavan, 1951

*Mesolinga typica* Chavan, 1952 [29]

(Fig 13D and 13E)

Examined material: 19 spm (Cossmann coll.: MNHN.F.J10401, J11589 and J11590).

The residual colour pattern consists of two components: (1) two broad, commarginal, pale, yellow-orange, fluorescent stripes and (2) a dark, non-fluorescent background (Fig 13D and 13E). The stripes are located in the median and ventral parts of the valves. This pattern is caused by continuous incorporation of pigments over space, along the entire growing edge, and discontinuous and recurrent incorporation over time (see S18 Fig).

Variability: One of the two figured specimens (Fig 13D) shows an additional narrow stripe closer to the umbo.

Comments: The fluorescent commarginal stripes directly succeed major growth interruptions.

Genus *Mesomiltha* Chavan, 1938

*Mesomiltha pulchra* (Zittel and Goubert, 1861) [64]

(Fig 13F and 13G)

Examined material: 1 spm (Cossmann coll.: MNHN.F.J11584) and 3 spm (Le Marchand coll.: UPMC, including one figured specimen UPMC-144).

The residual colour pattern consists of two components: (1) three commarginal, pale yellow-orange, fluorescent stripes and (2) a dark, non-fluorescent background (Fig 13F and 13G). Two narrow stripes occur in the median part of the valve and a third very broad stripe is situated in the ventral part. This pattern results from continuous incorporation of pigments over space, along the entire growing edge, and discontinuous and recurrent incorporation over time (see S19 Fig).

Comments: As in *Mesolinga typica*, the fluorescent commarginal stripes directly succeed major growth interruptions.

Genus *Jagonoma* Chavan, 1946

*Jagonoma circumcisa* (Zittel and Goubert, 1861) [64]

(Fig 13H–13M)

Examined material: 6 spm (Cossmann coll.: MNHN.F. J11583, J11585, J11586, J11587 and J11588).

The residual colour pattern consists of two components: (1) pale yellow, fluorescent, commarginal stripes and (2) a dark, non-fluorescent background (Fig 13H–13M). The number of stripes, as well as the distances between them, is variable. There is no stripe on the umbo or in the umbonal region. This pattern is produced by continuous incorporation of pigments over space and discontinuous and recurrent incorporation over time (see S20 Fig).

Comments: As seen in the juvenile specimen (Fig 13L) the onset of fluorescent stripes occurs at a much earlier ontogenetic stage than expressed in adult shells (Fig 13J and 13K). Likely, this is a matter of preservation, since pigments are usually incorporated only in the outermost layers of the shell, which often become slightly abraded in the umbonal regions of burrowing bivalves. Similar to the aforementioned lucinids, the commarginal fluorescent stripes directly succeed major growth interruptions.

Comments on Lucinidae: The colour pattern revealed in Oxfordian lucinid bivalves from Cordebugle seems to be common in bivalves. Numerous Cenozoic species display coloured commarginal stripes that directly succeed growth interruptions (Fig 13N–13P, [46]).

Clade Archiheterodonta Giribet, 2007

Family Astartidae d’Orbigny, 1844

Genus *Neocrassina* Fischer, 1886

*Neocrassina ovata* (Smith, 1817) [65]

(Fig 14A–14C)

**Fig 14:**
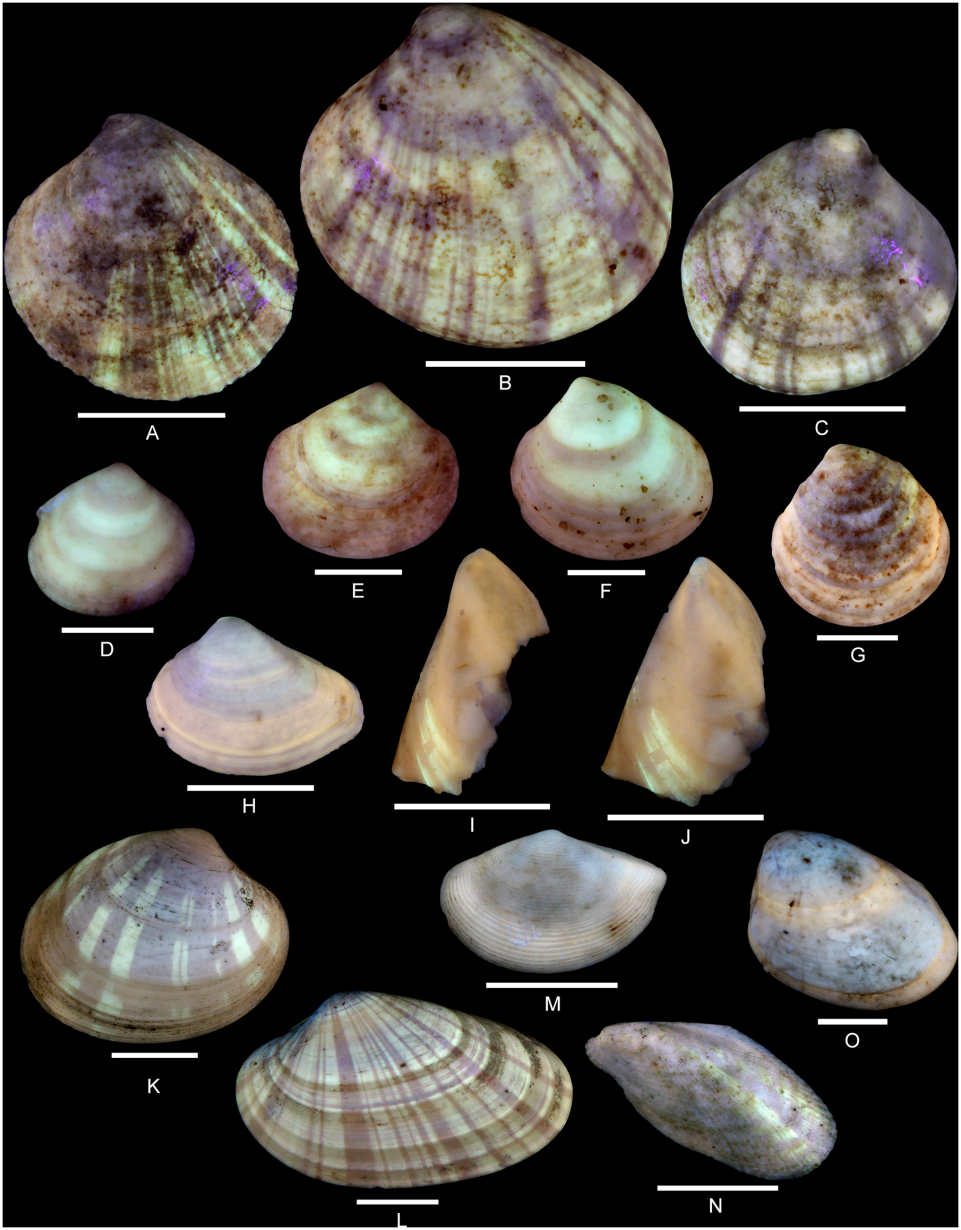
Residual colour patterns of bivalve species (Crassatelloidea, Sphaerioidea and Mytiloidea) from the Oxfordian of Calvados. (A-J) Bivalves from the Oxfordian of Cordebugle (Calvados). (A-C) *Neocrassina ovata* (Smith, 1817). (A) MNHN.F.J10472 (Cossmann coll.), left valve view. (B) MNHN.F.J10473 (Cossmann coll.), left valve view. (C) MNHN.F.J11075 (Cossmann coll.), right valve view. (D) *Nicaniella communis* (Zittel and Goubert, 1861), MNHN.F.J10474 (Cossmann coll.), left valve view. (E, F) *Nicaniella bruni* Chavan, 1952. (E) UPMC-145 (UPMC coll.), right valve view. (F) UPMC-146 (UPMC coll.), left valve view. (G) *Nicaniella morini* (de Loriol, 1875), UPMC-147 (UPMC coll.), left valve view. (H) *Neomiodon percrassus* Chavan, 1945, UPMC-148 (UPMC coll.), left valve view. (I, J) *Modiolus imbricatus* Sowerby, 1818, UPMC-149 (UPMC coll.). (I) left valve view. (J) view of the anterior part of the valve. (K-O) Bivalves from the Lutetian of the Paris basin showing similar radial stripes. (K) *Pitar (Paradione) lunularia* (Deshayes, 1825), MNHN.F.A25079 (Faullummel coll.), right valve view. (L) *Costacallista laevigata* (Lamarck, 1806), MNHN.F.A25081 (Faullummel coll.), left valve view. (M) *Nuculana (Saccella) striata* (Lamarck, 1805), MNHN.F.A31834 (Faullummel coll.), left valve view. (N) *Musculus (Planimodiola) sulcatus* (Lamarck, 1805), MNHN.F.A31975 (Faullummel coll.), left valve view. (O) *Nuculana (Nuculana) terminalis* Deshayes, 1858, MNHN.F.A31835 (Faullummel coll.), left valve view. Scale bars: 10 mm (A-C, K, L, N), 5 mm (H-J, M, O), 2 mm (D-G).

Examined material: 6 spm (Cossmann coll.: MNHN.F.J11073, J11075, J10472 and J10473).

Taxonomic note: *Neocrassina* has been raised to genus level by Chavan (1969) [66].

The residual colour pattern consists of two components: (1) pale yellow, fluorescent, radial stripes and (2) a dark, non-fluorescent background (Fig 14A–14C). The stripes are continuous from the umbo to the ventral margin. They are closely spaced, and of varied width. The broadest stripes are located on the median part of the valve. This pattern results from discontinuous incorporation of pigments over space, and continuous incorporation over time. The numerous incorporation areas of varied size are distributed along the entire growing edge (see S21 Fig).

Variability: Some specimens show radial rows of broad, subquadrangular, fluorescent patches (Fig 14B and 14C). These rows result from discontinuous incorporation of the pigments over time.

Comments: The colour pattern revealed in *N*. *ovata* is common in bivalves. Numerous Cenozoic species display a pattern of radial stripes or rows of subquadrangular patches (Fig 14K, 14L and 14N).

Genus *Nicaniella* Chavan, 1945

*Nicaniella communis* (Zittel and Goubert, 1861) [64]

(Fig 14D)

Examined material: 69 spm (Cossmann coll.: MNHN.F.J10474 and J10475), 7 spm (UPMC coll.).

Taxonomic note: *Nicaniella* has been raised to genus level by Chavan (1969) [66].

The residual colour pattern consists of two components: (1) pale yellow, fluorescent, commarginal stripes and (2) a dark, non-fluorescent background. The figured specimen (Fig 14D) shows four relatively broad commarginal stripes with less broad interspaces. This pattern is produced by continuous incorporation of pigments over space, along the entire growing edge, and discontinuous and recurrent incorporation over time (see S22 Fig).

Variability: The examined shells exhibit two, three or four broad fluorescent stripes.

Comments: The onset of pigment incorporation occurs early in ontogeny.

*Nicaniella bruni* Chavan, 1952 [29]

(Fig 14E and 14F)

Examined material: 20 spm (UPMC coll., including two figured specimens UPMC-145 and 146).

The residual colour pattern consists of two components: (1) two or three pale yellow, fluorescent, broad, commarginal stripes and (2) a dark, non-fluorescent background (Fig 14E and 14F). This pattern is produced by continuous incorporation of pigments over space, along the entire growing edge, and discontinuous and recurrent incorporation over time (see S23 Fig).

Comments: The onset of pigment incorporation occurs early in ontogeny.

*Nicaniella morini* (de Loriol, 1875) [67]

(Fig 14G)

Examined material: 16 spm (Cossmann coll.: MNHN.F.J10427 and J10428) and 6 spm (UPMC coll., including one figured specimen UPMC-147).

The residual colour pattern consists of two components: (1) a single broad, pale yellow-orange, slightly fluorescent, commarginal stripe and (2) a dark, non-fluorescent background on the umbo and dorsal half of the valve (Fig 14G). This pattern results from continuous incorporation of pigments over space and time (S24 Fig).

Comments: In contrast to the other species of *Nicaniella*, incorporation of pigments in *N*. *morini* occurs only at a late stage of ontogeny. All the valves that emit fluorescence under UV light show the same colour pattern independent of size. Consequently, the incorporation seems to have persisted once the individuals had gained maturity.

Comments on the *Nicaniella*: The colour patterns revealed in different species of *Nicaniella* from Cordebugle are common in bivalves. Numerous Cenozoic species show a single or several coloured commarginal stripes (Fig 14M and 14O).

Clade Neoheterodontei J. Taylor et al. 2007

Family Neomiodontidae Casey, 1955

Genus *Neomiodon* Fischer, 1887

*Neomiodon percrassus* Chavan, 1945 [27]

(Fig 14H)

Examined material: 13 spm (Cossmann coll.: MNHN.F.J10476) and 22 spm (UPMC coll., including one figured specimen UPMC-148).

The residual colour pattern consists of two components: (1): pale yellow, fluorescent, commarginal stripes and (2) a dark, non-fluorescent background (Fig 14H). A narrow stripe is positioned on the dorsal part of the valve and a very broad stripe covers almost the entire ventral half; the umbo and most of the dorsal half of the valve are dark. This pattern results from continuous incorporation of pigments over space, and discontinuous and recurrent incorporation over time (see S25 Fig).

Comments: The narrow dorsal stripe is frequently absent while all valves show a broad, fluorescent ventral stripe. Therefore, the usual pattern consists of a single broad ventral stripe and the incorporation of pigments commonly starts relatively late in ontogeny.

Clade Pteriomorphia Beurlen, 1944

Family Mytilidae Rafinesque, 1815

Genus *Modiolus* Lamarck, 1799

*Modiolus imbricatus* J. Sowerby, 1818 [68]

(Fig 14I and 14J)

Examined material: 3 spm (UPMC coll., including one figured specimen UPMC-149).

The residual colour pattern consists of two components: (1) commarginal pale yellow fluorescent segments and (2) a dark, non-fluorescent background (Fig 14I and 14J). This pattern results from a discontinuous incorporation of pigments over space, along large areas of the growing edge, and discontinuous and recurrent incorporation over time.

Comments: Only two fragmentary specimens have shown elements of pattern under UV light. Therefore, the description of the colour pattern is incomplete.

## Discussion

### Preservation of colour patterns in fossil shells

The reconstruction of the evolutionary history of shell colour patterns is generally considered to be limited by the scarcity of material with preserved colouration. However, the lack of comprehensive research in this field is certainly another factor limiting the establishment of pathways in colour pattern evolution. Numerous scientists have shown that UV light is an easy means to visualise residual colour patterns in Cenozoic shells. In Cenozoic fossil molluscs, residual colour preservation is so common that it is frequently possible to study the intraspecific variability of colour patterns [18]. Nevertheless, nobody has conducted systematic investigations for residual colouration in Mesozoic shells.

The probability to observe residual colour patterns under UV light depends on the diagenetic state of the shells (ideally pristine aragonite or calcite), and thus also on the properties of the sediments in which the shells are enclosed. These sedimentary properties, however, are inadequately known. Obviously, localities that have these properties are less common in the Palaeozoic or Mesozoic than in the Cenozoic record, but may provide key points for the study of the early evolution of residual colour patterns. The Oxfordian Cordebugle fauna is the first Mesozoic assemblage from which a considerable diversity of residual colour patterns is documented using UV light and thus certainly is such a key point. In addition, it demonstrates that the preservation of colour patterns may be rather common in pre-Cenozoic shells, provided that they are well preserved (S1 Table).

### Diversity of the residual colour patterns

Nine different colour patterns have been distinguished in the 25 species that provided positive results under UV light (54% of the tested species; S1 Table).

#### Gastropods

From the 28 species that were tested, 14 species belonging to six families yielded positive results (50% of the tested species; S1 Table). Six colour patterns have been revealed under UV light (patterns 1G, 2G, 4G, 5G, 6G and 8G) and two are observed in natural light (patterns 3G and 7G).

Pattern 1G: Triangular, dark false patches, contrasting with fluorescent colouration have been observed in *Pseudomelania cornelia* (Pseudomelaniidae; Fig 8). This pattern is abundant in numerous phylogenetically distant Cenozoic gastropods (e.g., Volutoidea and Seraphsidae).

Pattern 2G: Fluorescent, spiral stripes, located on spiral cords, have been observed in *Gerasimovcyclus* cf. *lorioli* (Eucyclidae; Figs 3D, 5F and 5G), *Nerineopsis boidini*, *Paracerithium echinophorum* and *P*. *climacinum* (Procerithiidae; Fig 12A–12D and 12N). This pattern also occurs in numerous phylogenetically distant Cenozoic gastropods (e.g., Trochidae, Cerithiidae, Buccinidae, Fasciolariidae).

Pattern 3G (observed in natural light): Spiral stripes that are unrelated to the sculpture occur in *Neridomus ovula* (Neritidae; Fig 5I–5J). This is one of the most widespread colour patterns in Cenozoic gastropods (e.g., Neritoidea, Fissurelloidea, Cerithioidea, Buccinoidea, Conoidea, Acteonoidea). Therefore, this pattern can be considered as either plesiomorphic or highly convergent.

Pattern 4G: Meshwork pattern has been observed in *Pseudomelania brasili* and *Ampullina clio* (Figs 6 and 10). It is a rather uncommon pattern shared by Jurassic pseudomelaniids and ampullinids (basal Caenogastropoda). In Cenozoic gastropods, this scarce pattern has so far only been revealed in the representatives of the seraphsid genus *Diameza* Deshayes, 1865 (Fig 10N–10P).

Pattern 5G: Fluorescent axial stripes have been observed in members of three different gastropod families, i.e. *Pseudomelania collisa* (Pseudomelaniidae; Fig 9), *Cloughtonia abbreviata* (Ampullinidae; Fig 11) and *Pseudonerinea caecilia* (Ceritellidae; Fig 12P–12S). The ceritellid *Pseudonerinea caecilia* shows widely spaced, regular stripes, while the pseudomelaniid *Pseudomelania collisa* and the ampullinid *Cloughtonia abbreviata* share a very similar pattern, consisting of more irregular, very closely spaced axial stripes. This latter pattern is rather uncommon in gastropods. Since both patterns 4G and 5G are uncommon but shared by Jurassic pseudomelaniids and ampullinids (basal Caenogastropoda), they might suggest close relationships between these two families. Considering the uncertain relationships of these families with other basal Caenogastropoda, further studies focusing on the colour patterns of their representatives might be rewarding.

Pattern 6G: Fluorescent spiral stripes located between the spiral cords or uniformly fluorescent whorls occur in the Jurassic Procerithiidae *Exelissa diacritica* and *E distans* (Fig 12E–12M), as well as in Cenozoic potamidids (both Cerithioidea).

Pattern 7G (observed in natural light): Opisthocline zigzag stripes have been observed in *Neridomus* sp. (Neritidae; Fig 5K) and represent a common pattern in Cenozoic neritids.

Pattern 8G: Irregular dark patches contrasting with fluorescent colouration occur in *Ataphrus (Ataphrus) marschmidti* and *A*. *(Endianaulax) sarahae* (Ataphridae; Fig 5A–5E). Since this pattern has not been observed in any Cenozoic Vetigastropoda, including ataphrids, it is currently regarded as a genuine feature of the Jurassic Ataphridae. *Ataphrus (A*.*) griffini* Dockery, 1993, from the Campanian of Mississippi, has a colour pattern visible in natural light composed of a pale spiral stripe and dark straight to zigzag axial stripes.

#### Bivalves

From the 18 bivalve species studied, 11 species belonging to five families (61% of the tested species; S1 Table) yielded positive results. Three distinct colour patterns have been observed.

Pattern 1B: Fluorescent commarginal stripes occur in *Mesolinga typica*, *Mesomiltha pulchra*, *Jagonoma circumcisa* (all Lucinidae, Fig 13D–13M), *Nicaniella communis*, *N*. *bruni*, *N*. *morini* (all Astartidae, Fig 14D–14G), *Neomiodon percrassus* and *N*. *ovatostriatus* (both Neomiodontidae, Fig 14H). This pattern is also very common in Cenozoic bivalves (e.g. Nuculoidea, Tellinoidea and Veneroidea).

Pattern 2B: Fluorescent radial stripes have been observed in *Neocrassina ovata* (Astartidae, Fig 14A–14C) and are also very common in Cenozoic species of various superfamilies (e.g., Crassatelloidea, Lucinoidea, Veneroidea).

Pattern 3B: Subcommarginal or oblique rows of fluorescent patches that are located on shell tubercles occur only in *Myophorella nodulosa* (Myophorellidae; Fig 13A–13C).

In summary, eleven colour patterns have been observed in the Jurassic shells from Cordebugle. Several of them occur in species of numerous unrelated families (patterns 1G, 2G and 3G of gastropods, patterns 1B and 2B of bivalves). These “general patterns” have been considered as such already with regard to Cenozoic shells. Moreover, general patterns in bivalves have been reported from as early as the Carboniferous, and are thus clearly an ancient feature (pattern 1B [1, 69]; pattern 2B [70–71]).

Several other colour patterns are restricted to species of a few families. They may thus be of considerable interest with regard to phylogeny and systematics. Such rare colour patterns are much more abundant and diverse in gastropods than in bivalves from Cordebugle. Although their taxonomic composition is obviously different from that of the Jurassic fauna, a similar discrepancy has been reported for Cenozoic mollusc assemblages and may thus constitute another ancient feature that had been established at least by the Late Jurassic. As a further important result, the data from Cordebugle demonstrate that a significant diversification of shell colour patterns in gastropods had already occurred by the Oxfordian.

### Yellow versus red fluorescence under UV light

Two distinct types of fluorescence, i.e. different wavelengths, are emitted by the shells from Cordebugle, when exposed to UV light. All Caenogastropoda (Figs 6 to 12A–12M) and Heterobranchia (Fig 12P–12S) and all Bivalvia (Figs 13 and 14) emit a whitish-beige to yellow fluorescence. Two gastropod species belonging to the Vetigastropoda (Fig 5A–5E) emit red fluorescence. As an exception, *Gerasimovcyclus* cf. *lorioli* emits yellow fluorescence. Previous studies of residual colour patterns in Cenozoic molluscs have shown that all members of the Vetigastropoda exhibit red fluorescence, while those belonging to other gastropod clades, as well as all bivalves, exhibit whitish-beige to yellow fluorescence (S2 Table; [18, 22, 33, 47–48]). Members of the Neritimorpha display no fluorescence, but commonly preserve colour patterns visible in natural light. Taking the results from both Mesozoic and Cenozoic shells into account, red fluorescence under UV light may be considered as a diagnostic criterion of fossil Vetigastropoda. It may thus help to place several of the numerous Mesozoic taxa of doubtful affinities in the correct systematic position. Most likely, the different types of fluorescence result from the biochemical diversity of the pigments involved in the coloration of the gastropod shells [49–50, 72]. Unfortunately, the nature and composition of pigments in mollusc shells, whether fossil or Recent, are still poorly established, and Comfort’s (1951) statement that “the coloured substances which occur in molluscs offer a remarkably wide and largely unworked field to the biochemist” is regrettably still true [72].”

## Conclusion

The mollusc fauna from the Oxfordian of Cordebugle (Calvados, western France) is the first diverse pre-Cenozoic mollusc assemblage that has been successfully tested for fluorescent shell colour patterns, using UV light. The preservation of these colour patterns is certainly linked to the exceptional preservation of the mollusc shells, which still consist of pristine aragonite. In addition, the residual colour patterns revealed in the gastropod shells from Cordebugle are approximately 100 Myr older than the earliest previously documented ones, which come from the Early Paleogene (Thanetian).

The innovative application of UV light has allowed for the documentation of colour patterns in species that belong to large clades without Cenozoic representatives, such as the Nerineoidea or Myophorelloidea. Large numbers of well preserved shells have even enabled a study of the intraspecific variability of colour patterns in several gastropod species. Moreover, the results from the Cordebugle assemblage facilitate the analysis of colour pattern evolution, e.g. in the family Ampullinidae.

At the large scale, the Cordebugle Konservat Lagerstätte certainly yielded a key assemblage for the understanding of the evolution of colour patterns in Mesozoic molluscs, since 25 out of 46 species that have been tested reveal fluorescent residual colour patterns. The diversity of the patterns observed clearly demonstrates: (1) a significant diversification of colour patterns in gastropods as early as the Late Jurassic; (2) the importance of different wavelengths of fluorescence as a taxonomic tool (appearing as red *versus* whitish-beige to yellow colours under UV light); and (3) that residual colour patterns in Mesozoic shells may provide valuable information for the distinction of taxa, especially when they are almost identical with regard to shell shape (e.g., *Pseudomelania* spp.), as already seen for Cenozoic taxa.

Although the successful use of UV light to reveal residual colouration in Mesozoic shells has only been applied to material from the locality of Cordebugle herein, well preserved shells have been described from several other Jurassic and Cretaceous localities (e.g. the Middle to Late Jurassic of Poland, Lithuania and Russia and the Late Cretaceous of North America), and may also yield comparable colour patterns. Further study may thus not only improve the documentation of shell colour patterns from the Mesozoic, but also facilitate a better understanding of their evolution.

## Supporting Information

S1 Table Examined material from Oxfordian of Cordebugle and specimens emitting fluorescence under UV light. (XLS)

S2 Table French Mesozoic and Cenozoic species emitting red or yellow-white fluorescence under UV light. (XLS)

S1 Fig Mechanisms of incorporation of pigments in *Ataphrus (Ataphrus) marschmidti* Gründel and Kaim, 2006. Pattern 8G: irregular dark patches contrasting with fluorescent colouration.
(TIF)

S2 Fig Mechanisms of incorporation of pigments in *Ataphrus (Endianaulax) sarahae* (Chavan, 1954). Pattern 8G: irregular dark patches contrasting with fluorescent colouration.
(TIF)

S3 Fig The eucyclid *Gerasimovcyclus* cf. *lorioli* (Schmidt, 1905) and its shell microstructure. (A, B) UPMC-129 (Le Marchand coll.). (A) apertural view. (B) shell microstructure of the apertural margin. (C-E) UPMC-199 (Le Marchand coll.). (C) apertural view. (D) shell microstructure of the apertural margin. (E) detailed view of the photograph D. nl: nacreous layer.? opl: outer, probably prismatic layer. Scale bars: 2 mm (A, C), 100 μm (B, D, E).
(TIF)

S4 Fig Mechanisms of incorporation of pigments in *Gerasimovcyclus* cf. lorioli (Schmidt, 1905). Pattern 2G: fluorescent, spiral stripes, located on spiral cords.
(TIF)

S5 Fig Mechanisms of incorporation of pigments in *Neridomus ovula* (Buvignier, 1843). Pattern 3G (observed in natural light): spiral stripes.
(TIF)

S6 Fig Mechanisms of incorporation of pigments in *Neridomus* sp. Pattern 7G (observed in natural light): opisthocline zigzag stripes. (TIF)

S7 Fig Mechanisms of incorporation of pigments in *Pseudomelania brasili* (Bigot, 1938). Pattern 4G: meshwork.
(TIF)

S8 Fig Mechanisms of incorporation of pigments in *Pseudomelania cornelia* (d’Orbigny, 1851). Pattern 1G: triangular, dark false patches, contrasting with fluorescent colouration.
(TIF)

S9 Fig Mechanisms of incorporation of pigments in *Pseudomelania collisa* (de Loriol, 1874). Pattern 5G: fluorescent axial stripes.
(TIF)

S10 Fig Mechanisms of incorporation of pigments in *Ampullina clio* (d’Orbigny, 1850). Pattern 4G: meshwork.
(TIF)

S11 Fig Mechanisms of incorporation of pigments in *Cloughtonia abbreviata* (Römer, 1836). Pattern 5G: fluorescent axial stripes.
(TIF)

S12 Fig Mechanisms of incorporation of pigments in *Nerineopsis boidini* (de Loriol, 1874). Pattern 2G: fluorescent, spiral stripes, located on spiral cords.
(TIF)

S13 Fig Mechanisms of incorporation of pigments in *Paracerithium echinophorum* Cossmann, 1913. Pattern 2G: fluorescent, spiral stripes, located on spiral cords.
(TIF)

S14 Fig Mechanisms of incorporation of pigments in *Exelissa diacritica* Cossmann, 1913. Pattern 6G: fluorescent spiral stripes located between the spiral cords or uniformly fluorescent whorls.
(TIF)

S15 Fig Mechanisms of incorporation of pigments in *Exelissa distans* Cossmann, 1913. Pattern 6G: fluorescent spiral stripes located between the spiral cords or uniformly fluorescent whorls.
(TIF)

S16 Fig Mechanisms of incorporation of pigments in *Pseudonerinea caecilia* (d’Orbigny, 1851). Pattern 5G: fluorescent axial stripes.
(TIF)

S17 Fig Mechanisms of incorporation of pigments in *Myophorella nodulosa* (Lamarck, 1801). Pattern 3B: subcommarginal or oblique rows of fluorescent patches that are located on shell tubercles.
(TIF)

S18 Fig Mechanisms of incorporation of pigments in *Mesolinga typica* Chavan, 1952. Pattern 1B: fluorescent commarginal stripes.
(TIF)

S19 Fig Mechanisms of incorporation of pigments in *Mesomiltha pulchra* (Zittel and Goubert, 1861). Pattern 1B: fluorescent commarginal stripes.
(TIF)

S20 Fig Mechanisms of incorporation of pigments in *Jagonoma circumcisa* (Zittel and Goubert, 1861). Pattern 1B: fluorescent commarginal stripes.
(TIF)

S21 Fig Mechanisms of incorporation of pigments in *Neocrassina ovata* (Smith, 1817). Pattern 2B: fluorescent radial stripes.
(TIF)

S22 Fig Mechanisms of incorporation of pigments in *Nicaniella communis* (Zittel and Goubert, 1861). Pattern 1B: fluorescent commarginal stripes.
(TIF)

S23 Fig Mechanisms of incorporation of pigments in *Nicaniella bruni* Chavan, 1952. Pattern 1B: fluorescent commarginal stripes.
(TIF)

S24 Fig Mechanisms of incorporation of pigments in *Nicaniella morini* (de Loriol, 1875). Pattern 1B: fluorescent commarginal stripes.
(TIF)

S25 Fig Mechanisms of incorporation of pigments in *Neomiodon percrassus* Chavan, 1945. Pattern 1B: fluorescent commarginal stripes.
(TIF)
