# Supplementary figures and images for: UV Light Reveals the Diversity of Jurassic Shell Colour Patterns: Examples from the Cordebugle Lagerstätte (Calvados, France)
Source: PLoS One. 2015 Jun 3;10(6):e0126745. doi: 10.1371/journal.pone.0126745 (PMC4454669; doi:10.1371/journal.pone.0126745)

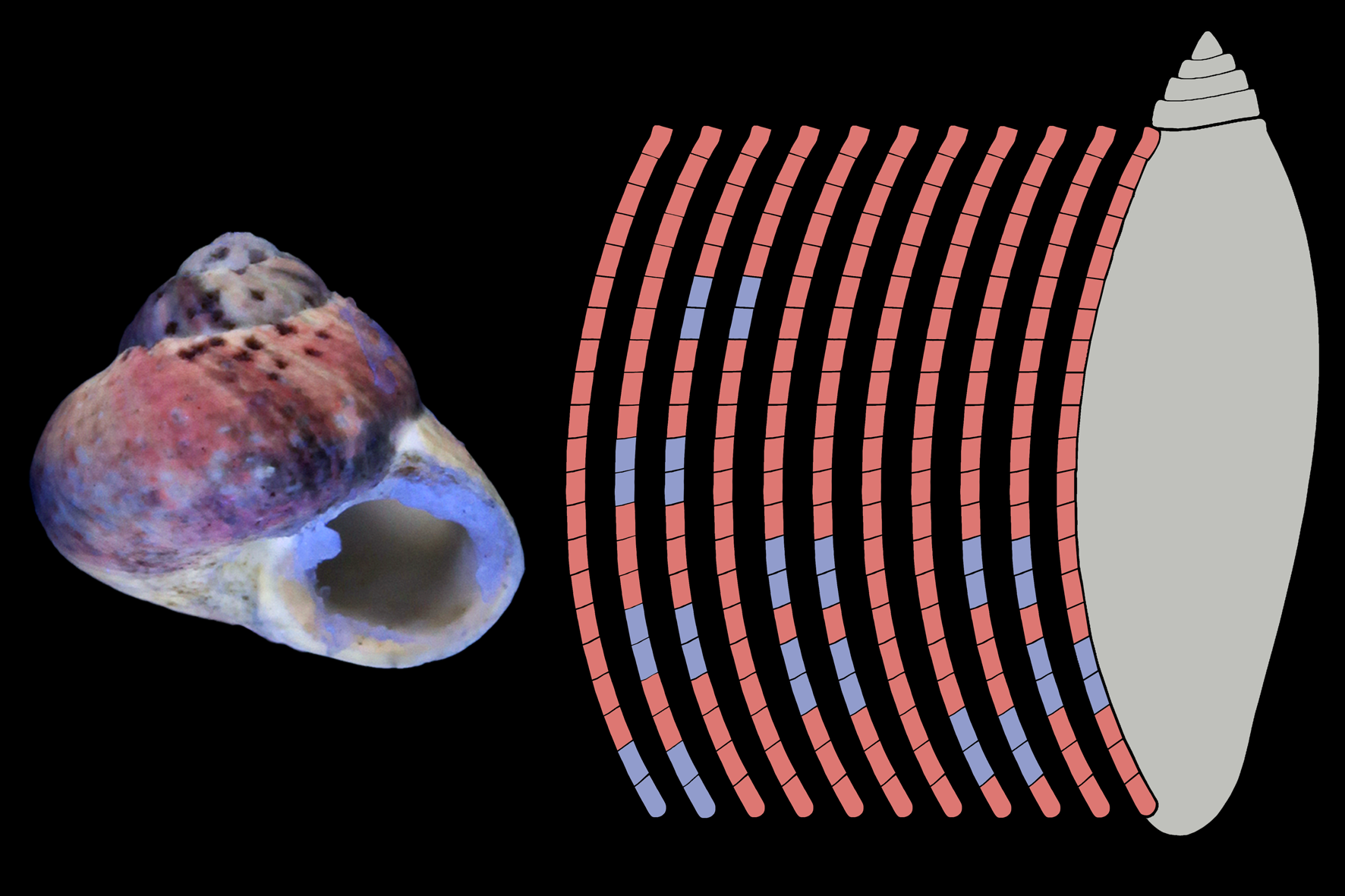

Supplement: S1 Fig — Pattern 8G: irregular dark patches contrasting with fluorescent colouration. (TIF) [file pone.0126745.s003.tif]

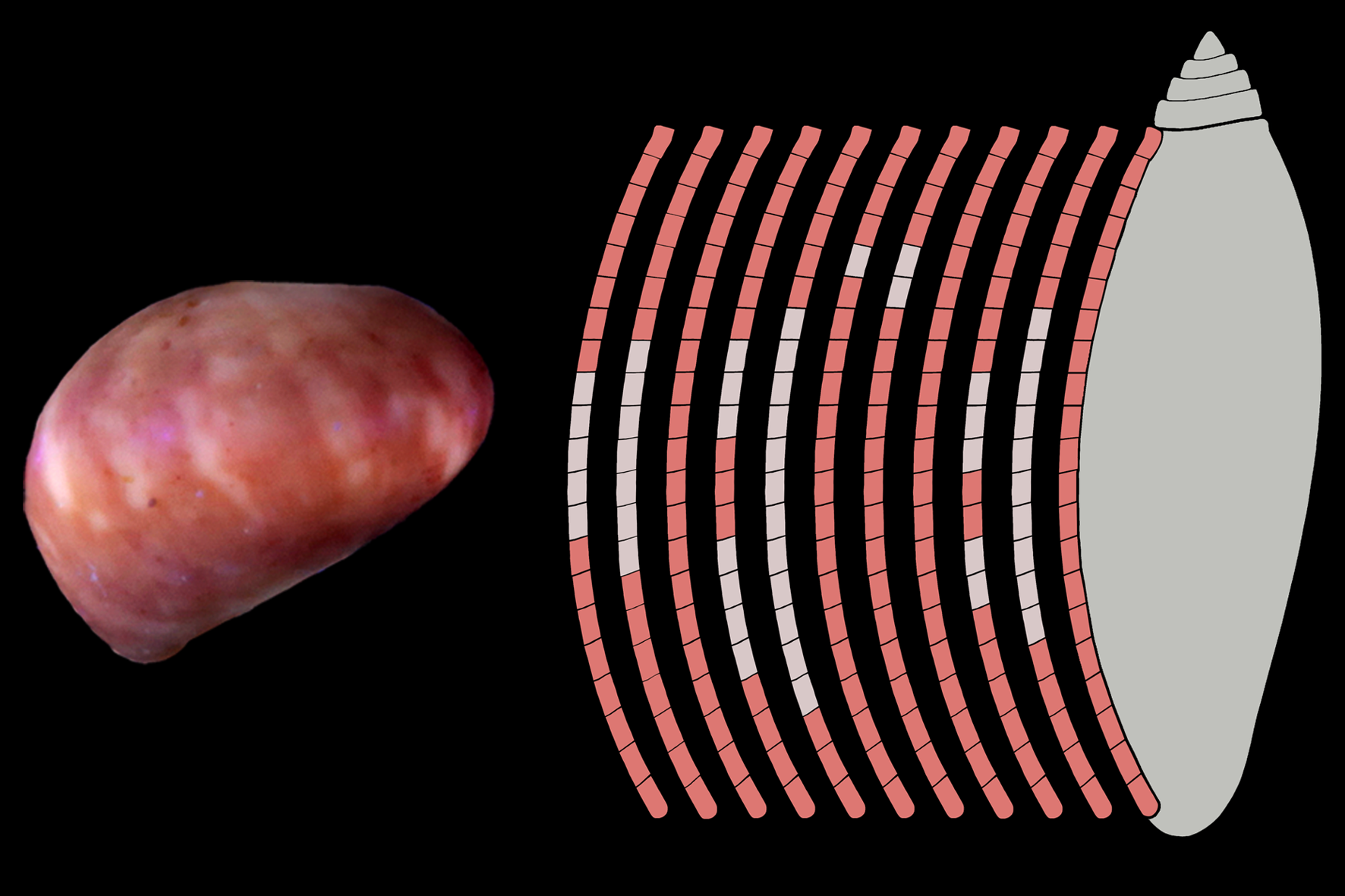

Supplement: S2 Fig — Pattern 8G: irregular dark patches contrasting with fluorescent colouration. (TIF) [file pone.0126745.s004.tif]

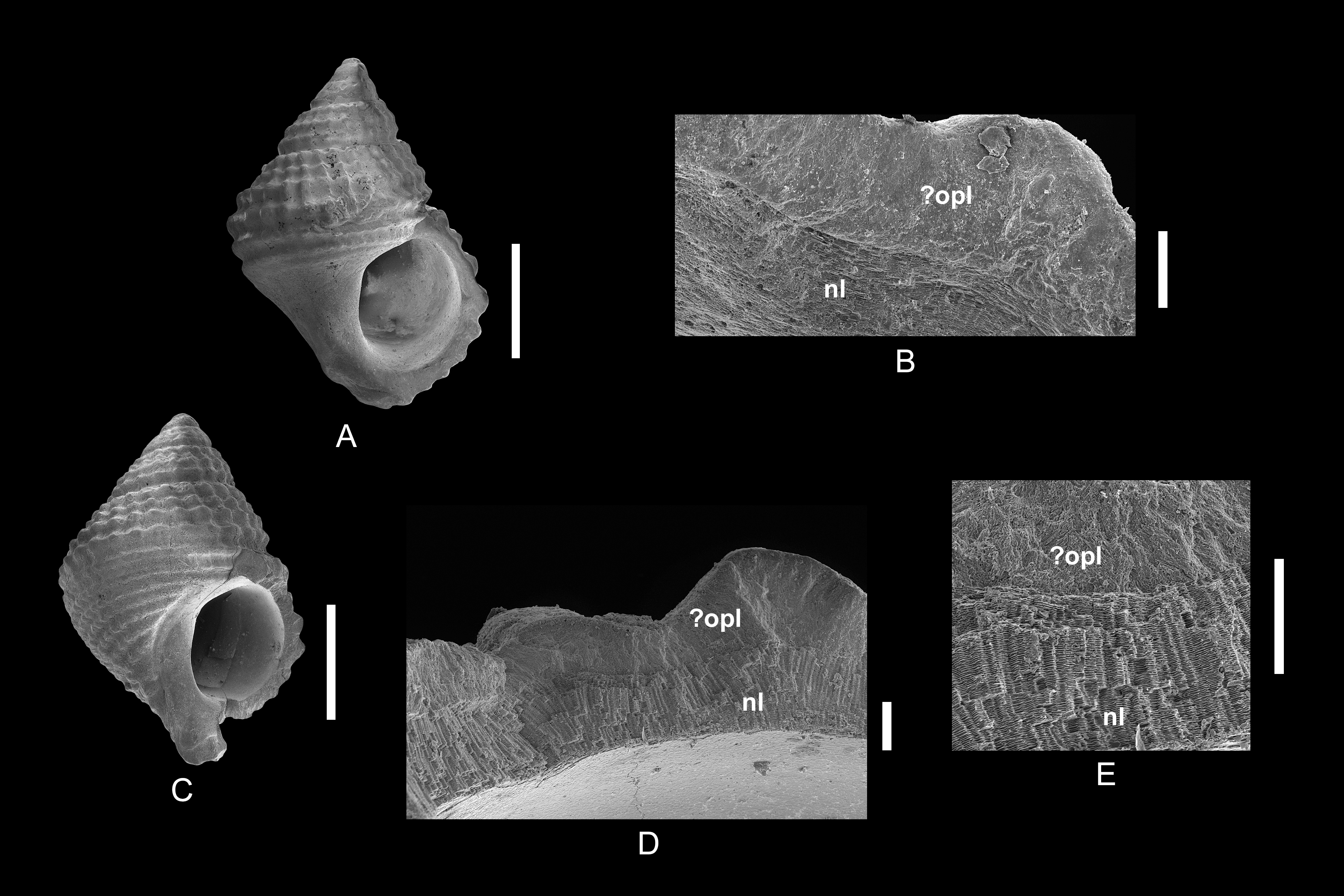

Supplement: S3 Fig — (A, B) UPMC-129 (Le Marchand coll.). (A) apertural view. (B) shell microstructure of the apertural margin. (C-E) UPMC-199 (Le Marchand coll.). (C) apertural view. (D) shell microstructure of the apertural margin. (E) detailed view of the photograph D. nl: nacreous layer.? opl: outer, probably prismatic layer. Scale bars: 2 mm (A, C), 100 μm (B, D, E). (TIF) [file pone.0126745.s005.tif]

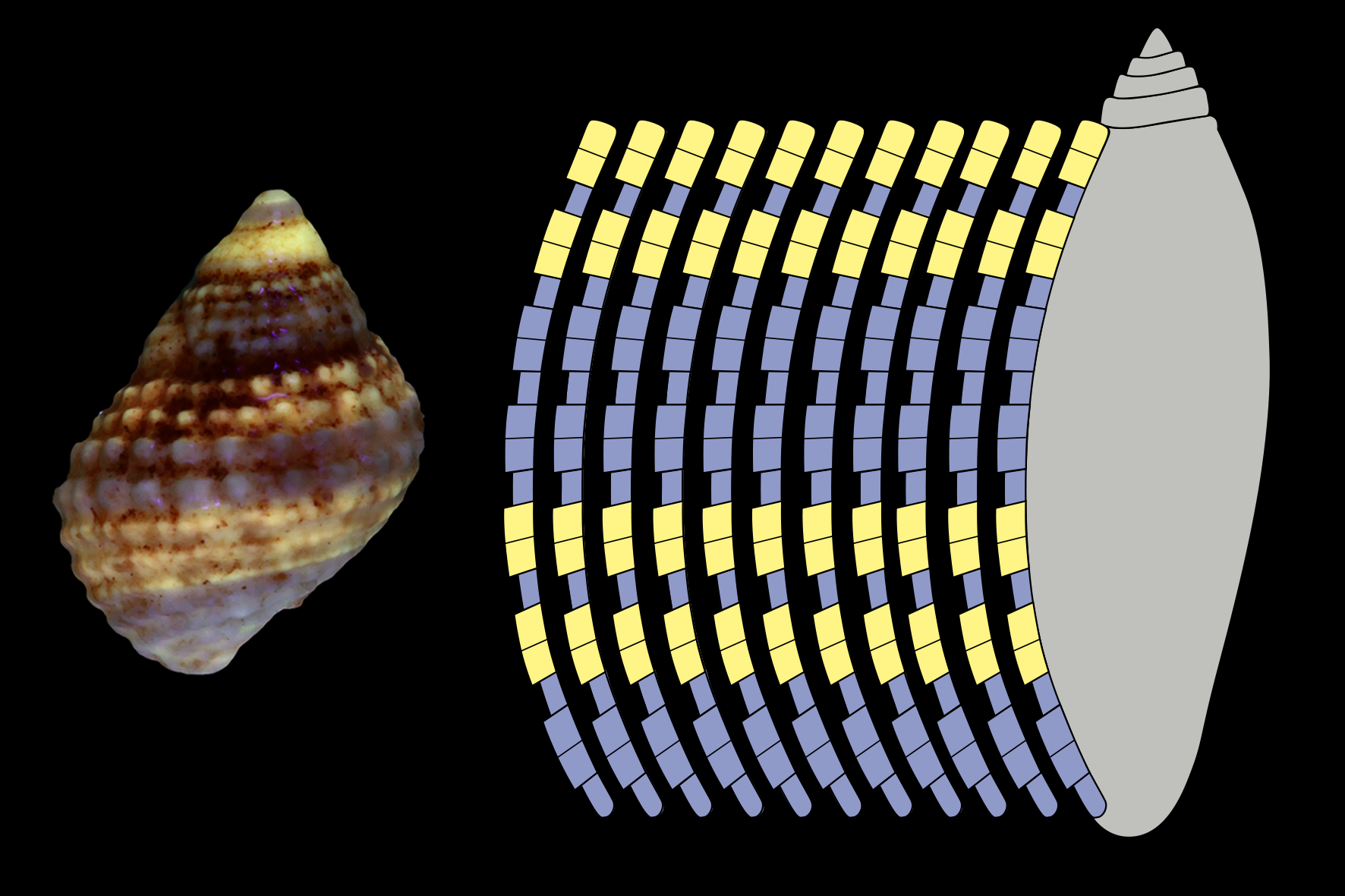

Supplement: S4 Fig — Pattern 2G: fluorescent, spiral stripes, located on spiral cords. (TIF) [file pone.0126745.s006.tif]

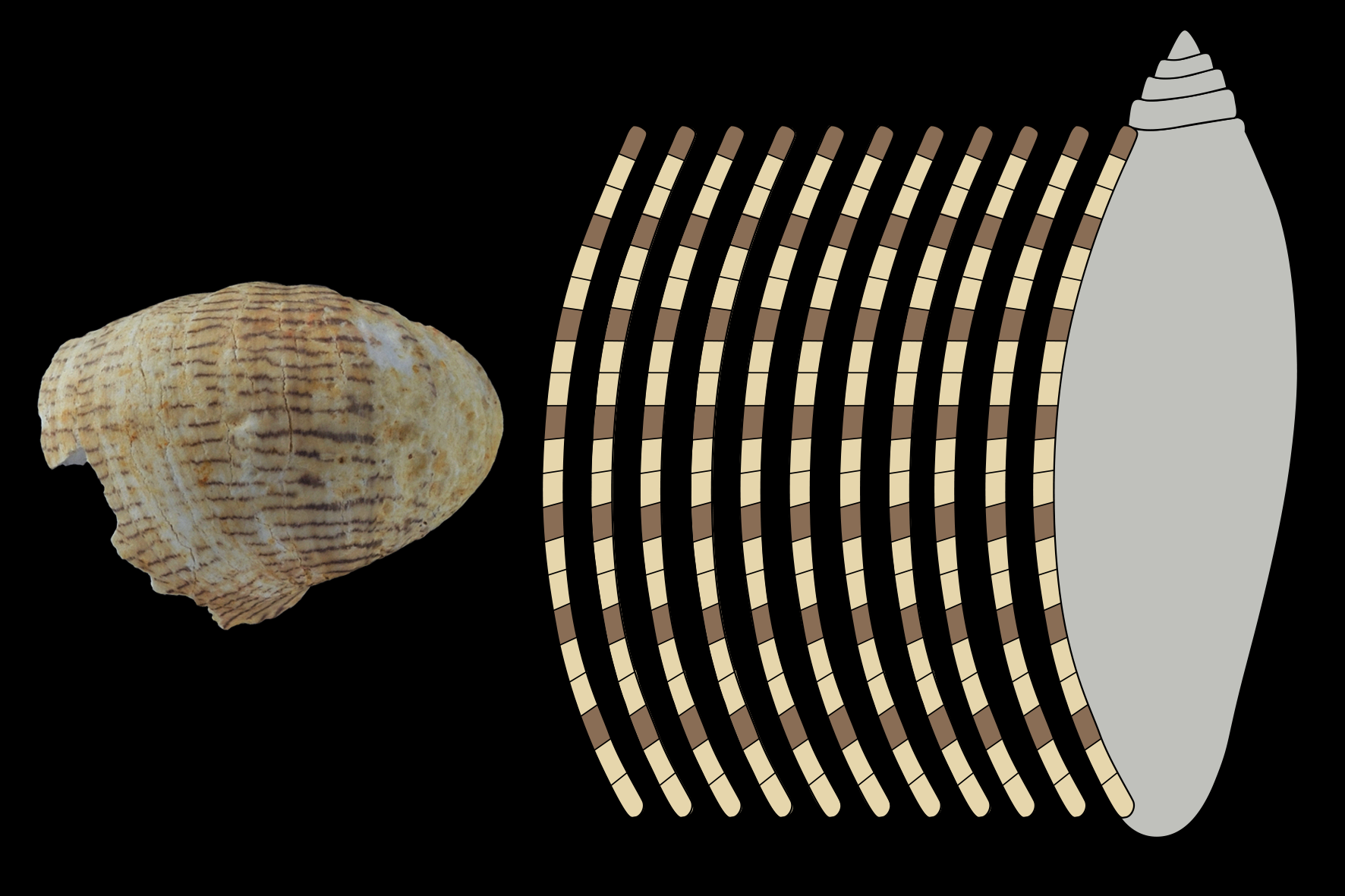

Supplement: S5 Fig — Pattern 3G (observed in natural light): spiral stripes. (TIF) [file pone.0126745.s007.tif]

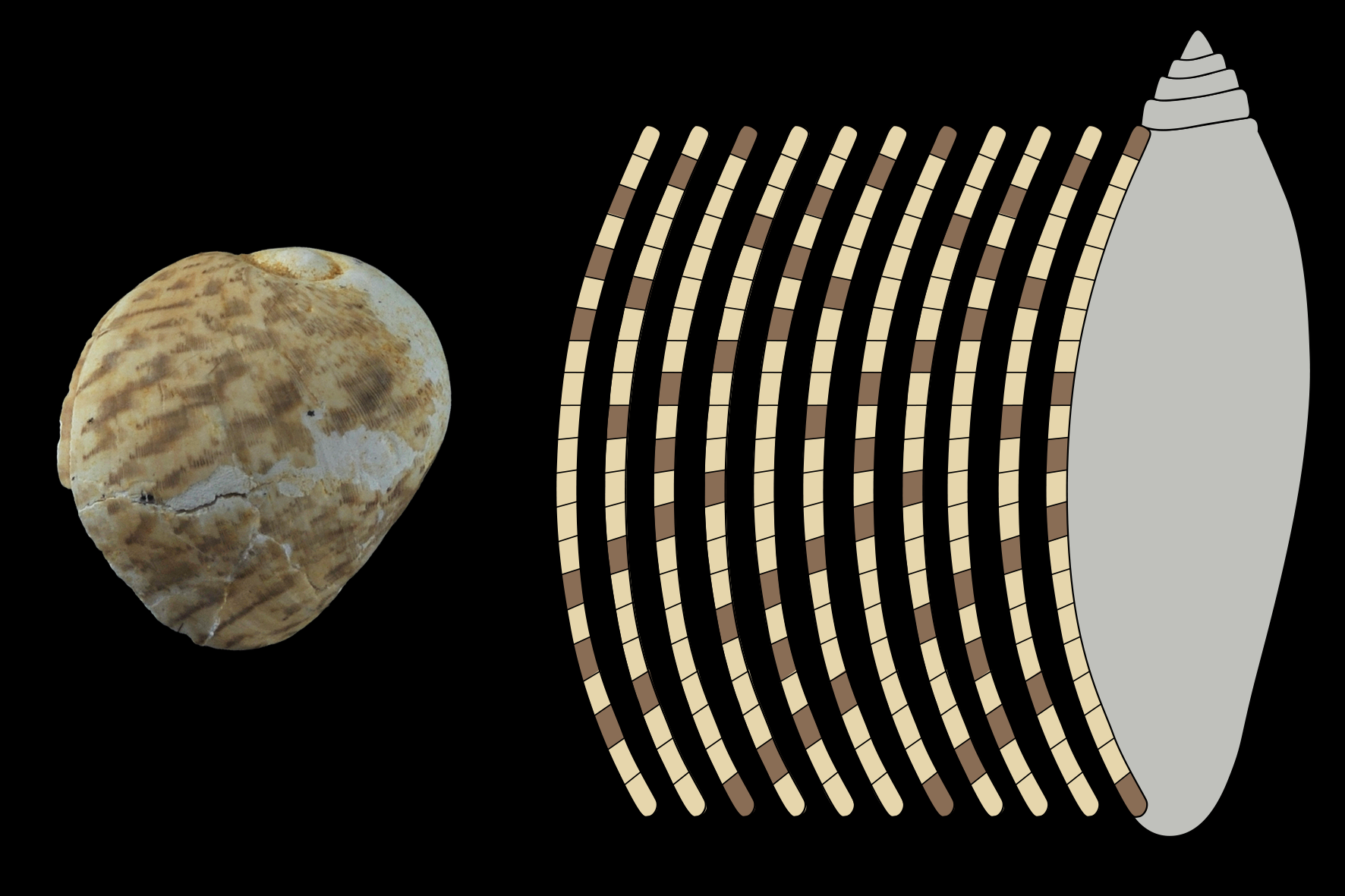

Supplement: S6 Fig — (TIF) [file pone.0126745.s008.tif]

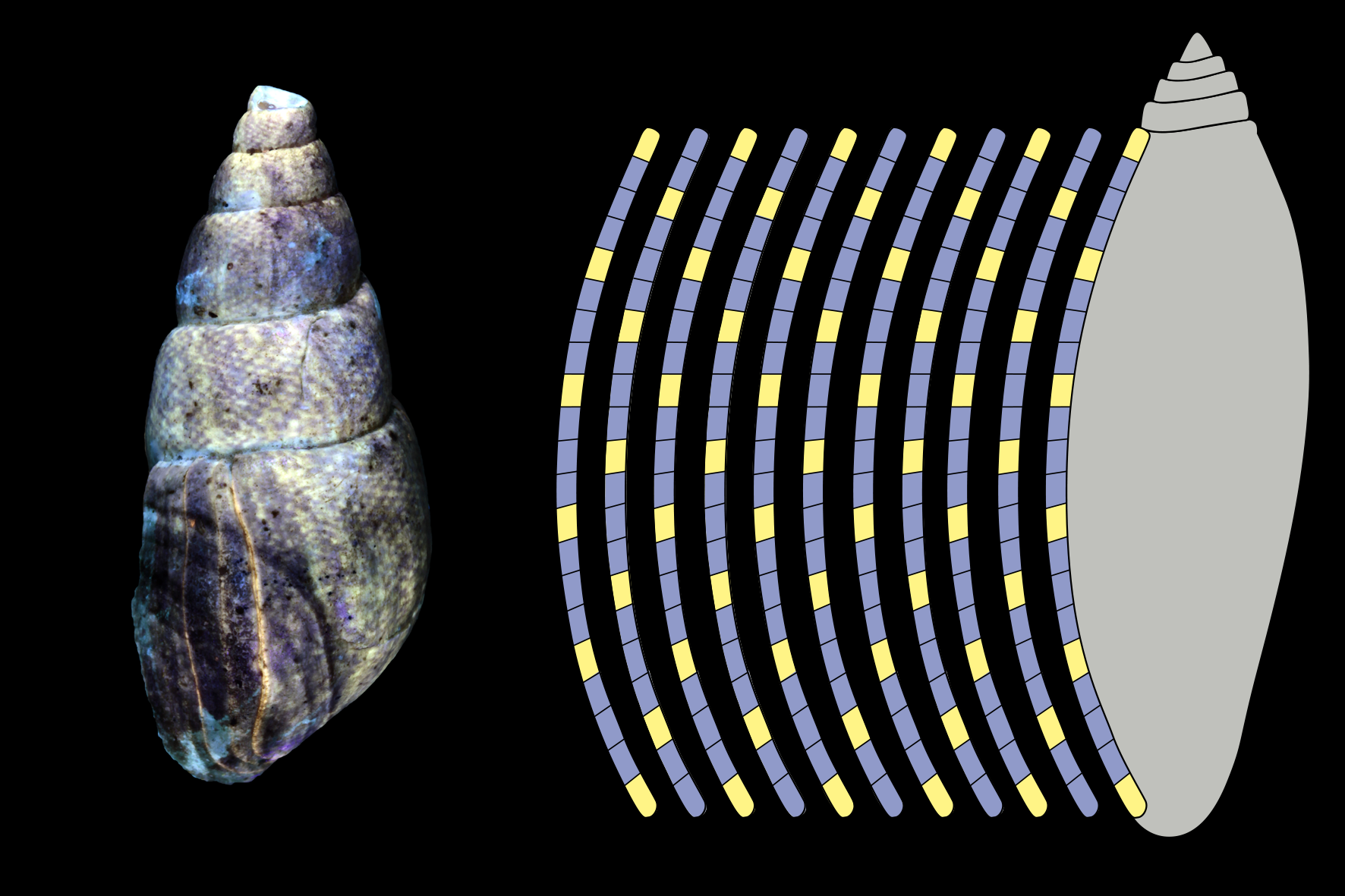

Supplement: S7 Fig — Pattern 4G: meshwork. (TIF) [file pone.0126745.s009.tif]

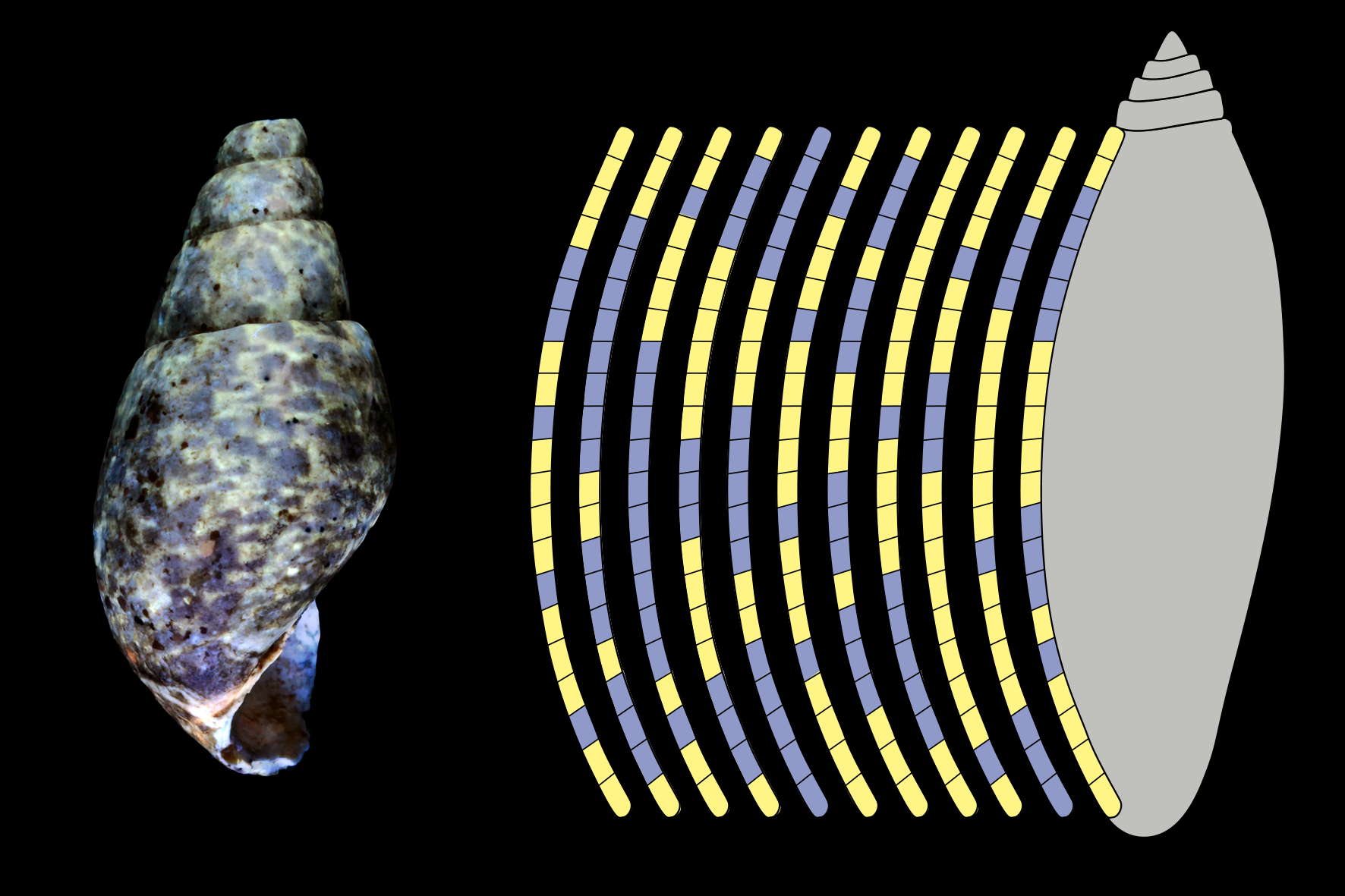

Supplement: S8 Fig — Pattern 1G: triangular, dark false patches, contrasting with fluorescent colouration. (TIF) [file pone.0126745.s010.tif]

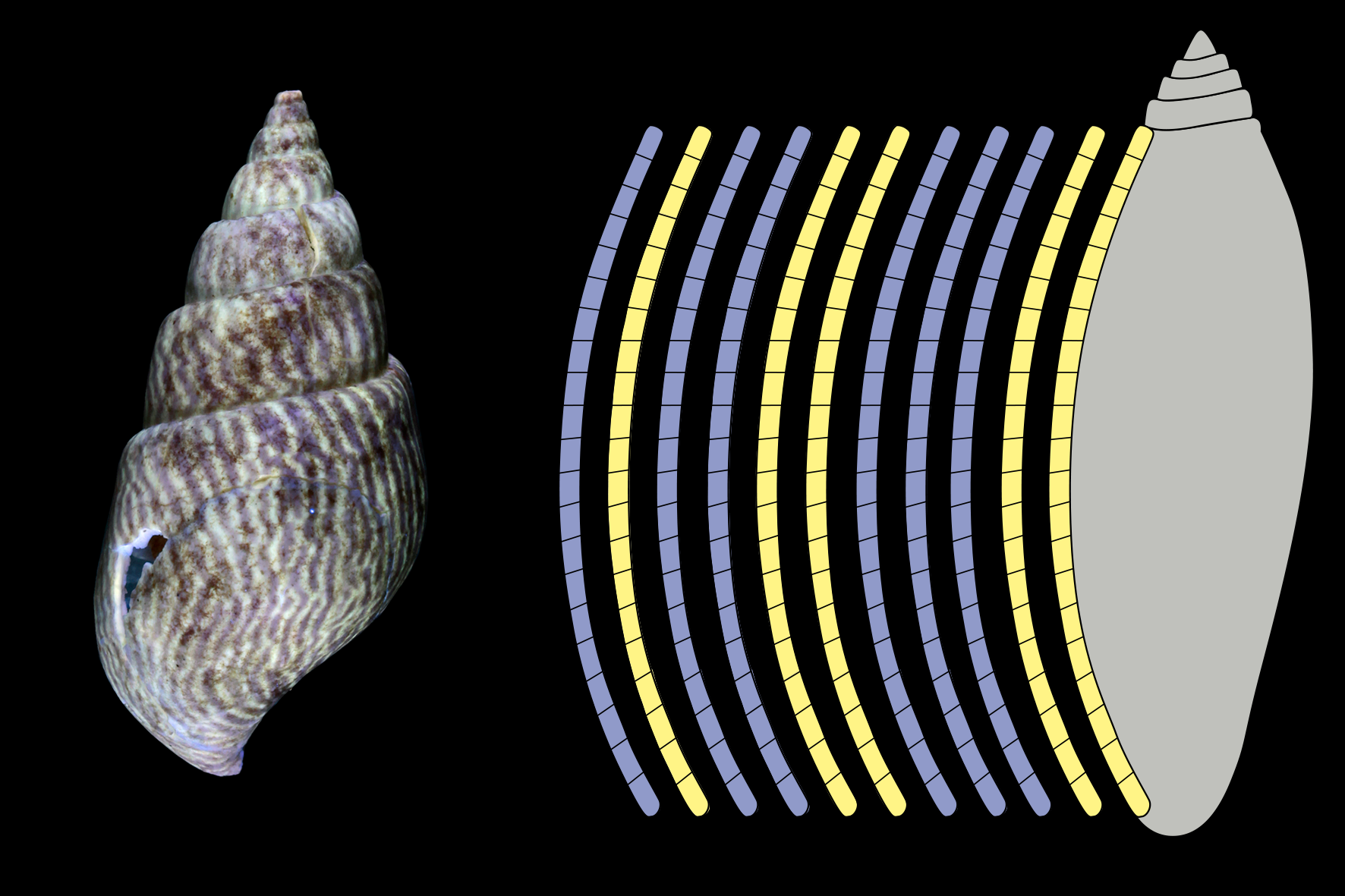

Supplement: S9 Fig — Pattern 5G: fluorescent axial stripes. (TIF) [file pone.0126745.s011.tif]

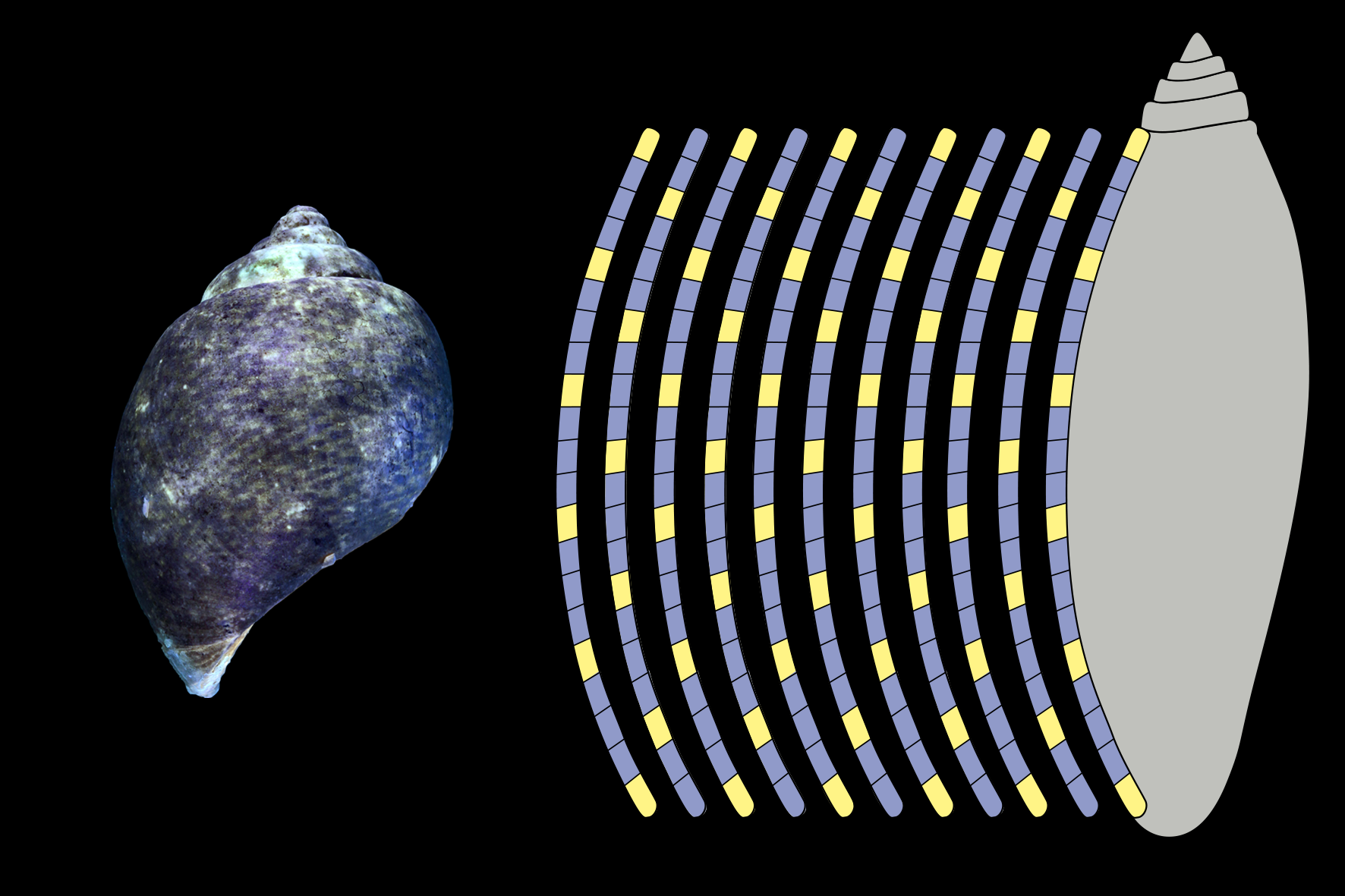

Supplement: S10 Fig — Pattern 4G: meshwork. (TIF) [file pone.0126745.s012.tif]

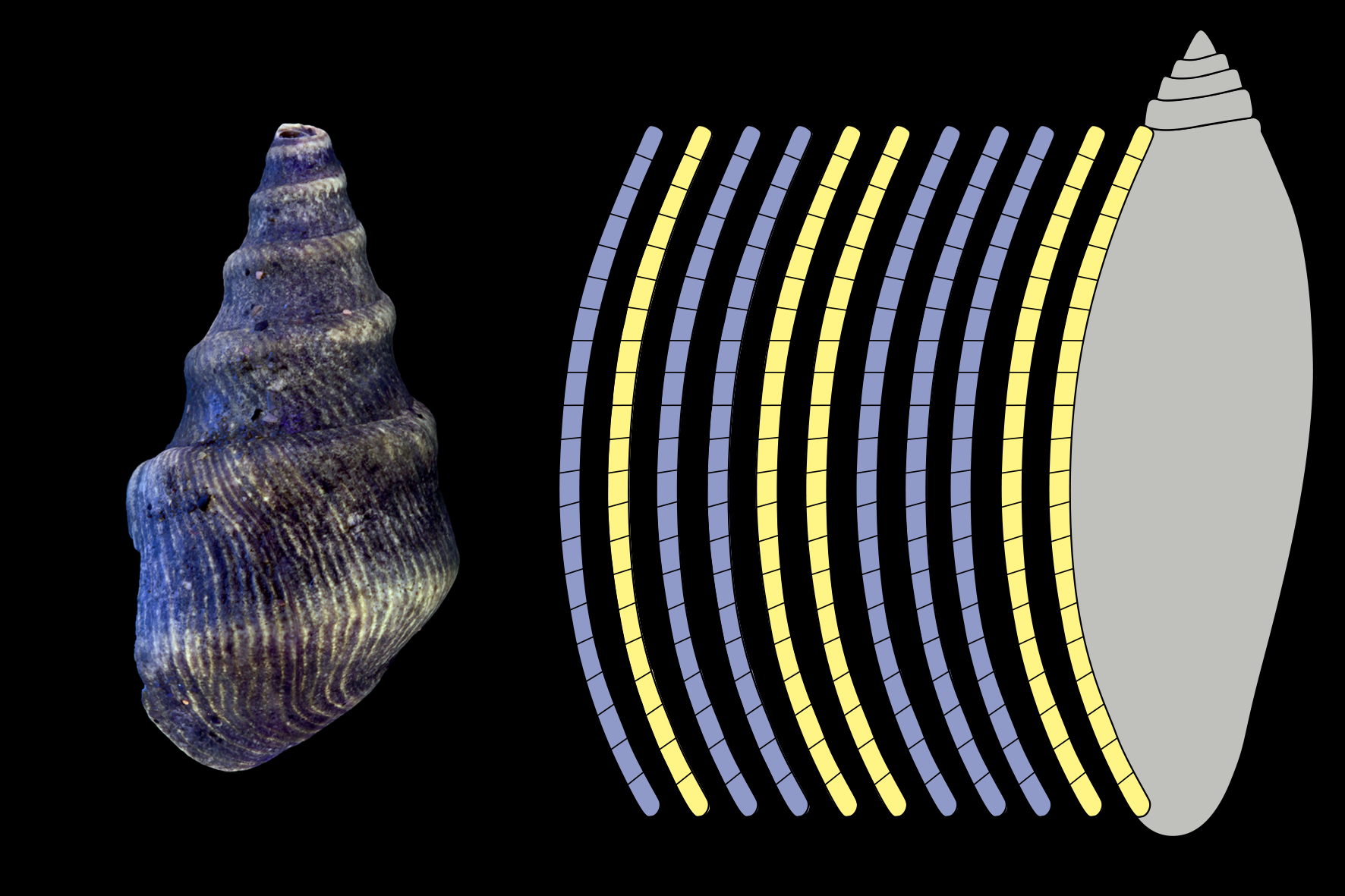

Supplement: S11 Fig — Pattern 5G: fluorescent axial stripes. (TIF) [file pone.0126745.s013.tif]

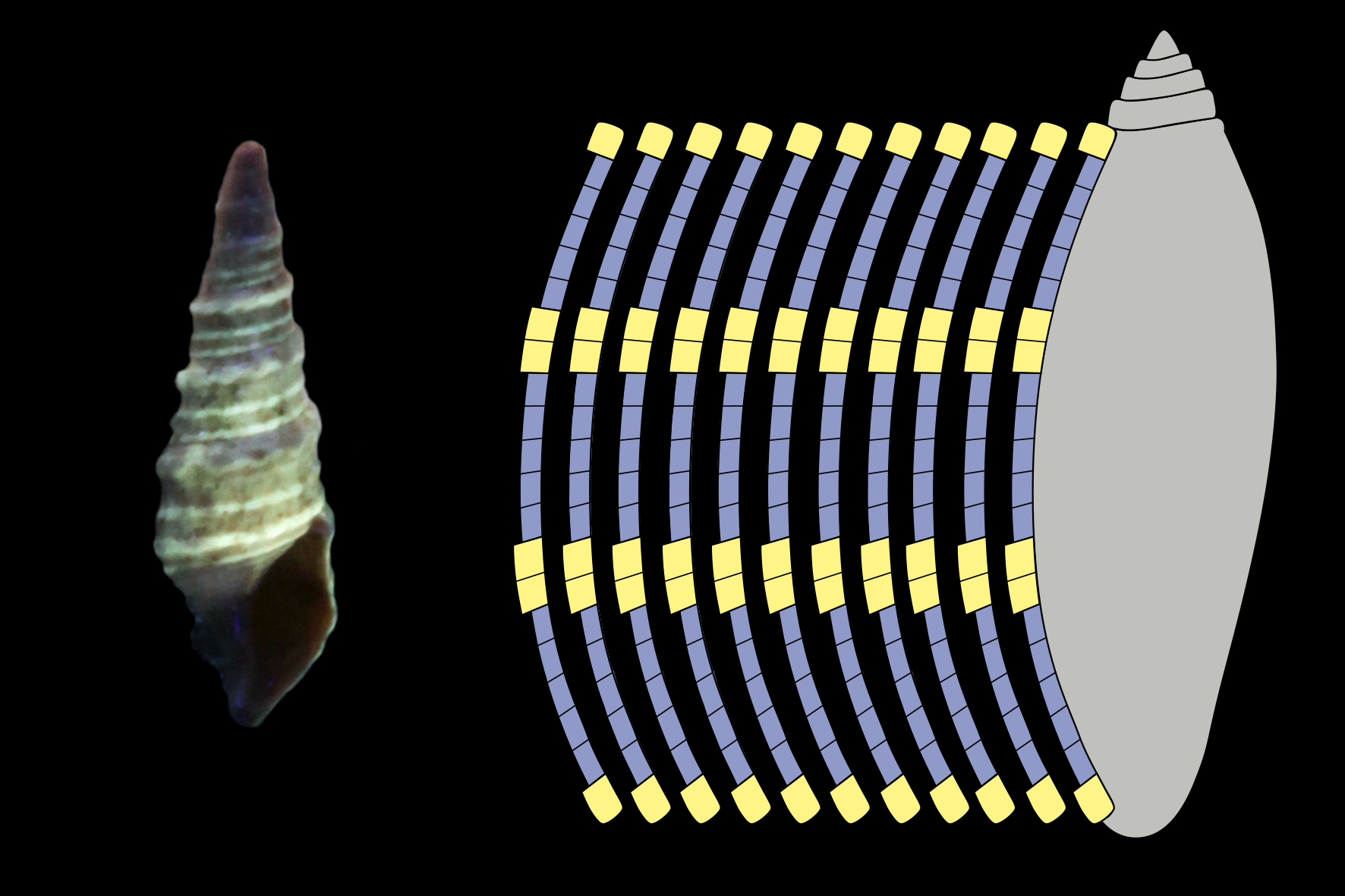

Supplement: S12 Fig — Pattern 2G: fluorescent, spiral stripes, located on spiral cords. (TIF) [file pone.0126745.s014.tif]

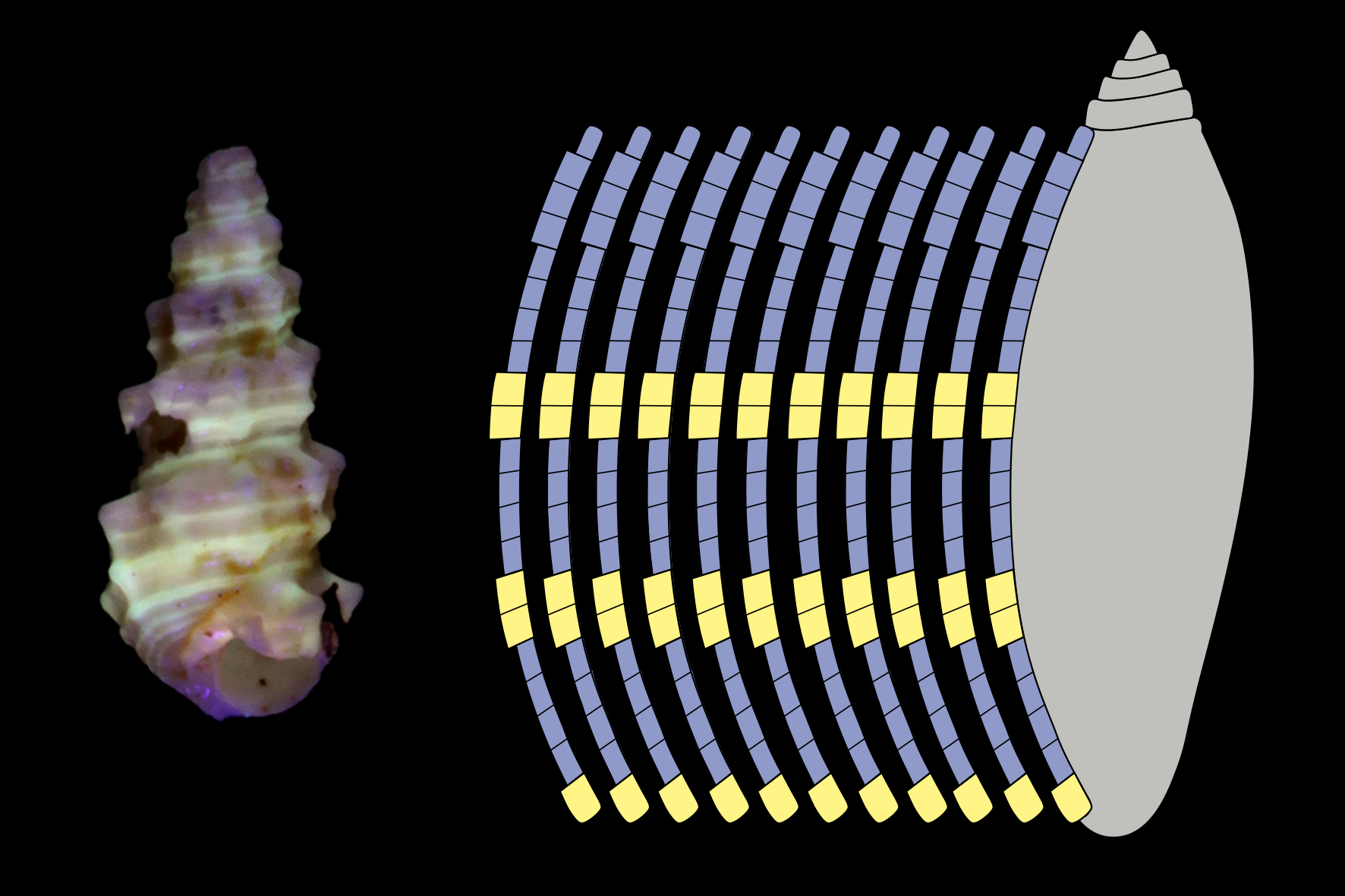

Supplement: S13 Fig — Pattern 2G: fluorescent, spiral stripes, located on spiral cords. (TIF) [file pone.0126745.s015.tif]

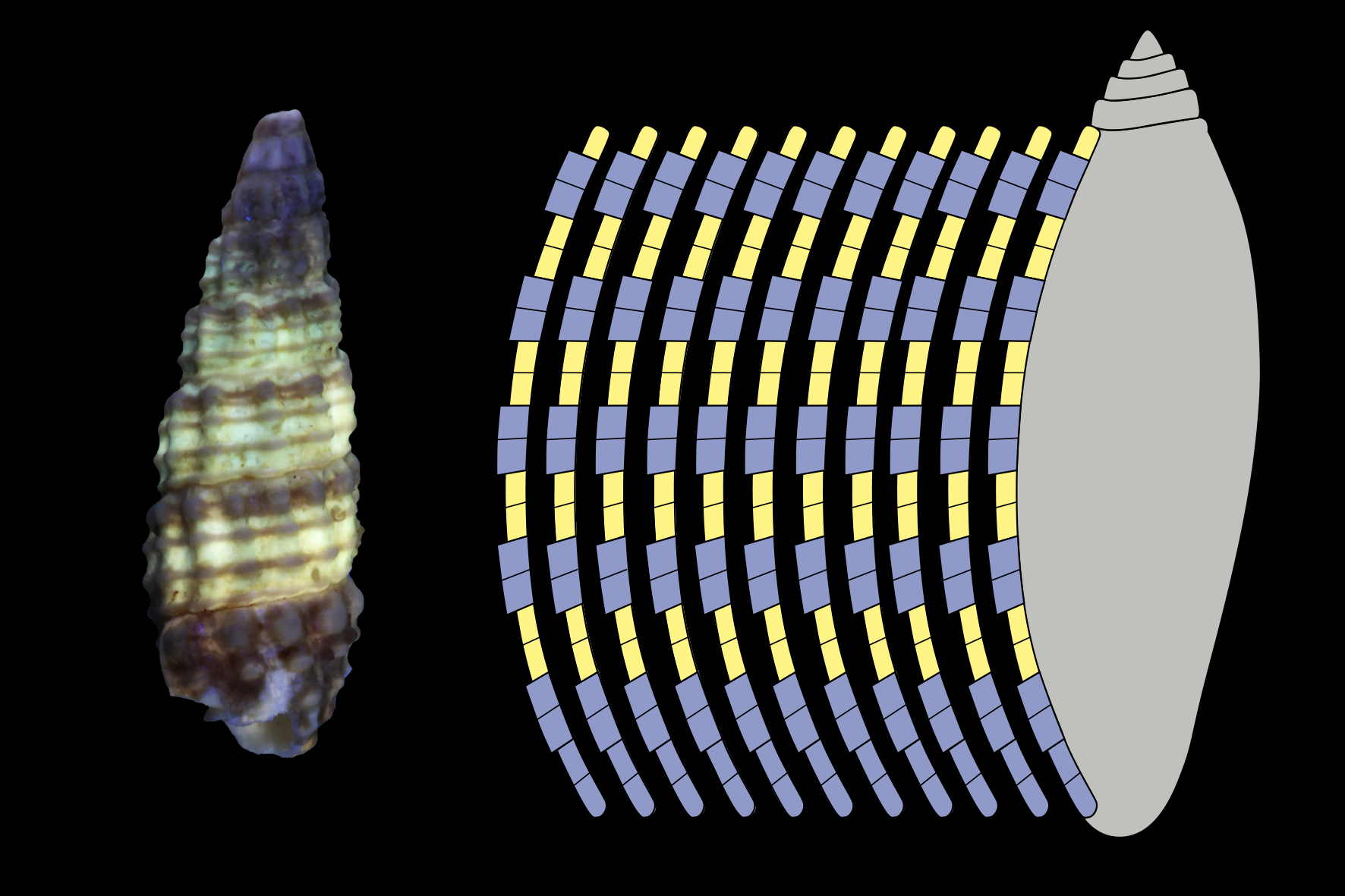

Supplement: S14 Fig — Pattern 6G: fluorescent spiral stripes located between the spiral cords or uniformly fluorescent whorls. (TIF) [file pone.0126745.s016.tif]

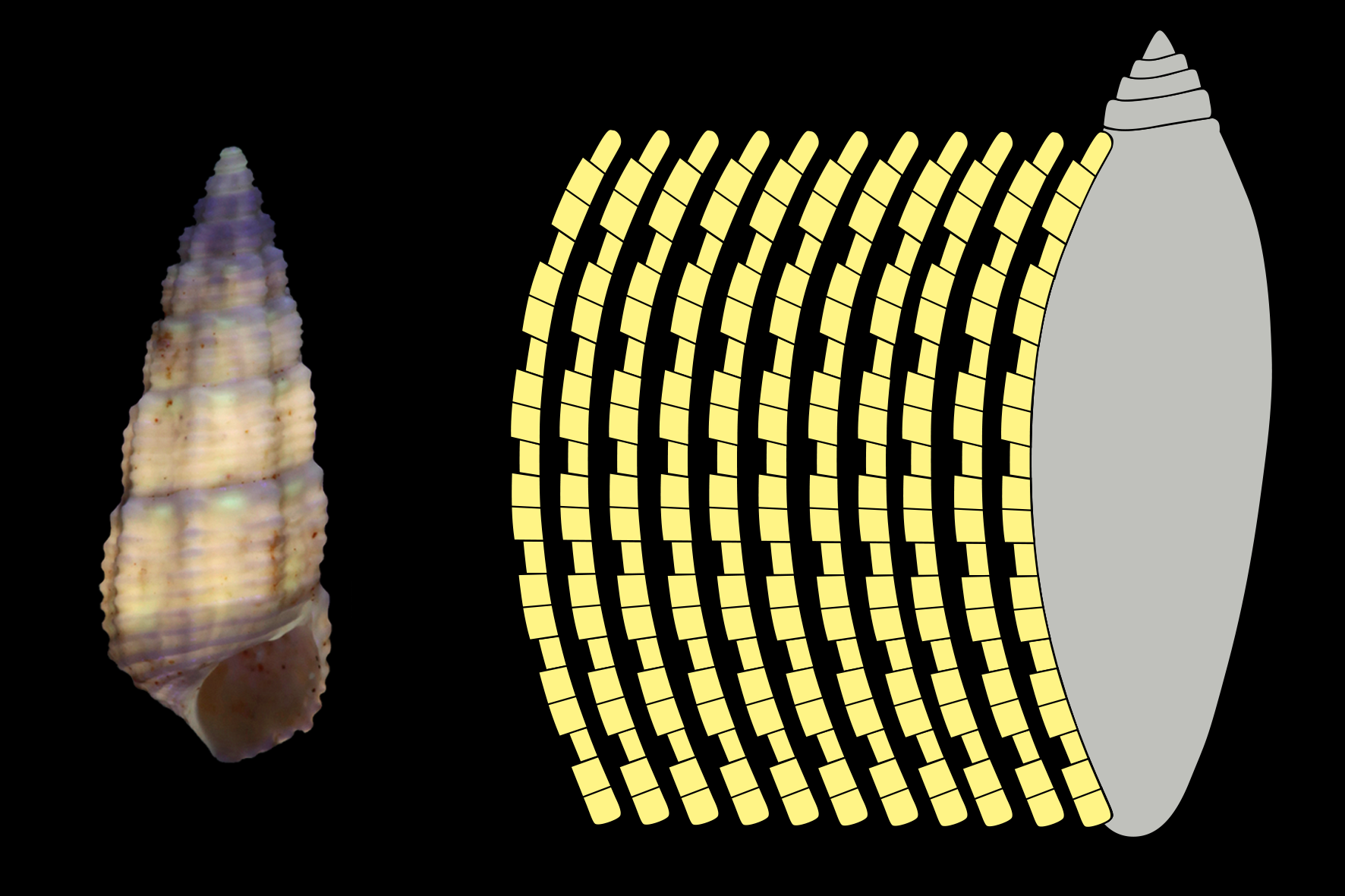

Supplement: S15 Fig — Pattern 6G: fluorescent spiral stripes located between the spiral cords or uniformly fluorescent whorls. (TIF) [file pone.0126745.s017.tif]

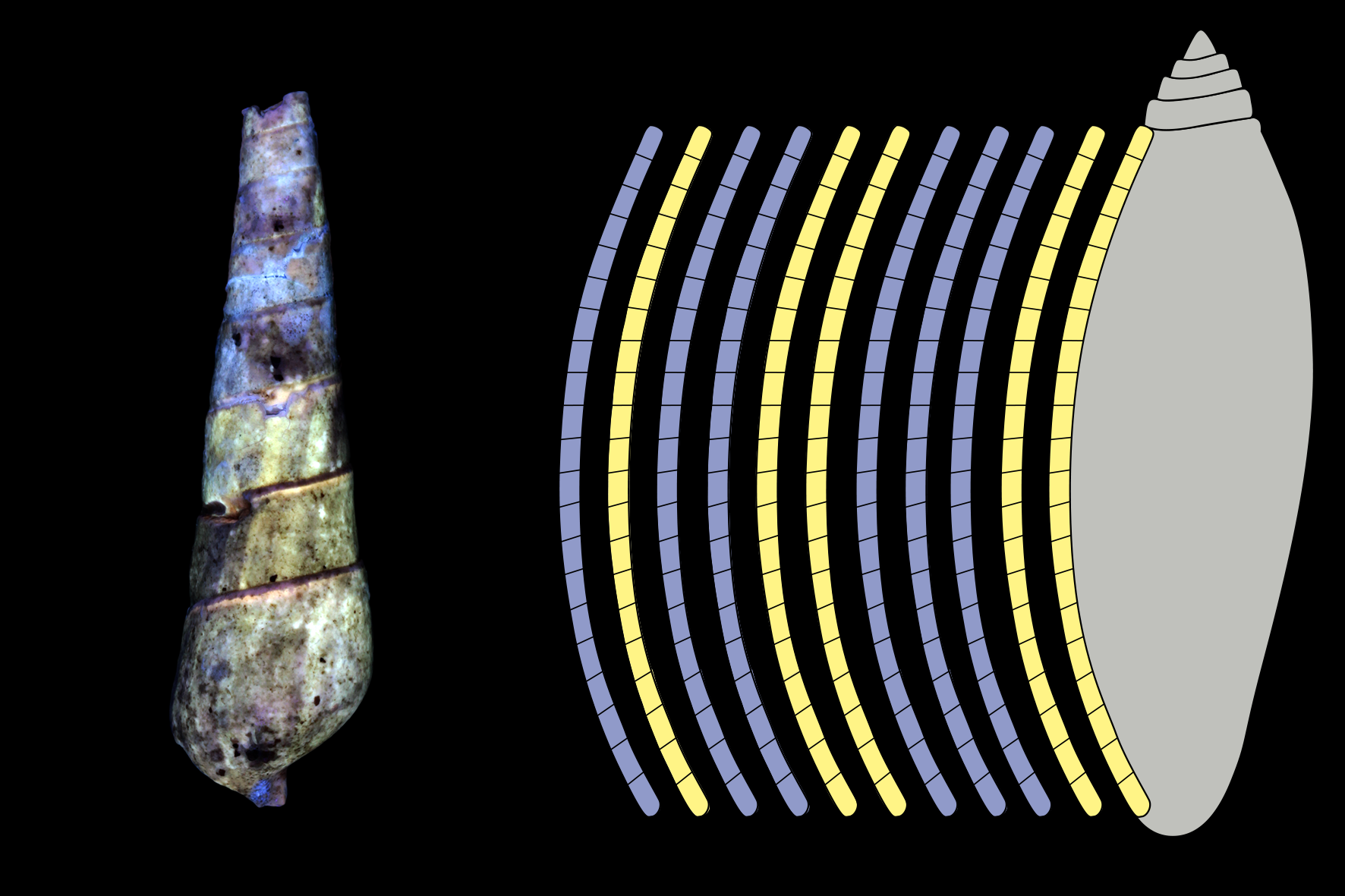

Supplement: S16 Fig — Pattern 5G: fluorescent axial stripes. (TIF) [file pone.0126745.s018.tif]

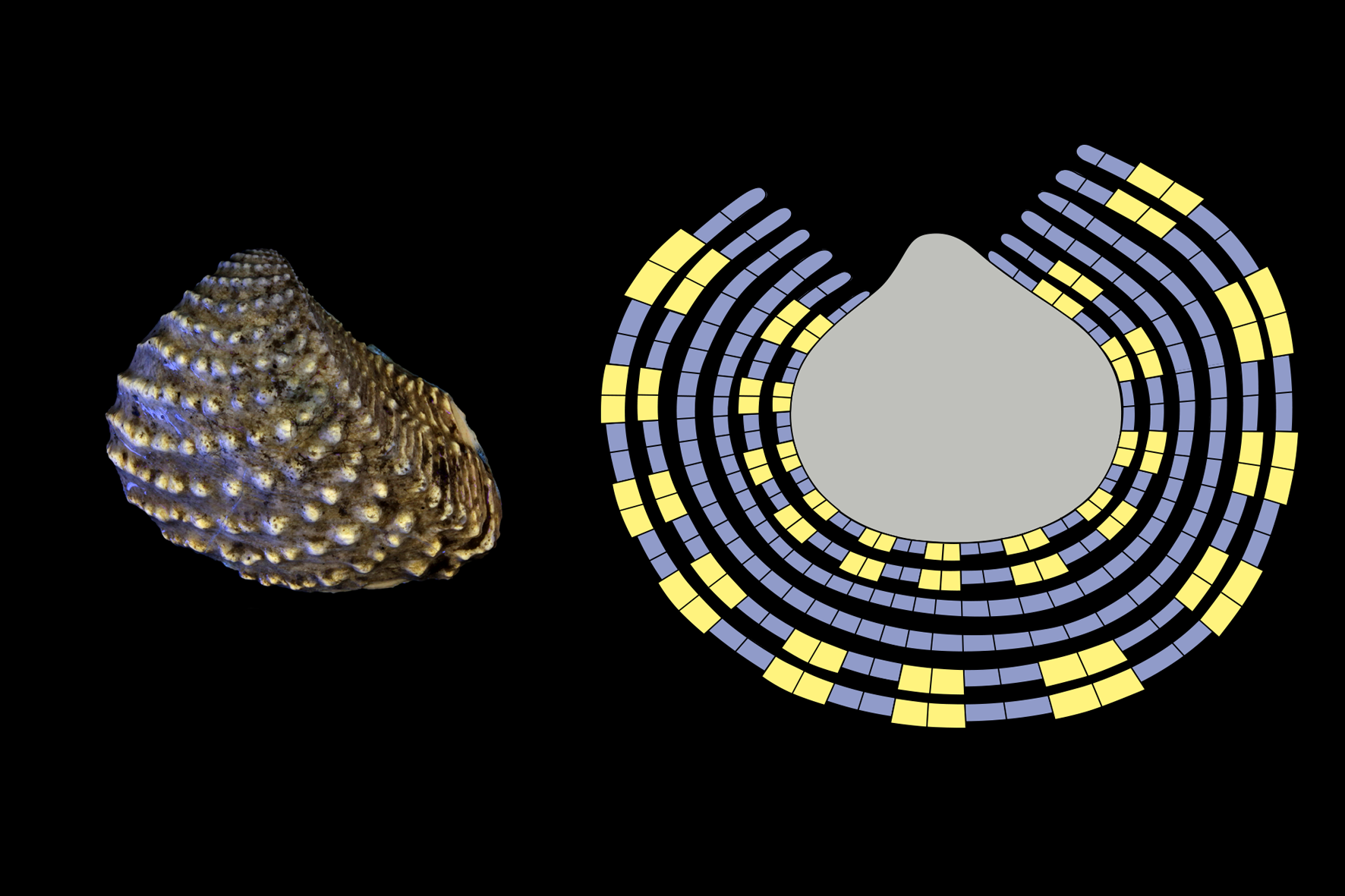

Supplement: S17 Fig — Pattern 3B: subcommarginal or oblique rows of fluorescent patches that are located on shell tubercles. (TIF) [file pone.0126745.s019.tif]

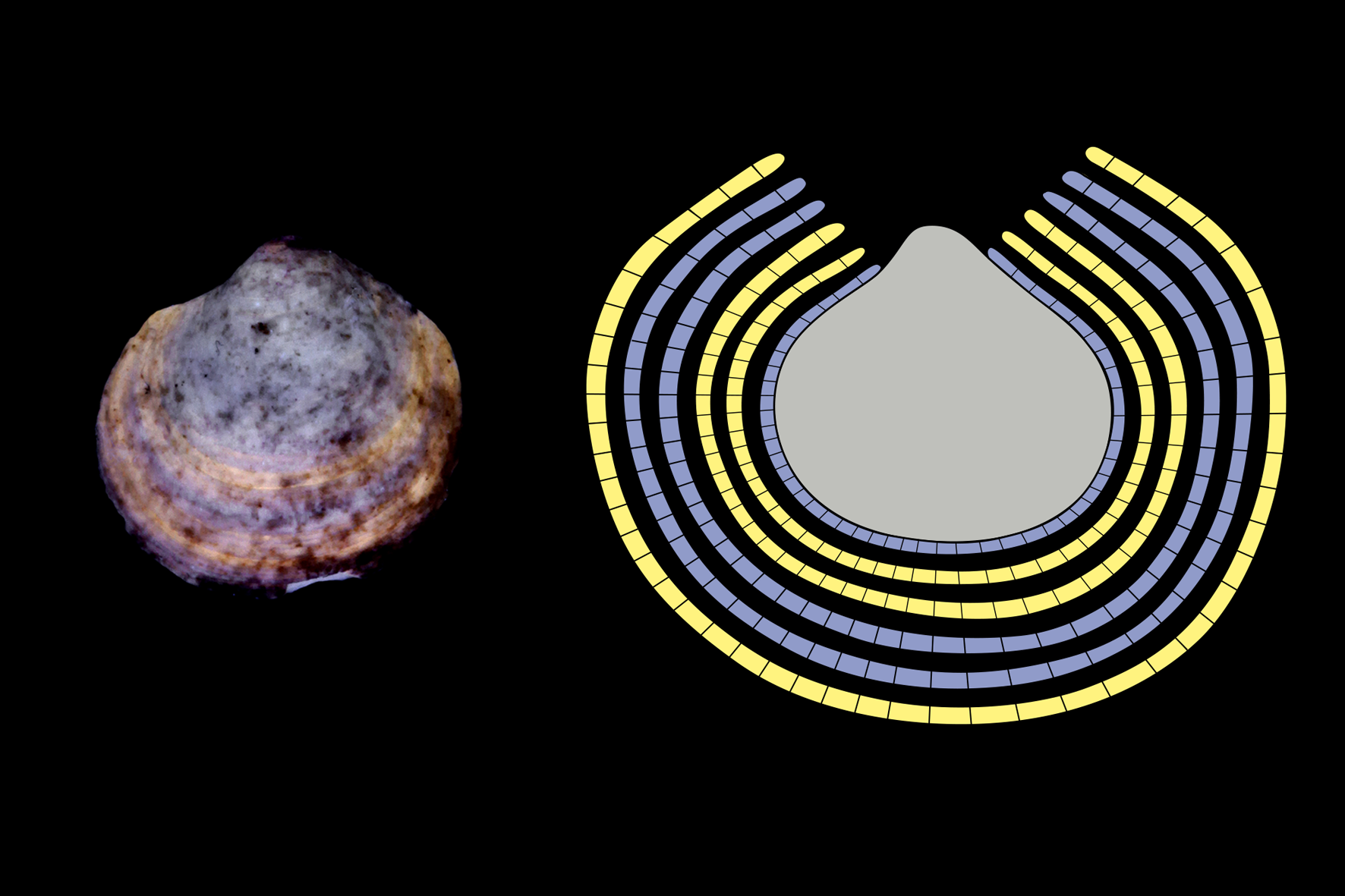

Supplement: S18 Fig — Pattern 1B: fluorescent commarginal stripes. (TIF) [file pone.0126745.s020.tif]

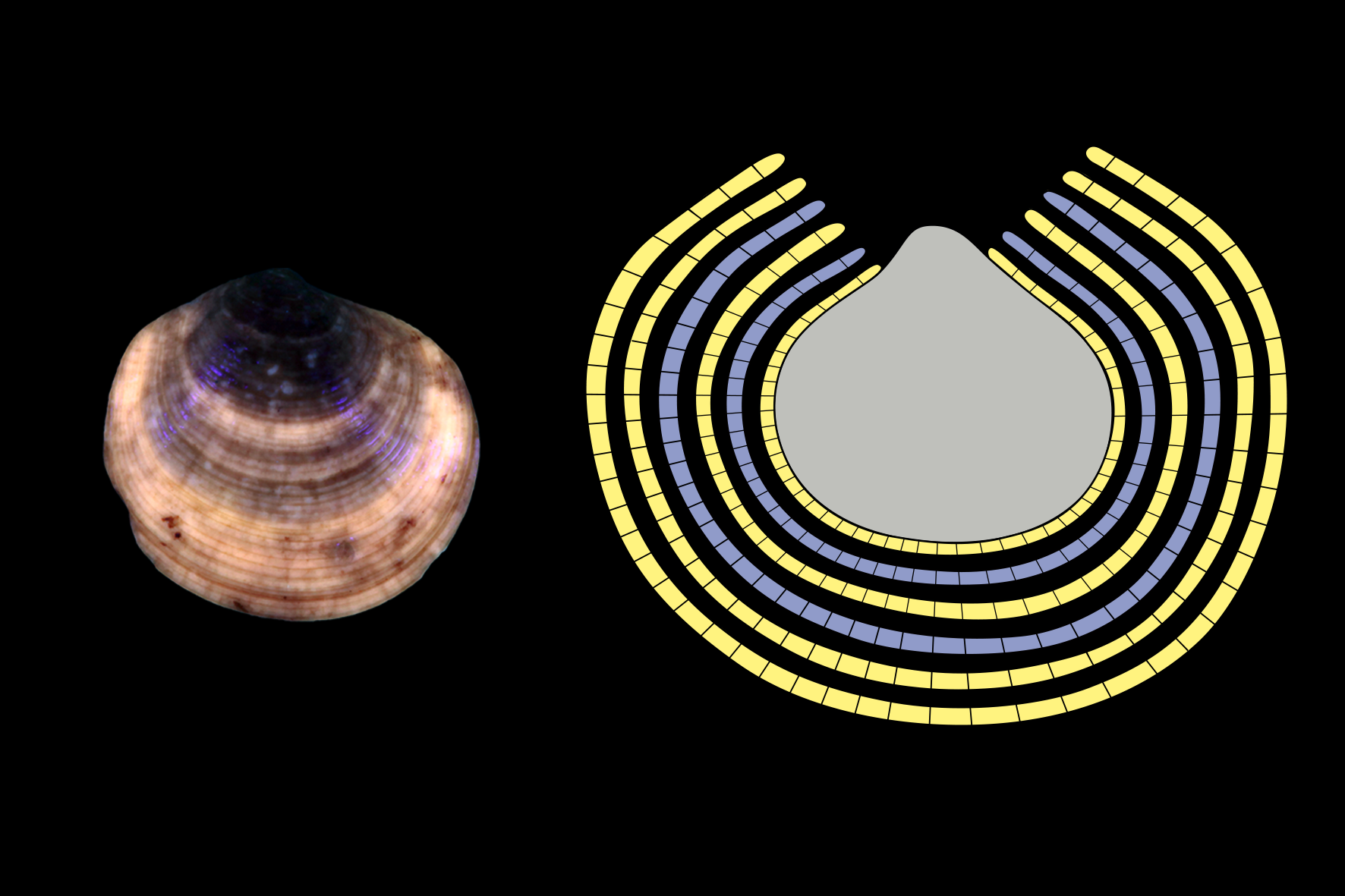

Supplement: S19 Fig — Pattern 1B: fluorescent commarginal stripes. (TIF) [file pone.0126745.s021.tif]

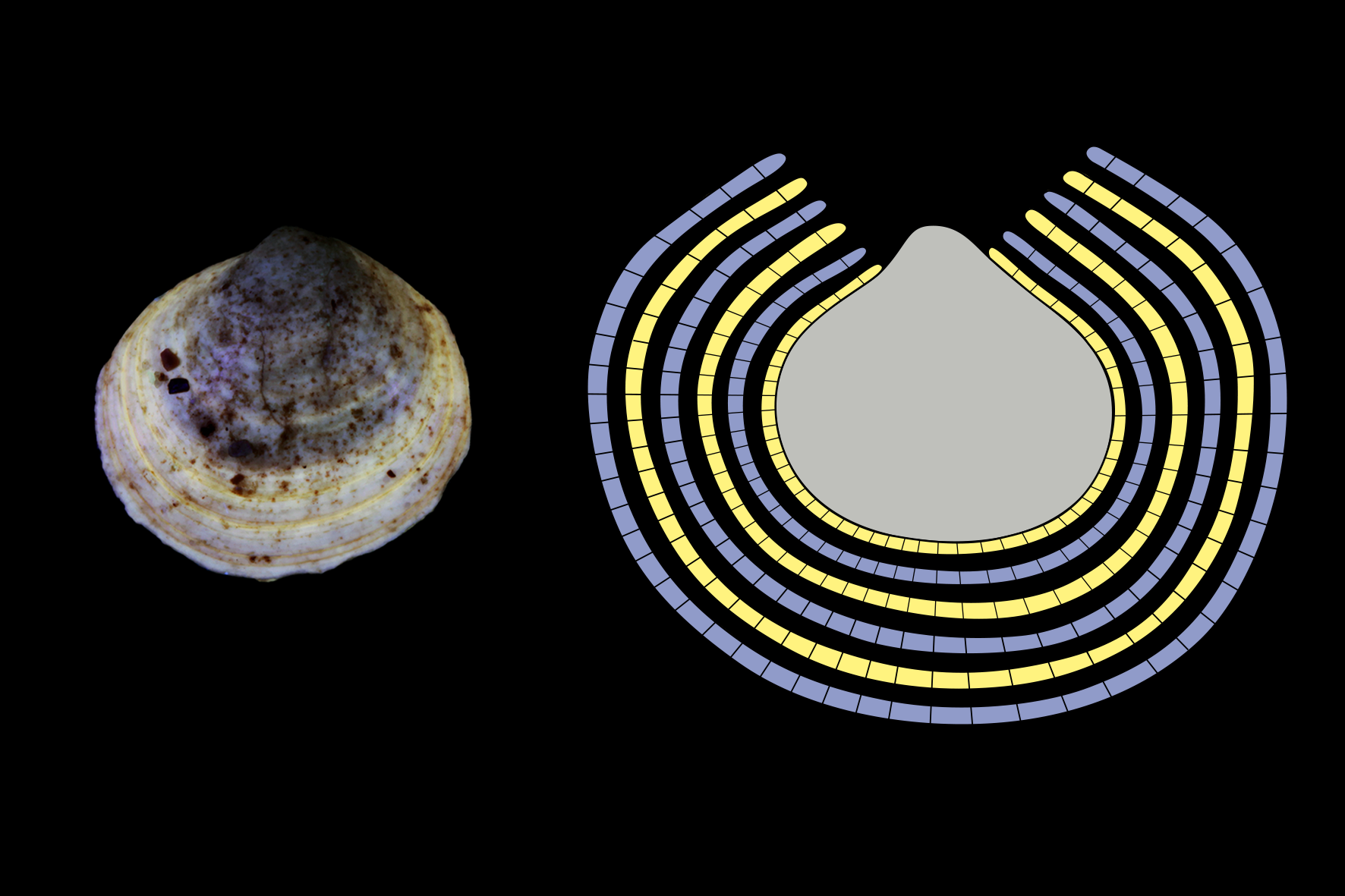

Supplement: S20 Fig — Pattern 1B: fluorescent commarginal stripes. (TIF) [file pone.0126745.s022.tif]

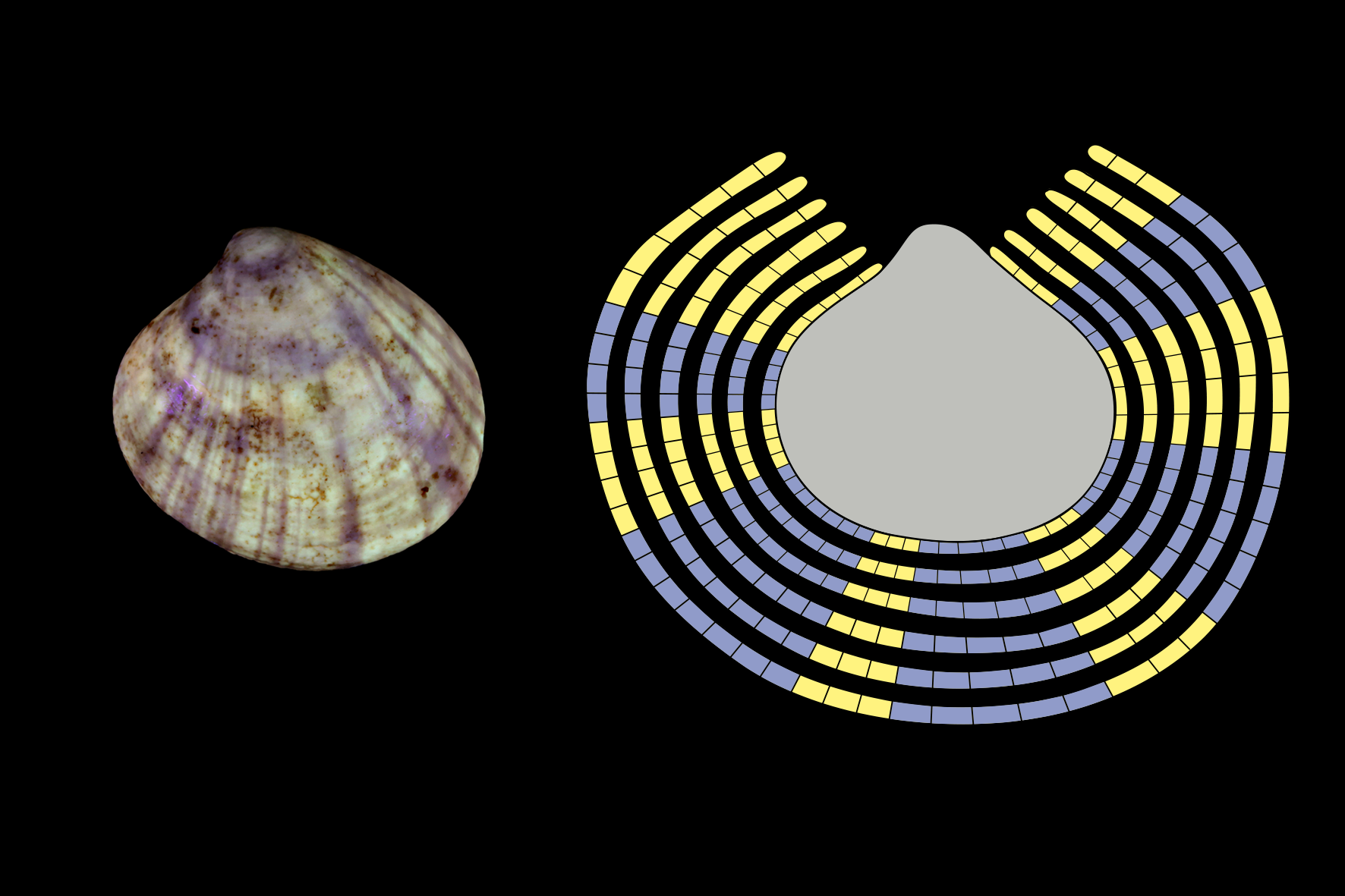

Supplement: S21 Fig — Pattern 2B: fluorescent radial stripes. (TIF) [file pone.0126745.s023.tif]

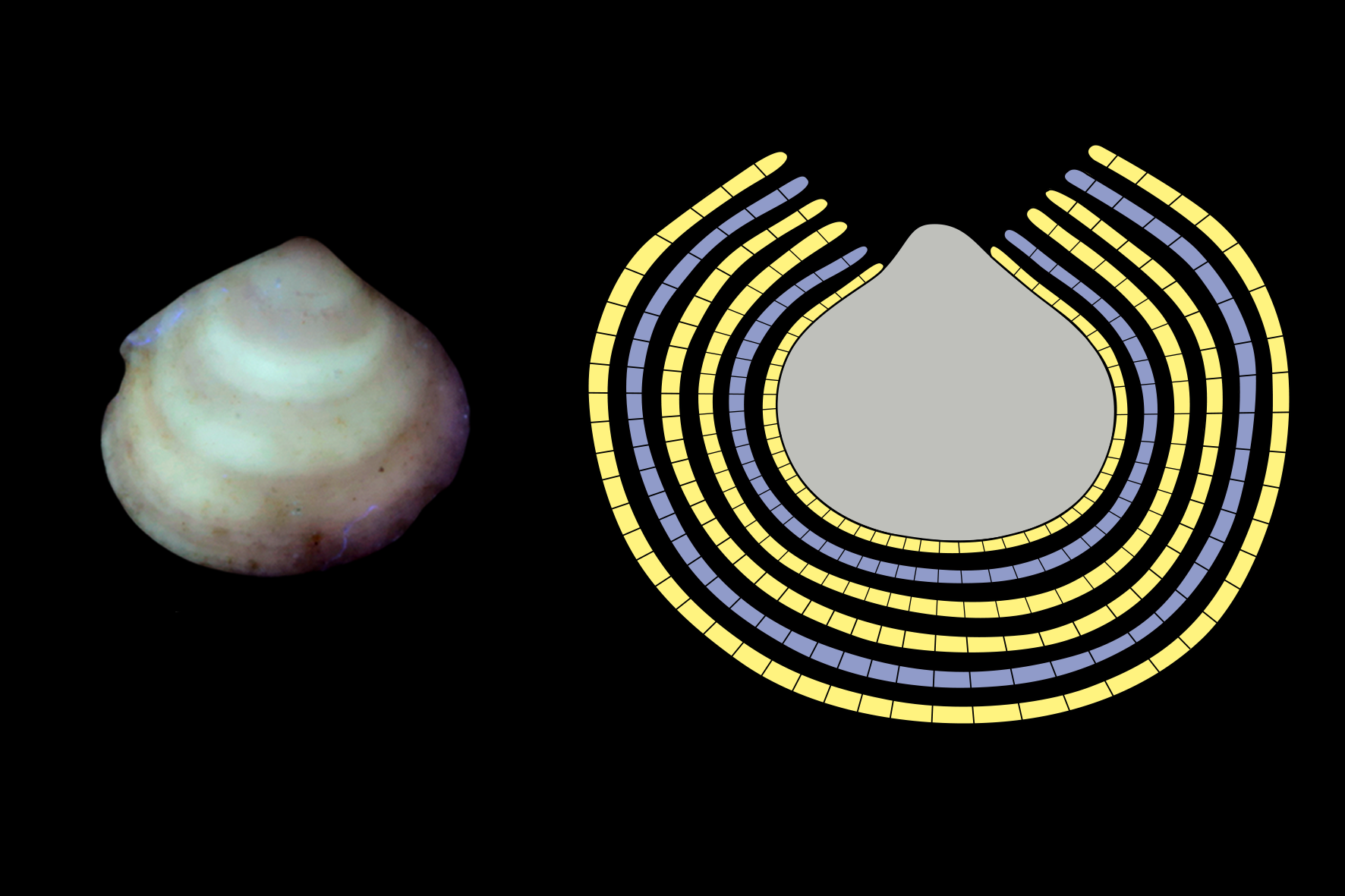

Supplement: S22 Fig — Pattern 1B: fluorescent commarginal stripes. (TIF) [file pone.0126745.s024.tif]

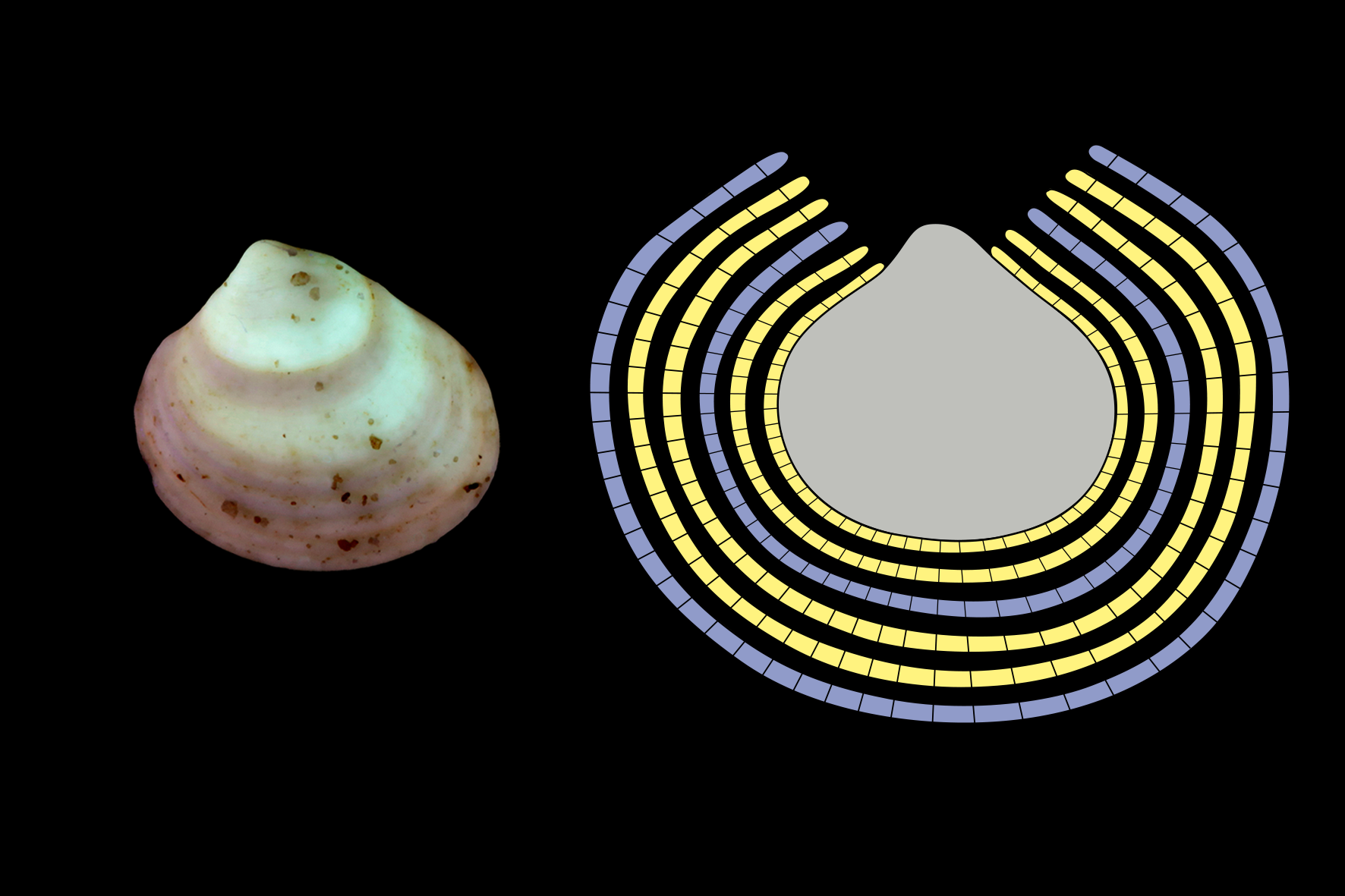

Supplement: S23 Fig — Pattern 1B: fluorescent commarginal stripes. (TIF) [file pone.0126745.s025.tif]

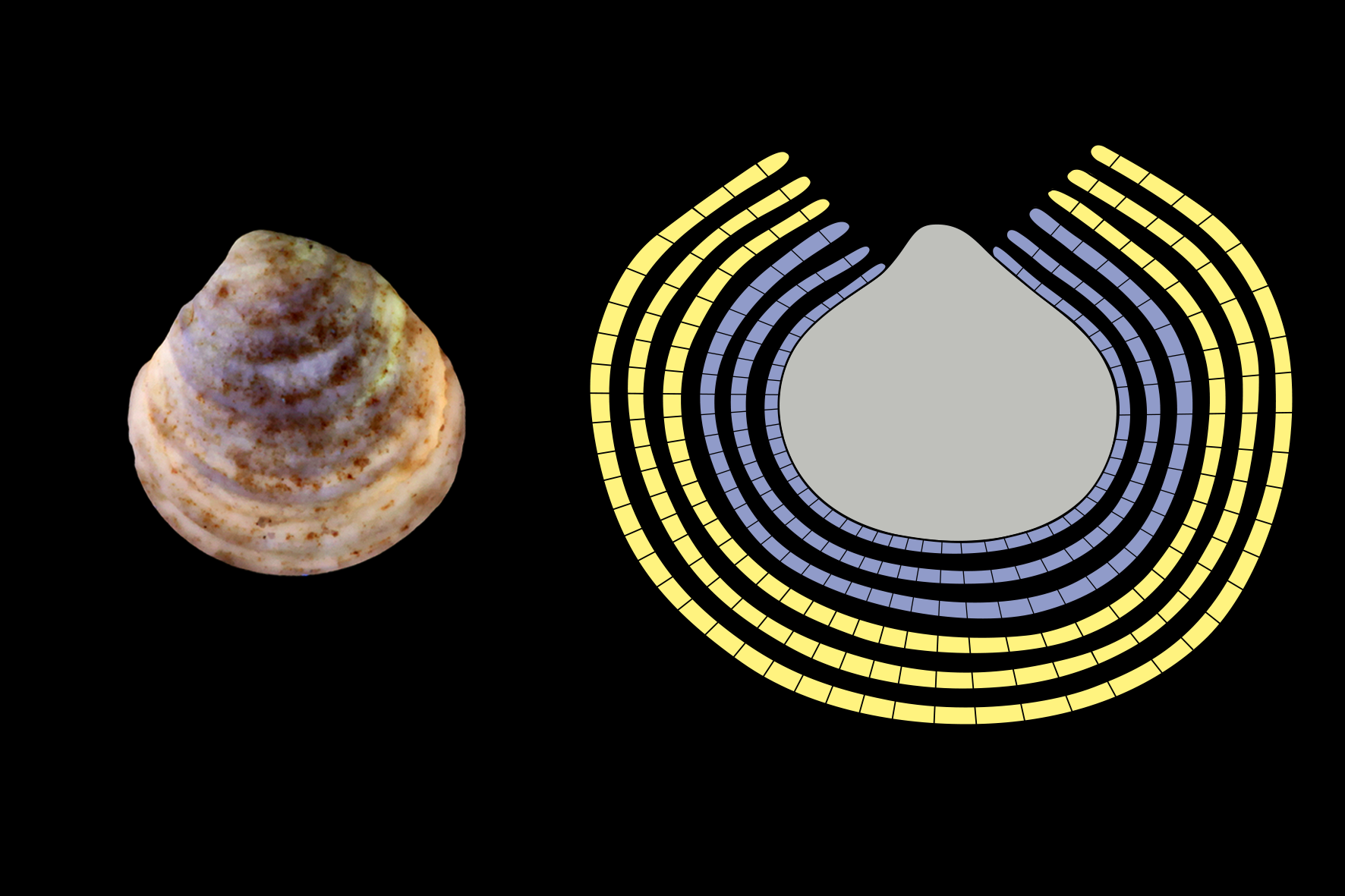

Supplement: S24 Fig — Pattern 1B: fluorescent commarginal stripes. (TIF) [file pone.0126745.s026.tif]

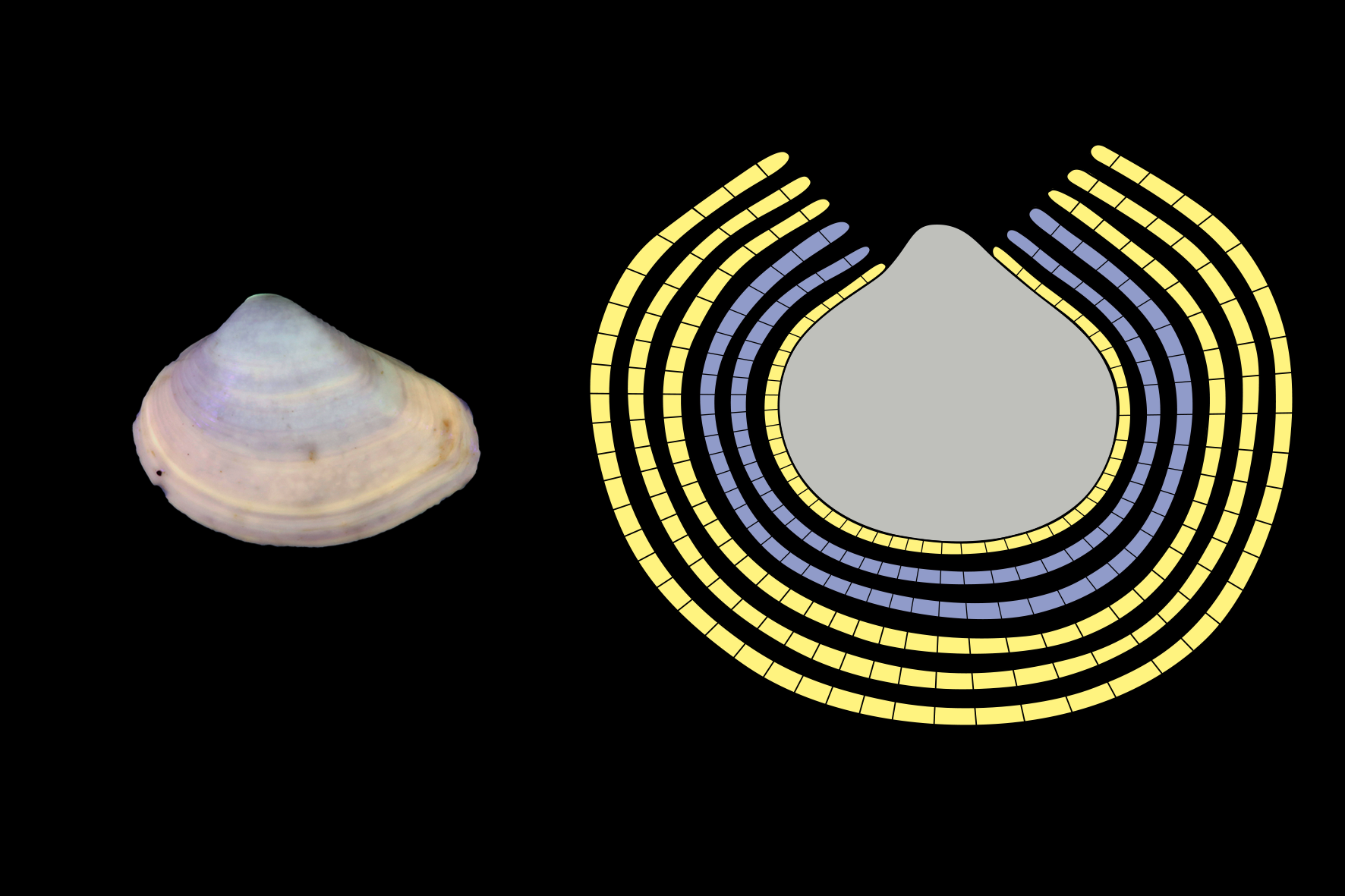

Supplement: S25 Fig — Pattern 1B: fluorescent commarginal stripes. (TIF) [file pone.0126745.s027.tif]
